# A Systematic Review of User Attitudes Toward GenAI: Influencing Factors and Industry Perspectives

**DOI:** 10.3390/jintelligence13070078

**Published:** 2025-06-27

**Authors:** Junjie Chen, Wei Xie, Qing Xie, Anshu Hu, Yiran Qiao, Ruoyu Wan, Yuhan Liu

**Affiliations:** 1 School of Design, Huazhong University of Science and Technology, Wuhan 430074, China; m202474533@hust.edu.cn (J.C.); m202474551@hust.edu.cn (W.X.); u202115710@hust.edu.cn (R.W.); 2 MoCT Key Laboratory of Lighting Interactive Service & Tech, Huazhong University of Science and Technology, Wuhan 430074, China; 3 School of Computer Science and Artificial Intelligence, Wuhan University of Technology, Wuhan 430070, China; felixxq@whut.edu.cn (Q.X.); anshuhu@whut.edu.cn (A.H.); 317909@whut.edu.cn (Y.Q.); 4 Cognitive Aesthetics Media Lab (CAMLab), Harvard Faculty of Arts and Sciences (FAS), Harvard University, Cambridge, MA 02138, USA

**Keywords:** GenAI, user attitudes, creativity, intelligence, acceptance, application domains, bibliometric analysis

## Abstract

In the era of GenAI, user attitude—shaped by cognition, emotion, and behavior—plays a critical role in the sustainable development of human–AI interaction. Human creativity and intelligence, as core drivers of social progress, are important factors influencing user attitudes. This paper systematically reviews 243 peer-reviewed studies on GenAI user attitudes published since 2019, identifying major research methods and theoretical perspectives, including the Technology Acceptance Model (TAM), the Unified Theory of Acceptance and Use of Technology (UTAUT), and the AI Device Use Acceptance (AIDUA) model. Drawing on contemporary creativity theories—such as Sternberg’s Theory of Successful Intelligence, the 4C Model by Kaufman and Beghetto, and the Dynamic Creativity Framework—we analyze how creativity and intelligence are conceptualized in current studies and how they affect user responses to GenAI. Through cross-cultural analysis and multimodal comparison, this review offers a comprehensive understanding of the interplay between GenAI and human creativity, aiming to support more inclusive and sustainable human–AI collaboration.

## 1. Introduction

### 1.1. Background

#### 1.1.1. GenAI

**Figure 1:**
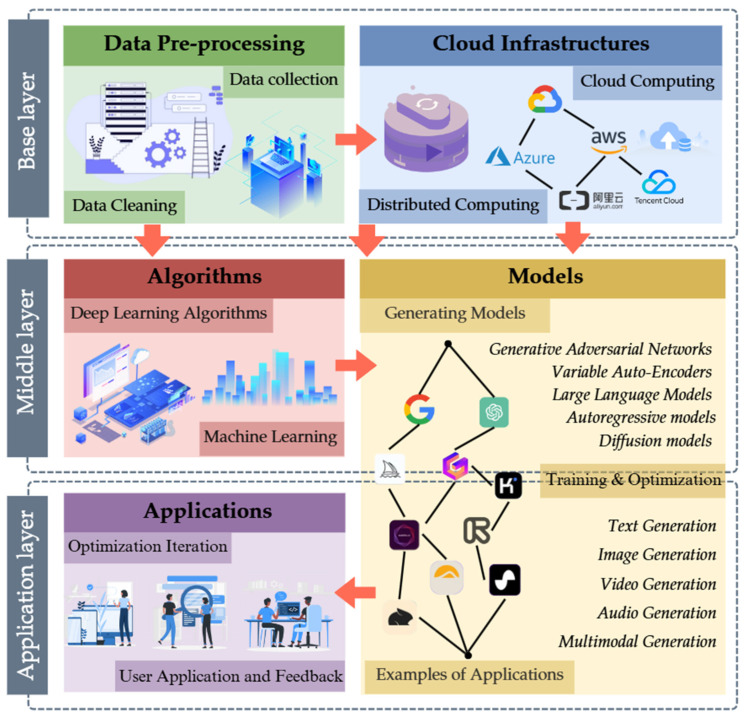
Flowchart of Generative Artificial Intelligence.

GenAI (Generative Artificial Intelligence), relying on advanced machine learning techniques and intense learning, enables deep parsing of multivariate data and innovative content generation (IBM 2024). Under this technological framework, models such as generative adversarial networks (GANs), variational autoencoders (VAEs), large-scale language models (LLMs), autoregressive models, and emerging diffusion models generate new data or make predictions Each of them, with its unique mechanisms, has driven breakthroughs in GenAI in the areas of text, image, video, audio, and multimodal content generation (McKinsey 2024). Figure 1 shows the system architecture of GenAI, starting from data preprocessing through the processing of algorithms and models and finally providing services to users at the application layer. The feedback from users can be used to optimize the algorithms again, forming a closed loop. The base layer is the data preprocessing layer. The middle layer mainly provides algorithms, models, and tools to make it easier for developers and researchers to build, deploy, and optimize GenAI models. The application layer is the end-user and industry application-oriented layer where GenAI is transformed into actual products and services. The scope of research in this review focuses on all user-oriented content.

#### 1.1.2. Attitude

Attitude is an accumulation of information regarding an object, person, situation, or experience leading to a predisposition to act positively or negatively towards some object or technology (Littlejohn and Foss 2010). User attitude usually refers to an individual’s overall evaluation or tendency towards a particular object, concept, or technology. User attitudes influence the acceptance, adoption, and continued use of a technology or service (Davis 1989; Ajze 1991). In this paper, attitude consists of three main components: (1) Cognitive component: beliefs or knowledge about the technology (perceived usefulness, ease of use) (Davis 1989). (2) Affective component: emotional reactions or feelings towards the technology (satisfaction, trust, acceptance) (Douglass 1977). (3) Behavioral component: intention or tendency to act in a certain way towards the technology (intention to use or recommend) (Ajze 1991; Venkatesh et al. 2003).

#### 1.1.3. Creativity and Intelligence

Sternberg’s Theory of Successful Intelligence defines intelligence as the capacity to achieve personal goals within one’s socio-cultural context through the balanced deployment of analytical, creative, and practical abilities. It also emphasizes a dynamic process of self-regulation—adapting to, shaping, or selecting environments to suit one’s needs (Sternberg 1999). Building on this foundation, Sternberg further proposes the notion of Adaptive Intelligence, which extends the Theory of Successful Intelligence by emphasizing long-term survival, ethical reasoning, and ecological responsibility as central to intelligent behavior (Staff 2020). However, the question of what exactly defines human intelligence is contested, particularly among researchers of artificial intelligence (Britannica 2025). Building on Sternberg’s Theory of Successful Intelligence—which posits that intelligence is not a fixed trait but a modifiable and context-dependent capacity—this study further conceptualizes human intelligence as a multidimensional system. It encompasses cognitive, metacognitive, emotional, creative, aesthetic, moral, and social dimensions, which interact dynamically to support learning, problem-solving, and value-driven behavior, particularly in technology-mediated environments (Creely and Blannin 2025; Kaufman and Beghetto 2009).

Human creativity has traditionally been defined as the ability to produce novel and valuable outcomes—a concept that encompasses innovation, originality, and reflective thinking (Runco and Jaeger 2012). Classical models, such as the Four-C Model, provide a developmental taxonomy that categorizes creativity into four levels: mini-c (personally meaningful insights), little-c (everyday problem-solving), pro-c (Kaufman and Beghetto 2009). This model underscores the continuum of creative expression, emphasizing that creativity ranges from individual, subjective experiences to achievements recognized on a societal scale.

In addition, recent research increasingly conceptualizes creativity as a dynamic, context-sensitive, and socially embedded process (Csikszentmihalyi 1998; Corazza 2016). On the one hand, creativity is understood as a potential and evolving process. Sternberg and Lubart’s (1995) investment theory conceptualizes creativity as a dynamic allocation of resources—such as intelligence, motivation, and domain knowledge—in interaction with environmental factors (Sternberg and Lubart 1995). Corazza (2016) defines creativity as “potential originality and effectiveness” and introduces the notion of creative inconclusiveness, which captures the inherently uncertain and emergent nature of creative processes (Corazza 2016). On the other hand, creativity is shaped by cultural and social contexts. proposed the Systems Model of Creativity, emphasizing that the generation of creativity is the result of dynamic interaction among persons, domains, and fields (Csikszentmihalyi 1998). Glăveanu conceptualizes creativity as an emergent, co-constructed process shaped through continuous interaction among individuals, tools, and socio-cultural environments and further emphasizes its nature as a negotiated outcome across cultural boundaries in his cultural dynamic creativity framework (Glăveanu 2014). Beghetto and Corazza (2019) emphasize the variability and complexity of creativity, proposing that it should be understood and assessed through a dynamic systems perspective, particularly in educational contexts (Beghetto and Corazza 2019). This dynamic perspective becomes particularly salient in GenAI environments, where human creativity interacts continuously with algorithmic outputs, evolving in response to both machine suggestions and human reinterpretations (Creely and Blannin 2025). Therefore, based on the Four-C Model and dynamic creativity frameworks, this study defines creativity as a multi-level and evolving capacity for generating original and effective outcomes, emerging through ongoing interaction between individuals, socio-cultural environments, and generative technologies.

In the context of GenAI, human intelligence, as a multidimensional adaptive system, not only drives users’ perceptions (e.g., credibility, perceived usefulness, and perceived risk of GenAI) but also guides their behaviors (e.g., actual use decisions, frequency of use, and co-creativity), and shapes their attitudes toward GenAI. In addition, the differences in user attitudes reflect, to some extent, the different cognitive assessments and emotional responses of users in different domains to “creativity and intelligence being affected by GenAI”. Therefore, this paper focuses on the state of human creativity and intelligence in various domains in the context of GenAI, as well as And how GenAI affects human creativity and intelligence.

### 1.2. Research Objectives

This review synthesizes current research on user attitudes toward GenAI, summarizing the predominant methodological approaches and theoretical frameworks while extending discussions on human creativity and intelligence. A meta-analysis was conducted on key dependent variables to examine the underlying mechanisms influencing user attitudes. Furthermore, the review explores how GenAI shapes human creativity and intelligence across different industries and how these changes, in turn, affect user perceptions. By comparing variations in attitudes and influencing factors across diverse user groups, the study further uncovers the roles of human creativity, intelligence, and user heterogeneity in the evolving landscape of GenAI acceptance.

## 2. Materials and Methods

### 2.1. Literature Retrieval

The search followed the Preferred Reporting Items for Systematic Reviews and meta-analysis PRISMA guidelines (Liberati et al. 2009). In this study, existing papers and publications on users’ attitudes toward GenAI were collected from Scopus and Web of Science databases (see Table 1 for keywords).

### 2.2. Literature Screening

**Figure 2:**
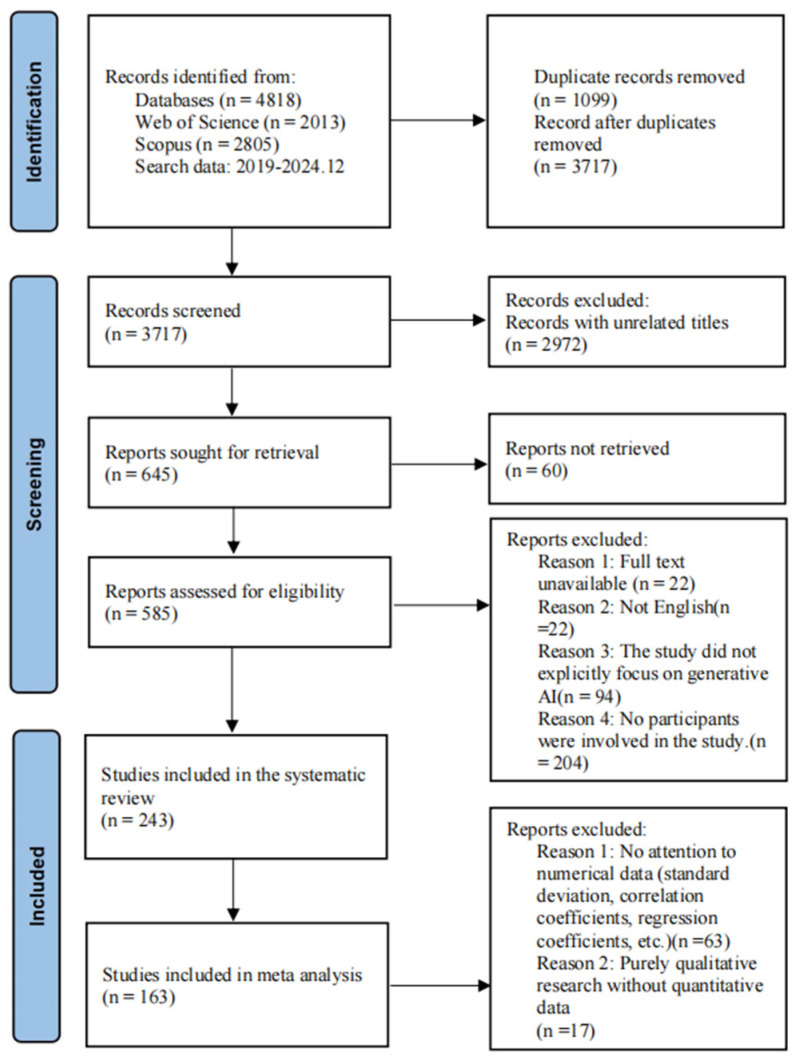
Flow Diagram of Literature Search.

The study initially collected 4818 papers and retained 3717 records in EndNote after removing duplicate entries. Subsequently, during Title-and-Abstract-based screening, literature not directly related to the topic of this study was initially eliminated. The screening requirements included (1) non-English literature, (2) literature that was clearly incompatible with the purpose of this study, (3) purely algorithmic studies that were not directly related to GenAI, and (4) studies that did not explicitly address the subjective feelings of users or human creativity. After this process, the amount of literature narrowed down to 745. To ensure the relevance and quality of the included literature, studies that (1) did not have full text, (2) did not have domain-specific user involvement studies, and (3) did not focus on attitudes, creativity, and intelligence (as defined by introduction) were excluded (see Table 2 and Figure 2 for a detailed screening process), and 243 articles were identified as the main analyzed subjects of this study. Of the 243 studies, 61 did not focus on numerical data (standard deviation, correlation coefficients, regression coefficients, etc.), and 9 did not have sufficient data to calculate effect sizes. A final total of 163 studies were identified for meta-analysis.

### 2.3. Data Extraction

The data extraction process was conducted in two stages. In the first stage, coding of study characteristics was performed, extracting key details such as publication year, sample size, domain, theoretical framework, and research methodology. The research methodology included both data collection methods and data analysis techniques. In the second stage, coding of effect size was conducted, extracting information on dependent variables, independent variables, effect sizes, statistical support for the relationships, and sample sizes. Notably, since a significant number of studies employed regression analysis and regression coefficients (β) are more appropriate for causal inference and evaluating the independent impact of independent variables on dependent variables (Cohen 2013), this study used regression coefficients (β) as the measure of effect size for the meta-analysis. (Additional details are presented in Supplementary Table S1).

## 3. Results

### 3.1. Research Methods

In this study sample, data acquisition methods were mainly quantitative (*n* = 169 (69.6%)). Among them, the most common research method was a survey or questionnaire (*n* = 150.89%). In contrast, the other 17 articles focused on experiments. The samples surveyed ranged from extensive samples (*n* = 3942 (Alsharhan et al. 2023)) to relatively small samples (*n* = 61 (Diirr et al. 2024)). Of the quantitative methods, 132 used models or theories to aid the research. 25% of the papers used qualitative methods (*n* = 45). These qualitative data came from interviews (semi-structured interviews (Amann et al. 2023)), focus groups (Maphoto et al. 2024), observations (Majid et al. 2024), and case studies (Zheng and Tse 2023), among others. The data were in non-numerical forms such as text, images, and audio. In addition, 10% of the studies used mixed qualitative–quantitative methods (i.e., combining qualitative and quantitative methods) to overcome the limitations of a single method (Turner et al. 2015). Table 3 details the specific quantities of various research methods.

In terms of the temporal dimension of data collection, most studies have used cross-sectional methods, which are also suitable for characterizing populations, measuring the prevalence of phenomena, and exploring associations between different variables (Bryman 2016). However, strong evidence of causality cannot be provided because time series are not measurable. A small number of articles conducted longitudinal analyses, which can be used to analyze changes in individuals or groups over time, revealing long-term trends and causal relationships (Ruspini 2000).

Questionnaires and cross-sectional designs provide a widely applicable basis for analysis, but over-reliance on questionnaires can lead to superficial attitude measurement, ignoring the emotional complexity of user attitudes and individual cognitive biases. In addition, users may tend to give “socially desirable” answers, resulting in self-report bias (Paulhus 1984), while cross-sectional designs cannot reflect the dynamic changes in attitudes that users experience with the process of use, technological upgrades, and changes in functionality (Zhou and Zhang 2024). For example, a user’s initial “exploratory” attitude may change to “dependence” or “resistance” after long-term use, which needs to be modeled by longitudinal regression and growth models.

### 3.2. Research Models and Theories

**Figure 3:**
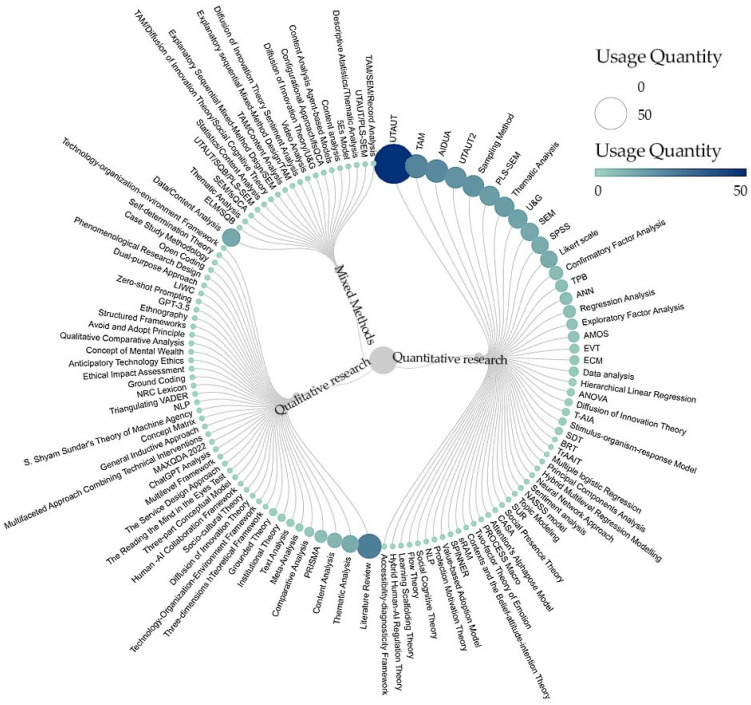
Data analysis method: Radial tree graph.

Quantitative research usually predicts the relationship between variables through existing models or theories. The models and theories used in research not only include traditional technology acceptance theories, such as the technology acceptance model (Davis 1989) and the UTAUT model (Venkatesh et al. 2003), but scholars also develop models according to their research purposes. For example, AIDUA (Gursoy et al. 2019) is a research model developed by Gursoy et al. in 2019 to study GenAI acceptance. The T-AIA model, a task-oriented AI device adoption model (Y. Yang et al. 2022), was proposed by Alexander et al. in 2023 to explain the factors behind AI’s credibility and acceptance of clinicians’ factors with the TrAAIT model (Stevens and Stetson 2023). These models are utilized to analyze the interactions between dependent and independent variables comprehensively and to explore how moderating variables may moderate these relationships in different contexts. Qualitative research, on the other hand, emphasizes a focus on individual experiences, motivations, and beliefs by enriching the details and contexts in the model or theory (Bryman 2016). Models, theories, and frameworks serve more as guidelines than as structures to be strictly followed. Therefore, models or theoretical frameworks in qualitative research may be continuously adapted during analysis to accommodate new findings (Heckman and Robb 1985). Figure 3 summarizes the methods used during the data analysis that assisted in dissecting the intrinsic connections between the factors and their mechanisms of influence on the acceptance of GenAI.

Table 4 lists the significant theories/models found in the literature studying attitudes about GenAI. It can be seen that UTAUT dominates the list with 50 studies. Other important models and theories include the TAM model, AIDUA model, UTAUT2, U&G theory, and TPB theory, among others. The results also show that about 9% of the analyzed studies do not rely on existing theories/models.

Some studies have introduced the theories and variables of creativity and intelligence. Such as the Four-C model (Kaufman and Beghetto 2009), the Innovation diffusion theory (Rogers et al. 2008), the Higher-order thinking Skills theory (Pellas 2025), and the Computational creativity theory (Franceschelli and Musolesi 2024) Among them, higher-order thinking skills (HOTS) list creativity along with critical thinking and problem-solving abilities as important components of human intelligence, and study the interrelationship between their attitudes towards the use of GenAI (Pellas 2025). Table 5 lists the important theories/models related to human creativity and intelligence in the research.

In addition, some studies have proposed modeling frameworks that link human creativity and user attitudes. Lim et al. proposed an interpretive model by combining the 4C model to describe and support the creative interaction between children and GenAI tools. This model centers on the "mini-c moment", and the proposed "creativity" model is defined as the ability of children to "generate novel and meaningful ideas" when interacting with GenAI. This kind of creativity is not only influenced by the functions of GenAI tools, but also by children’s cognition, emotion, motivation and environment (Newman et al. 2024). Margherita et al. proposed a framework for artificial intelligence to enhance creativity, dividing the impact of GenAI on creativity into three core categories: stimulating agile methods, enhancing human creativity, and stimulating unconventional thinking, providing a clear and structured perspective for studying user attitudes (Pagani and Wind 2025).

### 3.3. Meta-Analysis

A random-effects meta-analysis was conducted to synthesize existing empirical findings on user attitudes. The analysis focused on key dependent variables, including behavioral intention, use behavior, attitude, acceptance, trust, and concern, which collectively reflect the extent to which users are influenced to adopt GenAI. To obtain robust estimates of between-study heterogeneity, we employed the Restricted Maximum Likelihood (REML) method, which provides less biased estimates of variance—particularly in cases where the number of studies is small to moderate (Meta-Analysis Package for R n.d.).

#### 3.3.1. Dependent Variables

Dependent, independent, and moderating variables were extracted based on the 163 studies included in the review. Dependent variables are variables that are influenced by other variables (Alsharhan et al. 2023). Table 6 integrates the main dependent variables in the studies related to user attitudes in GenAI. Among them, research on behavioral intention is the dependent variable with the highest number of studies (*n* = 369), which is due to the fact that behavioral intention also reflects users’ mental attitudes and provides strong theoretical and practical support for understanding and predicting user behavior.

#### 3.3.2. Independent Variables

Independent variables are variables that affect other variables, and analyzing the independent variables in retrieved documents helps to understand the factors that influence users’ attitudes toward GenAI (Andrade 2021). In addition, this study refers to Krishnan et al.’s study for structural grouping of similar concepts (Gopinath and Kasilingam 2023), e.g., creativity with personal innovativeness (PI), individual creativity (IC), innovation orientation (IO), and perceived novelty have similar concepts. Therefore, they are all categorized as “creativity”.

Human intelligence comprises multiple dimensions—including cognitive, metacognitive, emotional, creative, aesthetic, moral, and social—which may influence users’ attitudes and behavioral intentions toward AI technologies through distinct psychological mechanisms. “Perceived” variables (e.g., perceived usefulness, ease of use, and intelligence) reflect rational evaluations of system functionality and efficiency and are thus considered external expressions of cognitive intelligence (Davis 1989; Facione 1989). In contrast, variables like perceived trust, enjoyment, and risk are tied to emotional experiences and expectations, falling under emotional intelligence (Gefen et al. 2003; Van der Heijden 2004; Featherman and Pavlou 2003). Self-efficacy and critical thinking represent users’ reflective thinking, self-regulation, and evaluative abilities, aligning with metacognitive intelligence (Facione 1989). Social influence and subjective norms reflect perceived conformity and normative pressure and are therefore associated with social intelligence. Although some studies mention variables related to moral and aesthetic intelligence (e.g., content quality, interface design), these have not been included in the meta-analysis due to limited sample sizes.

This heterogeneity in the nature of the variables suggests that when analyzing user attitudes, a functional distinction should be made between the psychological mechanisms behind “perception” to avoid a simplified understanding of human intelligence. Based on human intelligence, Table 7 summarizes the main independent variables.

#### 3.3.3. Meta-Analysis Results

Since the retrieved literature spans a large period, the year of publication was used as an independent variable in this study to analyze whether the year moderates the strength of the effect of IV (Perceived Usefulness and Perceived Ease of Use) on DV (Behavioral Intention).

Table 8 revealed that the independent variable significantly predicted effect size, F (1, 107) = 16.947, *p* < 0.001. However, the moderator variable “Year” was not statistically significant, F (1, 107) = 0.402, *p* = 0.528, indicating that publication year did not moderate the effect and that the explanatory power of the independent variable remained consistent across the observed period.

To account for the increased risk of errors due to multiple comparisons across numerous statistical tests, a stricter significance threshold of *p* < 0.01 was adopted in this study. This adjustment follows recommendations from peer review and standard practices in meta-analytical research involving large-scale comparisons.

Table 9 shows that Perceived Usefulness (PU) (b = 0.323, *p* < 0.001) was a significant positive predictor of effect size. In contrast, Perceived Ease of Use (PEU) (b = 0.184, *p* = 0.019) was not significant. The publication year (2019–2024) also did not significantly influence the effect size (*p* = 0.528), suggesting that the year does not play a moderating role in the relationship between independent variables and effect size.

In this study, we conducted a univariate meta-analysis of the key independent variables that influence users’ attitudes and behaviors toward GenAI. Table 8 presents the results of the meta-analysis between different dependent and independent variables, including the effect size analysis (i.e., estimate significance level and confidence intervals) and the results of the heterogeneity test (see Table 10).

Among the independent variables affecting behavioral intentions, Perceived Usefulness, Trust, Self-efficacy, and Perceived Enjoyment exhibited medium effect sizes (0.2 < β < 0.5), and Perceived Ease of Use, Social Influence, Facilitating Conditions, and Creativity presented small effect sizes (β < 0.2), and all of them reached statistically significant levels. The perceived risk presented a very small negative effect on behavioral intention and was not significant (β = −0.0686 *p* = 0.042).

For the prediction of usage behavior, all independent variables have different levels of positive effects, and behavioral intention presents a significant and strong effect size on usage behavior (β > 0.5), but the effect sizes of social influence, attitude, and creativity are not significant.

Among the independent variables predicting user attitudes, perceived usefulness, trust, social influence, perceived hedonism, perceived value, personal innovativeness, and perceived ease of use have positive effects, and perceived ease of use, creativity, and facilitating conditions have insignificant effect sizes. And perceived risk out significant negative effect (−0.142, *p* < 0.01).

For the prediction of acceptance, attitude was the strongest positive variable (β = 0.553, *p* < 0.001), while trust (β = 0.438, *p* < 0.001) and perceived usefulness (β = 0.419, *p* < 0.01) also showed strong effect sizes, with all three being highly statistically significant. None of its independent variables were significant.

Social Influence, Perceived Enjoyment (β = 0.192, *p* = 0.19), and Perceived Ease of Use (β = 0.176, *p* = 0.119) were statistically insignificant, although they showed a positive effect size on trust.

Perceived Risk had a significant positive effect on worry (β = 0.486, *p* < 0.001), followed by Perceived Usefulness (0.246, *p* = 0.009). The effects of social influence and perceived ease of use were not significant.

This suggests that the greater the individual’s confidence in their abilities, the more likely they are to exhibit higher levels of creativity. In contrast, the effect of behavioral intentions on human creativity was relatively weak but still statistically significant (β = 0.414, *p* = 0.007).

Table 11 shows that the *p*-values are all less than 0.001, and the I^2^ values for all variables are more than 80%, indicating that there is a high degree of heterogeneity with large differences between studies.

#### 3.3.4. Subgroup Analysis Results

To explore potential moderators that might account for the observed heterogeneity, subgroup analyses were conducted based on application domains (G. Li et al. 2021). These domains include education, creative industry (e.g., design, art, innovation-driven sectors, and technologies), healthcare, organization (e.g., applications related to employees, internal enterprise operations, HR processes, and managerial decision-making), consumer service scenarios (e.g., mobile banking, restaurants, tourism, hospitality, insurance, and online shopping), and general-purpose use. This categorization was adapted from the framework proposed by (Kelly et al. 2023), which provides a structured lens to distinguish GenAI adoption across diverse sectors.

According to methodological guidance, subgroup analyses should not be performed when there are fewer than three studies in a given subgroup (Borenstein et al. 2021). Therefore, only subgroups with three or more studies were included, using Behavioral Intention (BI) as the focal outcome variable. Table 12 summarizes the total effects of each explanatory factor on BI across different sectors.

In domains such as education and service industries—where user bases are broad and technological penetration is relatively high—factors including perceived usefulness, ease of use, social influence, trust, attitude, and perceived playfulness all exhibit significant positive effects on usage intention. In contrast, in more specialized or structurally complex fields—such as the creative industries, healthcare, and organizational settings—facilitating conditions and perceived usefulness play a more central role. Specifically, the adoption of AI in creative sectors is primarily driven by the instrumental utility of the tools rather than by ease of use or social influence.

### 3.4. Application Domain

The study categorizes research into several domains: Education (*n* = 125, 51%), Creative Industry (*n* = 36, 15%), Healthcare (*n* = 21, 9%), and Commercial Organizations (*n* = 20, 8%). Each domain contains insights into human creativity under the influence of GenAI, along with related variables. The study is structured around the current state of human creativity influenced by GenAI, target population attitudes toward GenAI, factors influencing these attitudes, and differences in attitudes across populations within the same field.

#### 3.4.1. Education

**The state of creativity and intelligence under the influence of GenAI:**

In the dimension of intelligence, several models have expanded the introduction of variables such as “perceived intelligence (Bhaskar et al. 2024)”, “self-efficacy (Fu et al. 2024)”, and “higher-order cognitive regulation (Pellas 2025)”, providing evidence that users’ cognitive and metacognitive intelligence are associated with variations in their attitudes toward GenAI.

In the dimension of creativity, research indicates that GenAI effectively stimulates and enhances students’ creative potential by facilitating cognitive engagement and divergent thinking processes (Shahzad et al. 2024). However, excessive reliance on AI-generated content may constrain students’ original expressive space, weakening their creative motivation and individual style (L. D. Li et al. 2024). Students develop creativity and creative learning skills, particularly when solving problems beyond their prior knowledge, especially through pedagogical strategies that promote knowledge construction and problem-solving (Rajan and Niranjan 2025). Intrinsic motivation is thus considered a key driver of creative development. Moreover, studies have shown that creativity is significantly positively correlated with Higher-Order Thinking Skills—such as academic achievement, problem-solving, and critical thinking—and can positively influence students’ attitudes toward machine learning (Pellas 2025), which aligns with the findings of this meta-analysis.

**Attitudes of target groups towards GenAI and influencing factors:**

Users of GenAI mainly include educators and students, covering both general and higher education scenarios. Established studies overall show that students generally exhibit more positive attitudes compared to educators, but this trend varies somewhat across studies.

On the student side, as shown in Table 13, personal factors such as students’ perceptual intelligence (Bhaskar et al. 2024), curiosity (Gao et al. 2024), and information literacy (Jose et al. 2024), and technical factors such as the relative advantages of GenAI, compatibility, observability, and trialability (Ibrahim et al. 2023; Paraskevi et al. 2023) positively affect user attitudes. Notably, several studies have shown that personal innovativeness (PI) is recognized as an important moderating variable. Several studies have found that students with higher personal innovativeness are more inclined to evaluate ChatGPT positively, but it is also moderated by other factors (e.g., educational background, cultural differences, and technological familiarity (Tian et al. 2024). However, the moderating effect of personal innovativeness also varied from study to study, which is also consistent with the results of the meta-analysis. For example, in the Sri Lankan study, the moderating effect of personal innovativeness was not significant, although it was considered a moderator (Nawaz et al. 2024). In contrast, in a study in the Philippines, personal innovativeness had a significant positive effect on behavioral intentions (Hernandez et al. 2023).

On their part, educators are relatively cautious in their attitudes, even though they usually have adequate knowledge of GenAI tools (Kamoun et al. 2024). Several studies have pointed out that educators are more likely to express concerns about content authenticity and pedagogical effectiveness when confronted with AI teaching applications. In addition, they are also more sensitive to the ethical issues (academic integrity risks and bias output, etc.) that GenAI may raise in teaching scenarios (L. D. Li et al. 2024; Hidayat-ur-Rehman and Ibrahim 2023). As summarized in Table 13, educators’ negative attitudes are often associated with perceived risk variables, including factors such as psychological risk (Wu et al. 2022), ethical risk (Zhu et al. 2024), potential risk (Zhu et al. 2024), and over-reliance (Abu Hammour et al. 2023). Meanwhile, individual differences (e.g., teaching experience (X. Lin et al. 2024), cultural background (Yusuf et al. 2024)), institutional policies (Polyportis and Pahos 2024), and geography (Yusuf et al. 2024) are also important moderators of teachers’ attitudes.

A comparison of student and educator attitudes toward GenAI reveals that educators as a whole have more negative attitudes and lower levels of trust. Students were more concerned with performance expectations and social influence and were susceptible to surrounding evaluations, while educators were more concerned with perceived playfulness. Student educators were more independent in their attitudes and less swayed by external factors (Kamoun et al. 2024). In addition, in higher education, there are significant differences in attitudes toward GenAI among students of different majors. Specifically, art and design students were more aware of the protection of originality and privacy of their works due to their unique career orientation. They were more concerned about the potential alternative effects of GenAI on their future careers (W. Li 2024).

On the other hand, veterinary students were more receptive to GenAI, believing that AI is powerful but cannot completely replace veterinary needs and recognizing its potential to assist in learning and practice (Worthing et al. 2024). In economics, factors such as interactivity, perceived trust, and performance expectations positively influenced behavioral intentions to use ChatGPT, but the effects of ethics and effort expectations were not significant (Salifu et al. 2024). In legal education, social support is a key factor influencing learner motivation, which cannot be adequately provided or replaced by current LLM education (Sağın et al. 2023).

#### 3.4.2. Creative Industry

**The state of creativity and intelligence under the influence of GenAI:**

Although GenAI shows high performance and “creative” performance in image generation, text creation, product concept design, etc., its nature still relies mainly on existing data for reorganization and generation, which easily leads to the homogenization of results. In contrast, human designers’ innovative design capabilities based on emotion and empathy, abstract creativity, and personalization are difficult to replace by GenAI (J. Li et al. 2024).

Studies have shown that experienced designers perceive GenAI as an aid to help accomplish repetitive tasks, thus freeing up more time for creative work. Whereas for junior designers, the help of GenAI is more obvious, but over-reliance on GenAI may lead to the degradation of creativity (Doshi and Hauser 2024).

Related experiments have shown that in image generation tasks, works prompted by professional artists are rated as more creative overall, followed by AI prompts, and finally by novice prompts. There have also been experiments comparing user preferences for AI-created versus human-created work (Micalizzi 2024; Ragot et al. 2020), human and chatbot in terms of creativity performance (Koivisto and Grassini 2023), and user ratings of creative work when AI and humans are the creators (Magni et al. 2024). These experiments support Guilford’s idea of the “enduring value of human creativity” (Seli et al. 2025). It also reflects that creativity is not only embodied in the output but also in the path of conceptualization, cultural judgment, and expression strategies. These process characteristics reflect important dimensions of human intelligence, such as metacognition, emotion, aesthetics, and cultural understanding. Therefore, the creator should be the subject of the creation rather than the editor.

**Attitudes of target groups towards GenAI and influencing factors:**

In the creative practitioner community, despite the AI learning anxiety of some users, they still tend to use generative AI techniques (Yin et al. 2023). It has been suggested that designers may experience an “intermittent neutralization” phenomenon during actual use (Zhou and Zhang 2024). That is, the designers’ acceptance and adoption of generative AI are affected by privacy concerns and information illusions, leading to cognitive dissonance, which further leads to intermittent meshing. Whereas perceptual intelligence, anthropomorphism, and personalization can play a positive role, thereby preventing the spiking behavior. According to the summary in Table 14, factors such as effort expectation, price value, and hedonic motivation in UTAUT positively affect creators’ willingness to use GenAI, while performance expectation has no significant effect on willingness to use generative AI (Yu et al. 2024; Y. Wang and Zhang 2023). Creators are gradually realizing the opportunities presented by AI, suggesting that their adoption of the technology is based on actual functional performance rather than judgments based only on potential impact.

The acceptance of GenAI by the consumer group is significantly influenced by the values of “anthropocentrism” (Kokkoris et al. 2023). Controlled experiments show that when a piece of art is labeled with artificial intelligence, people’s preferences will decrease (L. Gu and Li 2022; Sohn et al. 2020; H.-Y. Kim and Lee 2023). Consumers usually tend to think that products designed by humans are more artistic and unique, while products generated by generative AI may seem mechanical and lack soul due to technological intervention (Ragot et al. 2020). However, this prejudice against AI art is not irreversible. Research shows that it is becoming increasingly difficult to distinguish between artificial art and AI art, and consumers’ acceptance of AI works will increase due to the improvement in interactivity and participation (Latikka et al. 2023). These findings imply that consumers’ attitudes may be associated with their expectations of autonomy and creative engagement rather than being solely linked to the aesthetic presentation of the work.

#### 3.4.3. Healthcare

**The state of creativity and intelligence under the influence of GenAI:**

Due to the specificity and complexity of the healthcare field, although GenAI can provide data support through simulation and modeling, its essence is still based on the output of existing data and algorithms and lacks true autonomy and creativity (Duffourc and Gerke 2023). For example, applications of GenAI in healthcare include the generation of diagnostic reports, treatment recommendations, and assisted analysis of medical research, but these outcomes still rely on the professional judgment and ethical responsibility of physicians (Shahsavar and Choudhury 2023). In addition, doctors need to establish an emotional connection with patients and provide personalized care, which is difficult for AI to replace. Therefore, the role of GenAI in healthcare is more like an “extension tool” than a creative subject, and its “intelligence” is more of a functional intelligence than a creative intelligence with human perception and judgment. Moreover, some studies suggest that the increasing reliance on AI tools may compromise biological intelligence, disrupting abstraction, creativity, and critical thinking (Sharma and Sarode 2024). Therefore, in the medical field, human creativity still dominates, while GenAI plays more of a supporting role.

**Attitudes of target groups towards GenAI and influencing factors:**

The attitude of users in this field is relatively positive, and both patients and healthcare workers are increasingly inclined to enable the use of generative AI in healthcare settings, even if the technology is not specifically designed for healthcare (Shahsavar and Choudhury 2023). Among these, trust is the most studied dependent variable, while information accuracy, transparency, security, and ethical principles are important factors affecting trust.

Healthcare practitioners are generally positive about the use of generative AI in healthcare, particularly recognizing its potential to improve the accuracy, speed, and efficiency of medical decision-making (Amann et al. 2023). Studies have shown that 70% of psychiatrists believe that generative AI can improve the efficiency of document processing, but the majority of physicians do not believe that generative AI can completely replace direct physician–patient communication (Blease et al. 2024)while expressing reservations about its ability to make ethical judgments (Abu-Farha et al. 2023).

Patients’ attitudes toward generative AI are relatively more complex. On the one hand, patients will have a positive attitude towards generative AI technology because of the smarter, more refined, and humanized healthcare services it brings. On the other hand, patients have equally high demands on the accuracy and transparency of generative AI, and they want to obtain reliable and accurate information to support their health decisions. In addition, privacy and data security are also important concerns for patients, and any technological application that may disclose personal health information may cause them to worry (Xu and Wang 2024). Among the moderating factors, the higher the complexity of health-related tasks, the lower the level of patient trust (Nov et al. 2023). Table 15 summarizes the independent and moderating variables affecting user attitudes in the healthcare domain.

#### 3.4.4. Organization

**The state of creativity and intelligence under the influence of GenAI:**

Employee creativity in an organization is the generation of novel and useful ideas, which is essential for the organization to be flexible and successful in responding to the dynamic market environment. GenAI is currently being used in the innovation ideation phase as well. Studies have shown that AI-generated ideas are comparable to or even better than the results of expert workshops in terms of novelty but still fall short in terms of perceived feasibility. This reveals the need for organizations to maintain a balance between “breakthrough ideas” and “practical feasibility” when relying on AI for innovation (Füller et al. 2024).

In addition, human-like features (e.g., perceptual intelligence and anthropomorphism) and enhanced social network connectivity have a positive impact on employee creativity, contributing to the quality of human–computer interactions, which in turn improves creativity (Zhang et al. 2024). Although technological convenience lowers the threshold and increases utilization, it may also prompt users to be superficial, thus inhibiting deep learning and the formation of true creativity, with perceived ease of use playing a double-edged role in this process (Zhang et al. 2025).

In addition, GenAI gradually becomes an external extension of human creativity in project management, content creation, and decision support scenarios. It was found that innovation attitude, peer influence, task–technology fit, and self-efficacy significantly influence employees’ creative behavior in using AI tools (B. Yang et al. 2024).

**Attitudes of target groups towards GenAI and influencing factors:**

Managers tend to have a positive attitude towards GenAI, especially when recognizing its potential to enhance efficiency, productivity, and innovation. Although personal characteristics such as age, gender, and education are often considered control variables in previous studies, these factors do not show a significant impact on the willingness to use GenAI in enterprises, whereas the experience factor of running a business is particularly important (V. Gupta 2024). The study showed significant differences between entrepreneurs and managers on four personality dimensions, with entrepreneurs scoring higher on responsibility and openness to experience and lower on neuroticism and agreeableness (Zhao and Seibert 2006).

GenAI can improve efficiency and employee effectiveness, and employees recognize that GenAI can assist them in doing their jobs (Koo et al. 2021). However, research has focused on employees’ negative, fear-based, and threat-centered attitudes toward GenAI (Bankins et al. 2024). This is related to factors conclude replacement concerns (Alexiou and Lingmont 2020; Suseno et al. 2022), outdated skills (Golin and Innocenti 2022), workflow changes (Zhao and Seibert 2006), and decreased work engagement (Arias-Pérez and Vélez-Jaramillo 2022). Still, some employees choose to see GenAI as a challenging stressor rather than a hindrance (Ding 2021). Employees with higher motivation show more positive attitudes toward technology, which may help them adapt to and utilize GenAI (Tyers and Leonard 2021).Table 16 summarizes the independent and moderating variables affecting user attitudes in the healthcare domain.

In addition to the above-mentioned application scenarios, the study also includes the areas of online shopping, travel services, hotel services, restaurant services, mobile services, and software development, among others. Table A1 summarizes the impact variables in these areas.

### 3.5. Bibliometrix Review

#### 3.5.1. Overall Trend of Publications and Citation

**Figure 4:**
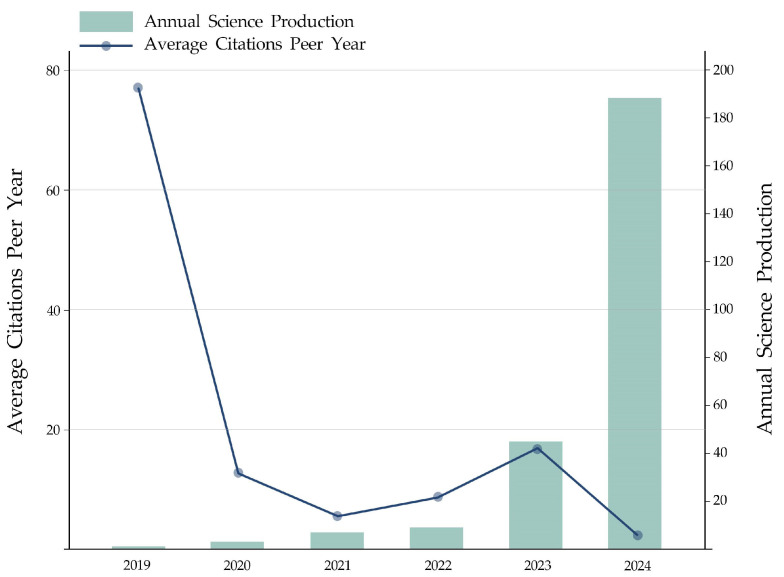
The overall trend of publications and average citations. Annual publication trends and average citations per paper of the research on users’ attitudes towards GenAI in the Web of Science Core Collection (WoSCC) database from 2019 to 2024 were analyzed using Bibliometrix 4.4.1.

According to Figure 4, from 2019 to 2024, the number of articles about users’ attitudes toward GenAI and the factors influencing them shows a continuously increasing trend. Based on the number of publications and their growth rate, the overall situation can be divided into two parts: 2019–2022 is the initial development phase of this study, and 2022–2024 is the steady and rapid growth phase. This vast change is due to technological advancements, wide application, and problems brought by GenAI. In 2023, GenAI technologies (e.g., OpenAI’s GPT-4, MidJourney, etc.) have made significant breakthroughs, which are capable of generating high-quality content such as text, images, and code. These technologies are not only widely promising but also raise a series of policy and ethical issues, thus triggering a wide range of academic concerns, which is also in line with the content analysis part of this study.

#### 3.5.2. Author Collaborative Network Analysis

**Figure 5:**
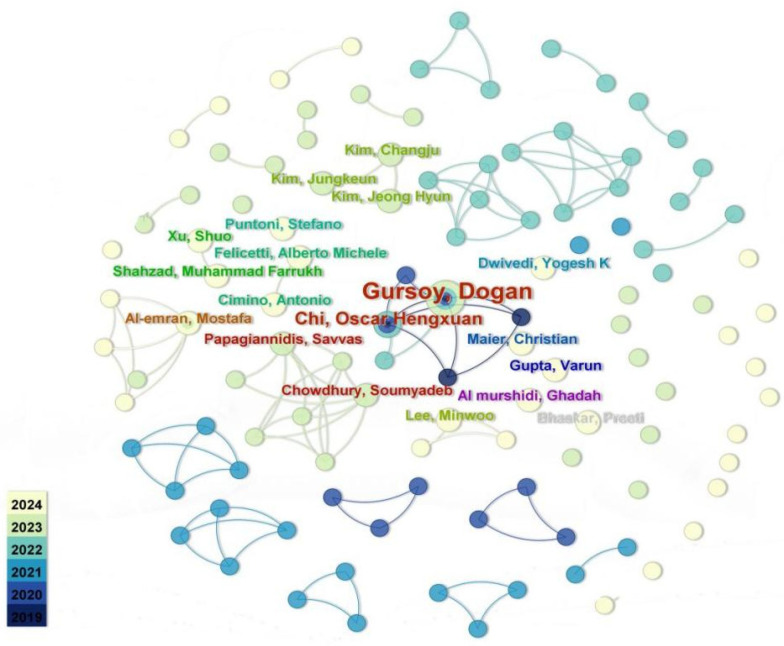
The collaboration network of authors. Each node in the map represents an author. The size of a node reflects the articles that authors have published. According to the legend in the lower left corner, the various colors in the node represent different times when the article has been published. The line indicates the period of collaboration between the authors. Different colored fonts represent different clusters. Analysis performed by using CiteSpace 6.3.

Based on Price’s Law, core authors contributing to research on GenAI attitudes and influencing factors were identified. The author collaboration network includes 113 nodes and 100 links. As shown in Figure 5, overall author connectivity is limited, indicating weak collaboration in the field. One of the most connected authors is Gursoy D (State University System of Florida), forming a large central node linked to Chi, Oscaer Hengxuan, Lin Hongxia, Ribeiro Manuel Alector, and others. Additional networks center around Lee, Paoagiannidis, AL-Emran M, and Kim J. Node colors in Figure 5 suggest a notable increase in contributing authors in recent years.

In addition, according to Table 17. Realistic Gursory D has the highest number of publications (5) and the total number of citations (817), which is the first place in the comprehensive data. Moreover, he has been working on user acceptance modeling for AI since 2019 and has made outstanding contributions to the research in this field. In addition, Balakrishnan J and Choudhury S are able to buy a high TC value despite their low volume and short posting time.

#### 3.5.3. Analysis of Contribution of Institutions and Countries

Table 18 presents the top 10 institutions and countries with the highest number of publications on user attitudes toward Generative AI (GenAI) from 2019 to 2024. Several universities in the United States have made significant contributions to this field, notably the University of California system, the State University System of Florida, and Harvard University, positioning the US at the forefront of GenAI research. Swansea University from the UK also stands out as one of the most active institutions in this domain, engaging in collaborations that span multiple countries, including the USA, UK, India, and China. In Asia, Beijing Normal University and the Chinese University of Hong Kong reflect the growing involvement of mainland China and Hong Kong in GenAI research.

**Figure 6:**
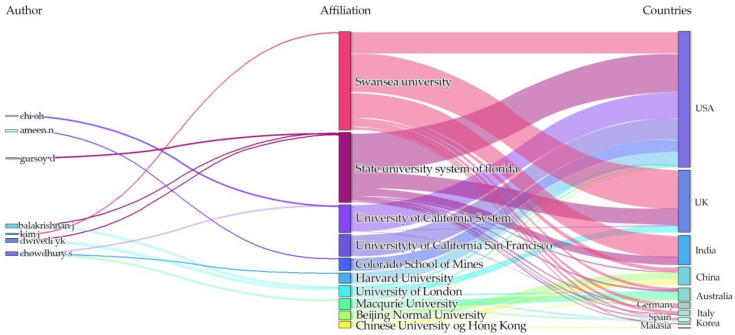
Affiliation relationships between authors and countries. Tree-field plot of affiliations (items 10), authors (items 7), and countries (items 10) produced by using Bibliometrics.

To complement this statistical overview, Figure 6 provides a visual representation of the relationships among authors, their institutional affiliations, and corresponding countries through a Sankey diagram. The visualization highlights the global but uneven distribution of GenAI research, with a concentration in Western countries and increasing participation from Asia and other regions. It also reveals active cross-institutional and cross-national collaborations, suggesting the interdisciplinary and international nature of research on user perceptions of GenAI. This underscores the need for more inclusive and culturally diverse studies to broaden the understanding of user attitudes toward GenAI technologies across different social contexts.

**Figure 7:**
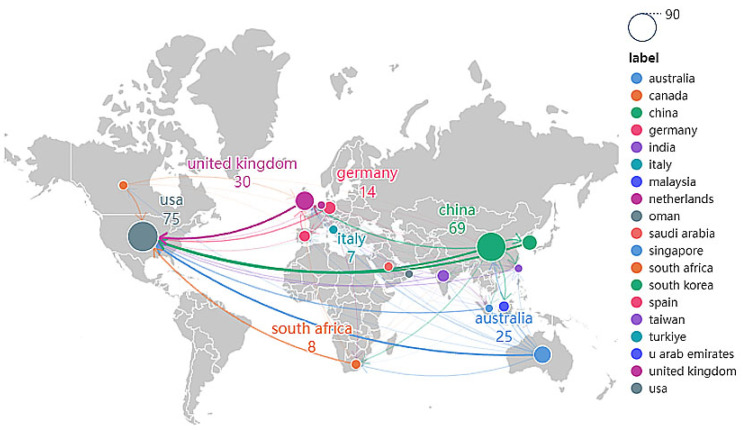
Country and region collaboration map. Analysis was performed by using Scimago Graphica.

**Figure 8:**
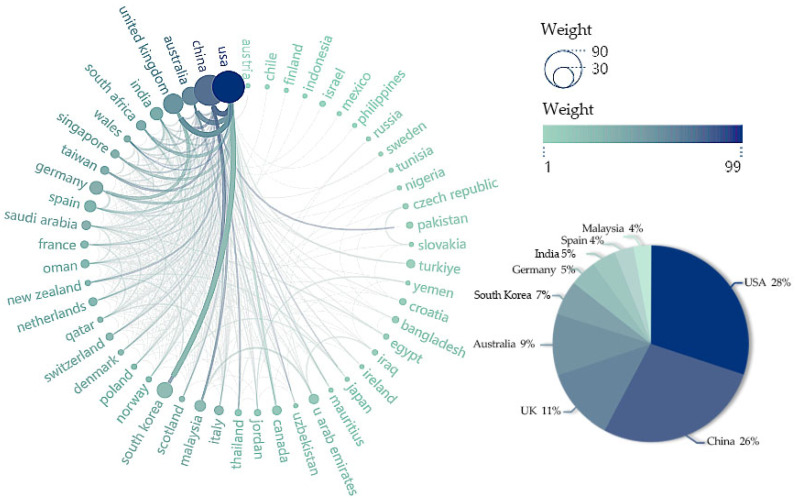
Country cooperation chord charts and country production pie charts. Analysis was performed by using Scimago Graphica and VOSviewer 2.1.5.

The country cooperation map, Figure 7, illustrates the cooperation between countries/regions and the global distribution of the collaboration. The nodes on the world map represent the countries, with larger nodes representing a higher number of outputs and the number of research outputs for each country labeled next to them. According to Figure 7 and Figure 8, it is shown that the US, China, and the UK are the countries with the highest research outputs in the field of modification, 75 (28%), 69 (26%), and 30 (11%), respectively. In addition, the thickness of the lines linking the countries represents the strength of the cooperation between the countries, with a thicker line representing stronger cooperation. Figure 7 shows that there is strong cooperation between different countries and institutions, and cross-country and cross-school research cooperation is evident, especially the collaboration between the United States and other countries is remarkable. Among them, the most frequent connection is between the United States and the United Kingdom, with 12 collaborations. This is followed by the United States and China, as well as the United States and South Korea, both with a frequency of 10 collaborations. The US and Australia ranked third, with a frequency of nine times. Due to limitations in the map display, the collaboration between all countries studying this area is shown in more detail in Figure 8.

**Figure 9:**
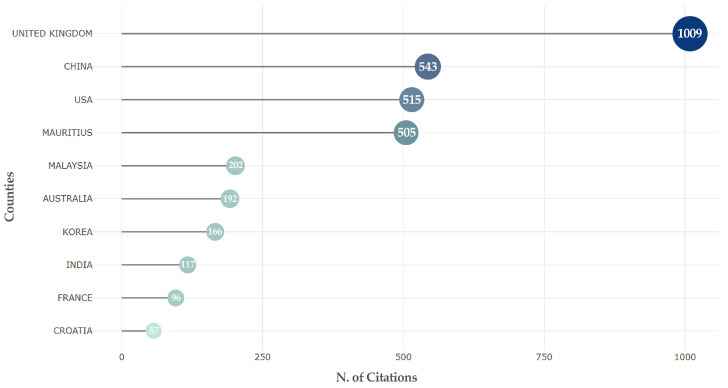
Most Cited Countries.

Figure 9 shows the citation situations of articles in various countries. In terms of academic influence, the UK has a strong academic influence, with 1012 total citations and an average article citation of 48.2. Interestingly, Mauritius, which has only two publications, has 505 total citations and an average citation as high as 252.5. China, on the other hand, has a relatively large number of papers, but the centrality of its documents is relatively low, with an average citation of only 9.7, which suggests that China should improve its academic impact by improving the quality of its research on users’ attitudes towards GenAI. Meanwhile, although Malaysia has fewer articles (10), the average citation is 50.5. On the one hand, this is due to the fact that tourism is a popular scenario regarding GenAI applications, and Malaysia has conducted more research on tourism. On the other hand, it also shows the academic influence of Malaysia in this field.

**Figure 10:**
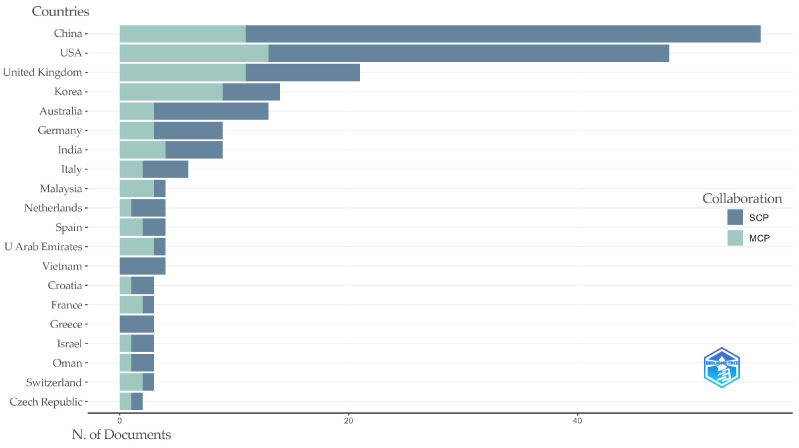
Corresponding authors’ countries. MCP: multiple-country publications; SCP: single-country publications. Analysis performed using Bibliometrix 4.4.1.

According to Figure 10, only 19.6% and 27.1% of publications in China and the United States, respectively, were accomplished through multinational cooperation. In contrast, several countries, such as New Zealand, Singapore, and Thailand, have a 100% cooperation rate.

#### 3.5.4. Publication Co-Citation Analysis

**Figure 11:**
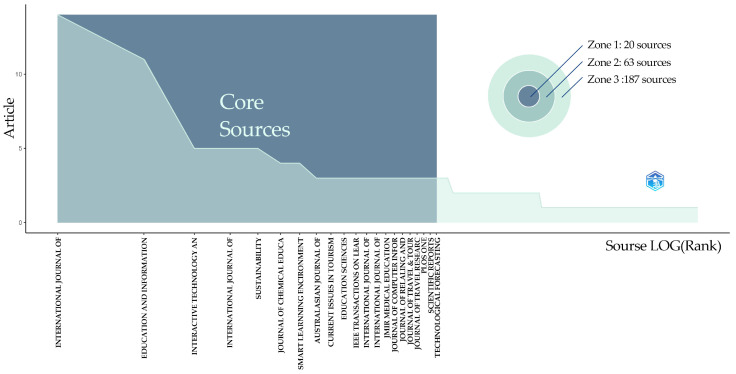
Distribution of Core Journals Based on Bradford’s Law.

Figure 11 illustrates the core sources based on Bradford’s Law, identifying journals with the highest number of GenAI-related publications. The *X*-axis ranks journals by publication frequency and impact (logarithmic scale), while the *Y*-axis shows the number of articles. As expected, the number of publications declines with journal rank, aligning with Bradford’s Law, which states that a few journals account for most publications.

Using Bradford’s Law through Bibliometrix, we identified the most active journals in the dataset, revealing that research is concentrated in a few key journals rather than evenly distributed. This concentration helps to identify core journals in the field of video generation. According to Bradford’s Law, data is typically categorized into three regions: core, middle, and peripheral sources, and our data follows this pattern. Figure 11 visualizes the sources into three regions: region 1 (core sources), region 2 (intermediate sources), and region 3 (peripheral sources). The shaded area labeled “Core Sources” denotes Region 1, which contains a small number of journals that contribute most of the relevant literature. Of the 174 journals included in this study, 20 fall into Region 1. Region 2 consists of 63 intermediate sources. These journals publish a moderate number of articles, and although they are not as productive as the core sources, they still provide a valuable contribution to the field. Region 3 represents peripheral sources and contains 89 sources with the fewest published articles. The contribution of these sources is small compared to the core and intermediate regions.

The study identified a minimum citation threshold of 20, and 133 out of 6813 sources reached this threshold. In the visualization image, each node represents a journal, and the size of the node represents the number of citations in the journal. The curves represent co-citation links between journals, and the thickness of the curves represents the co-citation strength of the linked journals. In addition, the color of each node is based on the cluster to which it belongs.

**Figure 12:**
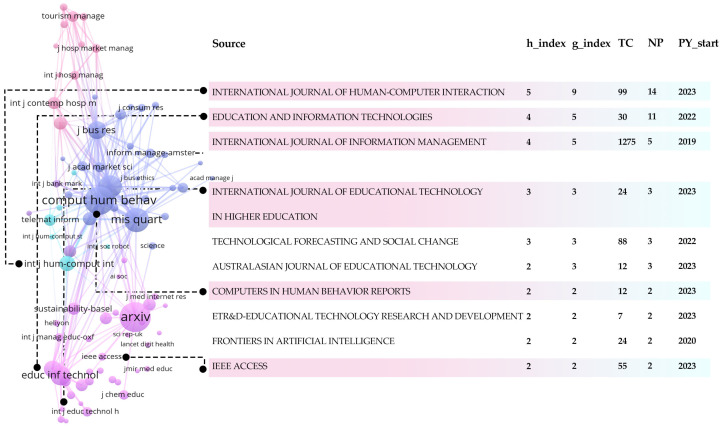
Publications co-citation network. Analysis was performed by using Scimago Graphica. and VOSviewer 2.1.5.

Figure 12 highlights how research on Generative AI is spreading rapidly across education, human behavior, and computing, with increasing interdisciplinary overlap. Key journals and publication metrics can help guide researchers toward influential outlets and networks for contributing to this dynamic field.

#### 3.5.5. Hot Spots and Trend Analysis

**Figure 13:**
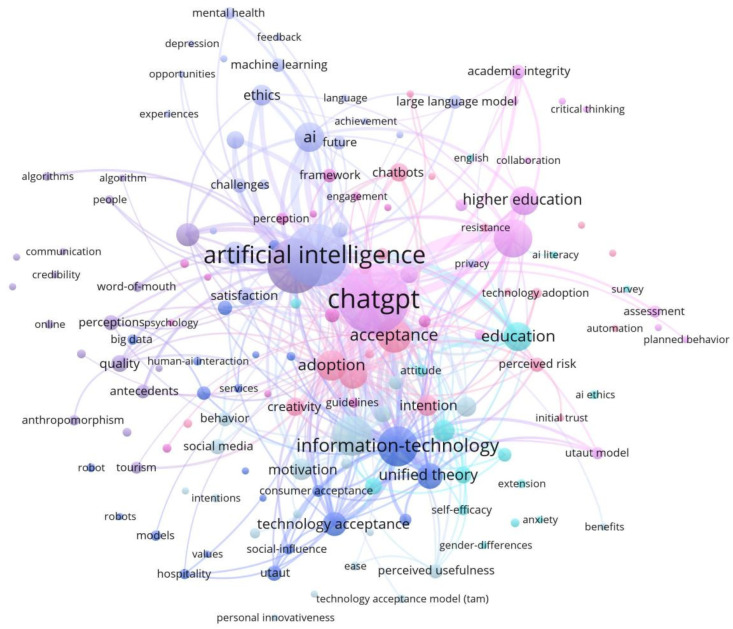
Keyword Co-occurrence Network.

Keywords reflect the core content of the papers and help Miyagi Snowflake You Network analyze research frequency and interrelations, thus identifying hotspots and future trends. As shown in Figure 13, among 1279 keywords, 79 appear at least three times. Node size and link strength indicate keyword frequency and co-occurrence, while colors represent different clusters. The keywords fall into four main categories: (1) technology-related (e.g., “ChatGPT,” “information-technology,” “large language model”); (2) user attitude (e.g., “acceptance,” “intention,” “trust”); (3) domain-specific (e.g., “education,” “design,” “higher education”); and (4) research model-related (e.g., “TAM,” “UTAUT”).

**Figure 14:**
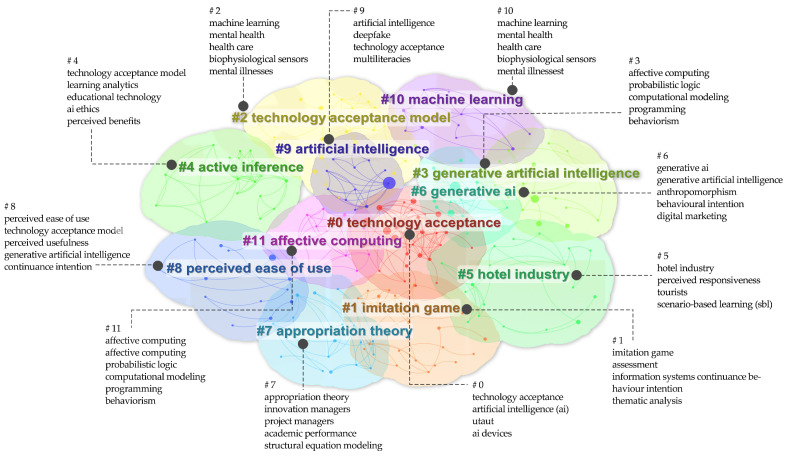
Keyword clustering diagram. That is, all the keywords retrieved in this study are divided into clusters. Each cluster has a different color and label. The smaller the label number, the more keywords the cluster contains. Analyzed using CiteSpace 6.3.

Mean Silhouette S = 0.8614 > 0.7. Modularity Q = 0.5965 > 0.5. The values of Q and S are within a reasonable range, which indicates that this clustering is convincing. The exact color keywords in Figure 14 are the same clusters, which form a total of 11 clusters, and these clusters reflect the research hotspots of users’ attitudes towards GenAI.

**Figure 15:**
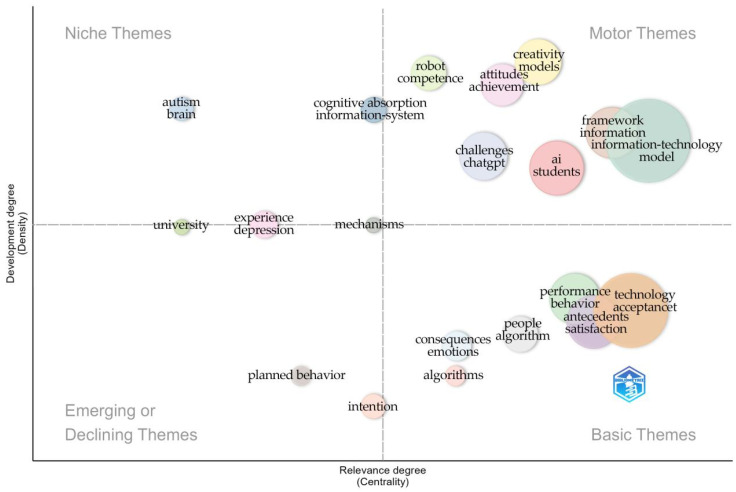
Thematic evolution map.

Figure 15 summarizes the distribution of topics in a specific domain, including the current “Niche Themes” and “Emerging or Declining Themes.” The particular themes cover a wide range of areas, from creativity, robotics, and cognitive science to education, mental health, and artificial intelligence. These themes reflect current research priorities and steady interest within the field. At the same time, the charts also point to emerging or declining trends that indicate new directions for future research or shifts in existing research directions. This analysis helps to understand the dynamics of research within the field and guides strategic planning and resource allocation.

## 4. Discussion

### 4.1. Comprehensive Analysis

#### 4.1.1. Theoretical Model

Traditional models such as TAM and UTAUT are mainly applicable to standardized, instrumental, function-oriented digital platforms. It has been noted that the TAM model lacks generalizability across industries (Kelly et al. 2022), and the UTAUT model focuses on the adoption of various digital landscapes rather than smart technologies (e.g., e-learning, mobile payments, and e-banking) (Tian et al. 2024), which has limitations of applicability across different cultural contexts and industries (Jadil et al. 2021). In addition, the models emphasize technological attributes and environmental factors, neglecting the influence of individual characteristics (e.g., self-efficacy, innovativeness) and affective factors (e.g., trust, anxiety) on technology acceptance (Dwivedi et al. 2019). It shows the limitations of these models when facing complex, intelligent technologies, especially generative AI with cognitive capabilities, effective design, and human–computer interaction features.

AIDUA and TrAAIT have begun to integrate irrational variables such as “anthropomorphism” and “emotion”. The anthropomorphism in the AIDUA model is not applicable to non-anthropomorphic AI technologies such as ChatGPT, and new variables such as perceived humanness and novelty value need to be introduced to more accurately describe user acceptance behavior (X. Ma and Huo 2023).

GenAI exhibits significantly different user acceptance paths in several industries, such as healthcare, education, creative design, and finance, among others; thus, user heterogeneity must be emphasized. For example, in the medical scenario, the TrAAIT model proposes a trust-oriented AI acceptance path for clinicians, emphasizing psychological assessment factors such as “clinical value, system reliability, and information trustworthiness” (Stevens and Stetson 2023). In creative scenarios, research should pay more attention to individual differences such as “creativity identity, sense of autonomous expression, and trait curiosity”. In addition, different users may interact with AI devices in different ways, and the T-AIA model proposed by Yang et al. indicates that there are significant differences in the attitudes and behavioral motivations of users towards social and task-based AI devices (Y. Yang et al. 2022). Utilitarian motivation, interaction convenience, and task–technology fit are important prerequisites for reducing users’ willingness to switch from task-oriented AI devices to humans (V. Gupta 2024).

Therefore, the future model should pay more attention to the difference between the functional value and the emotional value of AI types and introduce variables such as “task fit”, “interaction convenience”, and “emotional projection” in order to enhance the structural flexibility and contextual adaptability of the model. In addition, a “customizable variable module” can be added to maintain a dynamic balance between theoretical consistency and explanatory power in different industries. A variety of theories and methods are comprehensively applied to reveal the internal mechanism of GenAI acceptance in a comprehensive and in-depth manner.

#### 4.1.2. Variable Analysis

Among the independent variables included in this study, perceived usefulness, perceived value, and trust consistently played a significant positive role across multiple paths. It is worth noting that perceived ease of use did not show a significant effect in multiple paths, especially in the paths affecting attitude, acceptance, and trust, which did not reach statistical significance. Venkatesh et al. also mentioned that when the majority of the users perceive technology as “easy to use”, the variable loses its ceiling. Venkatesh et al. also mentioned that when the majority of users perceive technology as “easy to use”, the variable will lose its ceiling effect, thus affecting its statistical significance (Venkatesh and Bala 2008).

In addition, creativity appears as both a dependent and an independent variable in the model. When used as a dependent variable, an individual’s self-efficacy has a significant effect on it. When used as an independent variable, the effect of creativity on behavioral intentions reached statistical significance despite a smaller effect size; however, its effect on actual use behavior did not show a significant relationship. This result suggests that creativity is more of an intrinsic driving force that affects the level of user intention rather than a determinant that directly drives behavioral performance. The current Meta-analysis of the creativity path has a small sample size (k = 8), and the results are still limited in terms of robustness and representativeness. Future research should expand the sample base and introduce more mediating mechanisms to clarify further the status and role of creativity in the AIGC acceptance model.

Most of the variables involved in the study are essentially derived from users’ cognitive judgments, emotional responses, value expectations, and social motivations, i.e., human intelligence. Cognitive intelligence has a significant impact on users’ behavioral intentions and attitudes, especially perceived usefulness. Metacognitive intelligence significantly enhances the user’s behavioral intention through the enhancement in self-efficacy. Users’ confidence in their ability to operate the AI system, reflect, and self-assess helps to promote their usage behavior. Emotional intelligence plays an important role in several dimensions, with perceived trust and perceived pleasure positively influencing users’ attitudes and behavioral intentions, while perceived risk decreases users’ usage intentions. Social intelligence has a lower degree of influence relative to other types of intelligence and is not significant on some paths. These variables reflect not only the mental representations of individuals when using technology but also how human intelligence intervenes in the process of technology evaluation, contextual understanding, and behavior generation. Therefore, the acceptance mechanism of AIGC is also characterized as “the cognitive response of human intelligence to artificial intelligence”. This perspective can help to expand the TAM/UTAUT model and provide theoretical insights into the acceptance of AIGC technology in diverse industries and cultural contexts.

#### 4.1.3. Industry Perspective

In the context of evolving generative AI, users in different domains show significant status quo differences and characteristics in terms of creativity and intelligence.

In the field of education, GenAI is widely used as a cognitive tool. Some studies report that it is associated with enhanced intellectual support and perceived creativity, while others raise concerns about potential associations with weakened critical thinking and reduced creative motivation (L. D. Li et al. 2024; Shahzad et al. 2024). Studies have noted that the student population has an overall more positive attitude, positively influenced by factors such as curiosity, information literacy, and self-efficacy, while the teacher population is more cautious, often focusing on issues such as ethical risks and content accuracy. Creativity is closely related to higher-order cognitive skills in this domain, and an individual’s intrinsic motivation is key to creative development (Pellas 2025; Rajan and Niranjan 2025).

Creativity in this domain is often discussed in relation to higher-order cognitive skills, and intrinsic motivation has been frequently identified as an important factor associated with creative development. However, its creation is still based on the reorganization of data, which may tend to homogenize. Creativity in this field emphasizes Aesthetic and Emotional Intelligence, and GenAI is more often seen as a supporting role than a creative subject.

In the medical field, GenAI acts as an auxiliary intelligence rather than a creative subject (Duffourc and Gerke 2023). Healthcare professionals are concerned about its accuracy, ethics, and reliability, while patients value its convenience (Xu and Wang 2024; Amann et al. 2023). While AI tools are increasingly used to support efficiency in healthcare, emotional communication, and clinical judgment continue to be viewed as essential competencies of human practitioners, highlighting the distinct role of human intelligence in this field (Blease et al. 2024).

In organizations, employee creativity is influenced by factors such as perceived intelligence, social interaction, and task–technology fit. Ease of use has been discussed as a factor that may both encourage adoption and be associated with reduced depth of cognitive engagement (V. Gupta 2024; Zhang et al. 2025). Managers are generally positive, valuing their efficiency and innovation potential, while employees are polarized between perceived incentives and substitution anxiety. As GenAI applications expand beyond efficiency toward supporting human intelligence and creativity, organizations are also encountering new challenges related to creative outsourcing, skill degradation, and ethical considerations (Füller et al. 2024).

### 4.2. Limitations

This study still has several limitations that need to be emphasized and overcome in future research. First, in addition, most of the existing studies are cross-sectional in design, focusing on users’ short-term attitudes and behaviors toward GenAI and lacking dynamic tracking and mechanism identification of long-term changes in usage behaviors. In terms of theoretical foundation, current research still mainly relies on traditional technology acceptance models (e.g., TAM, UTAUT), lacking a systematic research framework that can reflect the heterogeneity of users (creativity, emotion, etc.), cultural heterogeneity, and global comparative perspectives.

Second, at the statistical level of meta-analysis, paths were generally characterized by high heterogeneity (significant I^2^ values), indicating systematic differences between studies beyond sampling error, such as study design, cultural context, sample characteristics, etc. In this context, the pooled effect sizes may not accurately represent the overall trend, and there is an urgent need to explore potential moderators through subgroup analysis further to enhance the explanatory power and generalizability of the results.

In addition, there are still obvious deficiencies in the type of content generated and the representativeness of the study sample. Currently, GenAI studies are highly focused on text generation tools (e.g., ChatGPT), which account for about 74% of the studies, while user acceptance studies on image (22%), video (3%), and audio (1%) generation tools are still scarce. With the rapid development of multimodal content generation, future research needs to more comprehensively cover all types of generation forms and their differentiated impact paths.

In terms of sample structure and cultural diversity, the existing literature mainly focuses on a single industry or region, with cross-industry studies involving only limited expert opinions (Dwivedi et al. 2023) or differences in trust (Novozhilova et al. 2024). Concerns about employment substitution and ethical issues are mostly scattered in individual field studies (Watters and Lemanski 2023). Particularly noticeable is the significant lack of cross-cultural research, with only a very few studies exploring differences in users’ intentions to use GenAI tools (e.g., applications in hospitality services) across cultures (Chi et al. 2023). It is noteworthy that the same influencing factor may show completely different directions and degrees of significance in different social situations and cultural contexts, reinforcing the importance of global comparative studies.

From the bibliometric analysis, it is evident that current publication output in the field of GenAI is highly geographically concentrated. The United States, China, and the United Kingdom collectively account for over 60% of total research output, reflecting structural imbalances in global academic resource distribution and discourse dominance. Meanwhile, countries such as Mauritius, despite contributing few publications, exhibit unusually high citation counts, suggesting the presence of small-sample, high-impact outliers that may distort influence assessments. Although the field shows a degree of internationalization, collaboration remains fragmented (113 nodes, 100 links), potentially leading to knowledge silos and hindering the development of coherent research paradigms. Additionally, Bradford’s Law analysis indicates that around 12% of core journals carry the majority of publications in this domain, reflecting a high concentration of academic discourse within a limited number of publishing platforms.

### 4.3. Future Research Directions

Future research should adopt a more dynamic longitudinal research design, establish a three-stage observation model of “short-term adaptation—medium-term dependence—long-term substitution or synergy”, track the evolution of user attitudes and behaviors over time, and identify key behavioral transformation nodes and risky points. Identify key behavioral transition points and risk generation points. This will help to understand the deep-rooted change mechanism of the relationship between users and GenAI.

At the same time, the current research bias on text generation tools should be addressed to expand the focus on multimodal generation tools such as image, video, and audio. Different generation modes bring different interaction methods, cognitive needs, and user expectations, which may form heterogeneous acceptance paths. In the future, cross-modal comparative studies should be carried out to analyze the determinants of commonalities and idiosyncrasies.

In terms of theoretical construction, the model should move from “instrumental acceptance” to an “intelligent interaction” paradigm, integrating cognitive assessment, emotional motivation, and social motivation and constructing a modularized theoretical framework to achieve contextual adaptability and structural flexibility of the model in healthcare, education, and creative design scenarios. We have constructed a theoretical framework that can be modularized and configured to achieve contextual adaptation and structural flexibility in medical, educational, and creative design scenarios. For example, the introduction of variables such as “task fit”, “interactivity”, and “affective projection” strengthens the explanatory power of the model in complex usage situations. In addition, future research needs to further emphasize the dual roles of “creativity” and “affective variables” in the acceptance mechanism, especially in the fields of creative design and knowledge generation, and explore how individuals can redefine their creative identities and value attributions through collaboration with AI.

In order to enhance cross-cultural adaptability, future research can construct localized acceptance path models based on regional cultural characteristics, such as focusing on instrumental efficacy and collective orientation in East Asian contexts and emphasizing autonomy and creative value in European and American cultures. This would enhance the geographic elasticity and global applicability of the theoretical models and further examine the influence of culture on the cognitive boundaries of creativity and the distribution of human–computer intelligence weighting.

Finally, future research should continue to focus on the ethical issues and legal risks of generative AI in the process of content generation—such as privacy protection, content authenticity, and copyright attribution, among others (M. Gupta et al. 2023)—to provide a theoretical basis for the formulation of relevant policies and laws, reduce the negative impact of technology on users’ attitudes, enhance users’ positive perceptions of generative AI, and realize better “human–computer co-creation”. Table 19 presents several potential future research directions in this domain.

## 5. Conclusions

This study systematically reviewed the relevant literature on user attitudes toward GenAI since 2019 and utilized systematic literature review, meta-analysis, and bibliometrics to systematically sort out the theoretical models, influencing factors, and industry differences in user attitudes.

Traditional technology acceptance models (e.g., TAM, UTAUT) remain the core framework, but their explanatory power for affective variables is limited. Emerging models (e.g., AIDUA, TrAAIT) integrate variables, such as “anthropomorphism” and “ethical risk”, and partially investigate theories related to creativity (Four C’s Model, Diffusion of Innovation Theory, Computational Creativity Theory, etc.). However, they need to be further adapted to cross-industry and cross-cultural scenarios.

Observations at the industry level show that students generally have a positive attitude, while teachers are concerned about academic integrity; creativity is closely related to Higher-Order Thinking Skills, and senior designers tend to use GenAI as an assistive tool; healthcare professionals emphasize technological accuracy, while patients value convenience and privacy protection, and there are significant differences in the acceptance of GenAI among different industry subjects.

Future research should break through the limitations of the current methodology, expand the multimodal and multicultural research perspectives, further improve the theoretical models, and continue to reflect on the roles and boundaries of human creativity and intelligence in the era of generative AI in order to promote the sustainable and inclusive development of human–computer collaboration.

## Figures and Tables

**Table 1:** Search Terms.

Topics	Keywords
Generative Artificial Intelligence	“AIGC” OR “Generative AI” OR “GenAI” OR “AI-generated content” OR “GANs”
AND	
User Attitude	user attitude” OR “acceptance” OR “perception” OR “behavior” OR “trust” OR “emotion” OR “reaction” OR “anxiety” OR “creativity” OR “concern” OR “intention” OR “Satisfaction”

Note: Quotation marks were used around terms that consisted of two or more words to ensure that results were returned that included these phrases rather than articles that contained each word individually.

**Table 2:** Inclusion and Exclusion Criteria.

	Inclusion Criteria	Exclusion Criteria
**Publication Type**	Original research published in peer-reviewed publications	Narrative reviews, letters, editorials, commentaries, unpublished manuscripts, meeting abstracts, and consensus statements
**Case definition**	The research must address users’ attitudes toward GenAI and their intention to use it.	The participants had no apparent attitudes or behavioral tendencies
**Dependent variable**	Usage intention, acceptance, adoption, willingness, trust	Other dependent variables
**Publication period**	From January 2019 to December 2024	Before 2019
**Publication language**	English	English translation is not available

**Table 3:** Methods of collecting ideas.

Research Paradigm	Data Collection Method	Number of Studies (%)	Total (%)
quantitative methods	Survey or questionnaire	150 (89%)	169 (69.6%)
	Experimental design	19 (11%)	
qualitative methods	Interview	16 (37%)	43 (17.6%)
	focus group	4 (9%)	
	observation	4 (9%)	
	Case study	11 (27%)	
	Media	8 (18%)	
Mixed Methods	Combined method	31 (12.8%)	31 (12.8%)

**Table 4:** Top 10 Models and Theories for Studying User Attitudes Toward GenAI.

#	Theory/Model	Number of Studies
1	Unified Theory of Acceptance and Use of Technology (UTAUT)	50
2	Technology acceptance model (TAM)	27
3	Artificially Intelligent Device Use Acceptance (AIDUA)	18
4	Unified Theory of Acceptance and Use of Technology2 (UTAUT2)	15
5	Use and Gratification (U&G)	14
6	Theory of Planned Behavior (TPB)	13
7	Self-Determination Theory (SDT)	10
8	Social Cognitive Theory (SCT)	8
9	Diffusion of Innovations Theory (DIT)	8
10	Stimulus–Organism Response (SOR) Theory	5

**Table 5:** Top 10 Models and Theories for Studying Human Creativity Variables.

#	Theory/Model	Number of Studies
1	Diffusion of Innovations Theory (DIT)	8
2	Unified Theory of Acceptance and Use of Technology2 (UTAUT2)	7
3	Unified Theory of Acceptance and Use of Technology (UTAUT)	6
4	Technology acceptance model (TAM)	6
5	Higher-Order Thinking Skills (HOTS)	4
6	Cognitive Appraisal Theory (CAT)	4
7	Social Cognitive Theory (SCT)	2
8	Use and Gratification (U&G)	2
9	Four-C Model	1
10	Computational Creativity Theory (CCT)	1

**Table 6:** Frequency of studies on the top ten dependent variables.

#	Dependent Variable	Definition	k	Count
1	Behavioral Intention	Desire to use technology in the future (Davis 1989)	369	77
2	Use Behavior	Use Behavior refers to the actions and patterns exhibited by individuals or groups when they utilize a product, service, technology, or system (Davis 1989)	94	37
3	Attitude	The individual’s positive or negative evaluation of performing the behavior (Ajzen 1985)	106	33
4	Acceptance	Individual or group perceptions and positive adoption intentions for technology, product, or innovation (Davis 1989)	67	25
5	Trust	Perception of confidence against the technology’s integrity and reliability (Morgan and Hunt 1994)	63	22
6	Concern	Individuals are concerned about the potential negative impacts or risks of a technology, product, or system (S. S. Kim et al. 2004)	42	14
7	Satisfaction	Customers’ emotions based on their expectations and consumption experience (Oliver 1977)	39	12
8	Continuance Intention	The extent to which consumers who have used a product or service in the past are willing to continue using the product or service in the future (M. Kim and Chang 2020)	41	11
9	Anxiety	Individuals experience anxiety, tension, or restlessness due to uncertainty or potential negative consequences when using new technologies or systems (Higgins and Compeau 1995)	7	5
10	Purchase intention	The willingness to purchase the product in the future and the customer’s willingness to buy the product further (Nuanchaona et al. 2021)	6	2

Note: k: Total number of studies in the dataset involving altered quantities and number of effectors; Count: Total amount of literature.

**Table 7:** Frequency of studies on the top ten independent variables.

Variable	Definition	Similar Constructs (Alias)	k
Perceived Usefulness	The degree to which a user expects a particular technology to enhance their performance by its use (Davis 1989)	Performance Expectancy, Relative Advantage	133
Perceived Ease of Use	The degree to which a user expects to use a technology free of effort (Davis 1989)	Effort Expectancy	116
Social Influence	The extent to which consumers perceive that others (e.g., family and friends) believe they should use a particular technology (Venkatesh et al. 2003)	Social norms, Social Need, Subjective Norms, Social Presence	96
Perceived Risk	The potential negative consequences or uncertainties that users associate with engaging with GenAI (Dinev and Hart 2006)	Perceived Ethical Risk, Perceived Anxiety	76
Facilitating Conditions	Consumers’ perceptions of the resources and support available to perform a behavior (Venkatesh et al. 2003)		64
Perceived Enjoyment	The extent to which the individual perceives that their attention is focused on the interaction with the technology, is curious during the interaction, and finds the interaction intrinsically enjoyable or interesting (Moon and Kim 2001)	Perceived enjoyment, Hedonic Motivation, Entertainment	61
Self-efficiency	The individual’s belief in their ability to effectively interact with and utilize generative AI tools to achieve specific creative, analytical, or operational goals (Davis 1989)	Competency Levels, Perceived Competence	54
Trust	Perception of confidence against the technology’s integrity and reliability (Morgan and Hunt 1994)	Perceived Trust, Perceived Credibility	50
Attitude	A person’s degree of evaluative effect (like or dislike) towards a target behavior (Ajzen 1985)		41
Perceived Value	The subjective evaluation by users of the usefulness, relevance, quality, and benefits derived from content generated by artificial intelligence (Chan and Zhou 2023)	Expected Benefits, Price Value, Perceived Benefits	41
Creativity	Persons or processes are creative to the extent that they produce creative products, and a product is creative if it meets two conditions: novelty and value (Vigeant 2024)	Personal Innovativeness, Perceived Creativity, and Individual Creativity	41

**Table 8:** Effect size meta-regression terms tests.

Variable	F	df_1_	df_2_	*p*-Value
Year	0.402	1	107	0.685
IV (Independent Variable)	16.947	1	107	<0.001

**Table 9:** Meta-regression results for PU and PEU of use intention (with Year as Moderator).

Independent Variable	Estimate	Standard Error	t	df	*p*	95% CI	
						Upper	Lower
Perceived usefulness	0.323 ***	0.076	4.233	107	<0.001	0.172	0474
Perceived Ease of Use	0.184	0.077	2.379	107	0.019	0.031	0.338
(Year 2019–2024)	−0.049	0.077	−0.634	107	0.528	−0.201	0.104

Note: *** *p* < 0.001 (highly significant).

**Table 10:** Meta-analysis of independent variables for dependent analysis.

Variable	k	*n*	β-Mean	Estimate	Standard Error	z	*p*	95% CI	
								Lower	Upper
**Behavioral Intention**									
Perceived Usefulness	63	26,429	0.32 ***	0.284	0.022	12.771	<0.001	0.241	0.328
Perceived Ease of Use	44	20,702	0.135 ***	0.136	0.025	5.369	<0.001	0.088	0.185
Social Influence	42	17,088	0.192 ***	0.177	0.026	8.758	<0.001	0.125	0.228
Perceived Risk	30	15,674	−0.0686	−0.059	0.029	−2.035	0.042	−0.116	−0.002
Facilitating Conditions	28	11,913	0.13 ***	0.153	0.031	4.954	<0.001	0.082	0.213
Perceived Enjoyment	21	9993	0.259 ***	0.222	0.036	6.234	<0.001	0.152	0.291
Self-efficacy	19	4932	0.272 ***	0.232	0.042	5.485	<0.001	0.149	0.315
Trust	15	6175	0.299 ***	0.267	0.048	5.965	<0.001	0.133	0.382
Perceived Value	14	5641	0.217 ***	0.164	0.043	3.837	<0.001	0.080	0.247
Attitude	12	6222	0.535 ***	0.400	0.055	7.337	<0.001	0.293	0.507
Creativity	11	5729	0.179 ***	0.199	0.048	4.170	<0.001	0.105	0.292
**Use Behavior**									
Behavioral Intention	22	11,077	0.532 ***	0.454	0.044	10.411	<0.001	0.369	0.540
Facilitating Conditions	10	3713	0.281 ***	0.217	0.059	3.702	<0.001	0.102	0.332
Social Influence	6	2179	0.329	0.237	0.099	2.395	0.017	0.043	0.430
Perceived Usefulness	5	2863	0.4798 ***	0.365	0.077	4.744	<0.001	0.214	0.516
Perceived Ease of Use	4	2813	0.313 **	0.234	0.083	2.806	0.005	0.071	0.397
Trust	4	1785	0.458 ***	0.477	0.097	4.938	<0.001	0.287	0.666
Attitude	4	1164	0.224	0.198	0.095	2.087	0.037	0.012	0.383
Creativity	4	1059	0.109	0.194	0.104	1.874	0.061	−0.009	0.397
**Attitude**									
Perceived Usefulness	17	8012	0.401 ***	0.307	0.041	7.574	<0.001	0.228	0.387
Perceived Ease of Use	13	6345	0.0963	0.067	0.039	1.736	0.083	−0.009	0.143
Perceived Risk	6	2308	−0.1605 **	−0.142	0.054	−2.608	0.009	−0.248	−0.035
Trust	5	2666	0.29 ***	0.275	0.070	3.908	<0.001	0.137	0.413
Perceived Enjoyment	5	2352	0.364 ***	0.276	0.076	3.650	<0.001	0.128	0.424
Social Influence	4	1687	0.251 **	0.206	0.068	3.045	0.002	0.073	0.338
Perceived Value	3	1126	0.207 ***	0.243	0.061	3.990	<0.001	0.123	0.362
Creativity	4	1271	0.2635	0.167	0.083	2.013	0.044	0.004	0.329
Facilitating Conditions	3	1058	0.219	0.218	0.085	2.561	0.01	0.051	0.385
**Acceptance**									
Attitude	7	3800	0.536 ***	0.512	0.103	4.989	<0.001	0.311	0.713
Perceived Ease of Use	7	2638	0.163	0.245	0.121	2.015	0.044	0.007	0.483
Perceived usefulness	6	2518	0.444 ***	0.449	0.123	3.645	<0.001	0.207	0.690
Social Influence	5	2149	0.293	0.236	0.119	1.983	0.047	0.003	0.470
Trust	4	1689	0.513 ***	0.484	0.120	4.027	<0.001	0.248	0.720
Facilitating Conditions	3	1194	0.125	0.041	0.153	0.265	0.791	−0.260	0.341
Perceived Risk	3	1905	−0.105	−0.154	0.259	−0.595	0.552	−0.661	0.353
Creativity	3	1335	0.118	0.118	0.237	0.498	0.618	−0.346	0.582
**Trust**									
Perceived Usefulness	6	2908	0.441 ***	0.574	0.087	6.568	<0.001	0.402	0.745
Perceived Enjoyment	3	1660	0.204	0.192	0.146	1.309	0.190	−0.095	0.479
Perceived Value	4	866	0.234 ***	0.321	0.097	3.318	<0.001	0.131	0.511
Social Influence	7	2255	0.218	0.234	0.098	2.380	0.017	0.041	0.427
Perceived Risk	11	3227	−0.131 **	−0.204	0.077	−2.655	0.008	−0.355	−0.053
Perceived Ease of Use	5	2412	0.187	0.176	0.113	1.557	0.119	−0.046	0.398
**Concern**									
Perceived Risk	3	981	0.504 ***	0.486	0.113	4.306	<0.001	0.265	0.708
Perceived Usefulness	5	1377	0.462 **	0.246	0.094	2.604	0.009	0.061	0.431
Social Influence	4	1383	0.039	0.062	0.087	0.716	0.474	−0.108	0.232
Perceived Ease of Use	3	814	0.062	0.069	0.096	0.716	0.474	−0.120	0.258
**Creativity**									
Self-efficacy	5	1388	0.4836 ***	0.480	0.114	4.226	<0.001	0.257	0.702
Behavioral Intention	3	1051	0.511 **	0.414	0.153	2.712	0.007	0.115	0.712

Note: Note: k: Total number of studies in the dataset involving altered quantities and number of effectors; *n*: refers to the number of study participants/users. *p*-value: likelihood of a chance event. Commonly used criteria are ** *p* < 0.01 (significant); *** *p* < 0.001 (highly significant). z-value: a statistic used to test whether the effect is significantly different from zero (i.e., whether it is a significant effect). The larger the z-value, the more likely it is to be significant. 95% CI: 95% Confidence Interval. The confidence interval refers to the likelihood that the true effect falls within the range of the 95% confidence level. If the interval does not contain 0, the effect is significant; if it contains 0, the effect may not be significant.

**Table 11:** Heterogeneity statistics of meta-analyses for key dependent variables.

Variable	Q	df	*p*	I^2^ (%)	95% CI	
					Lower	Upper
Behavioral Intention	2432	274	<0.001	92.936%	92.936	95.159
Use	299.109	52	<0.001	86.708%	82.708	94.05
Attitude	257.557	58	<0.001	81.65%	75.573	90.858
Acceptance	371.206	23	<0.001	92.236	85.910	96.502
Trust	284.223	36	<0.001	90.679	86.131	95.108
Concern	41.728	12	<0.001	81.298	61.3	97.138

Note: Cochran’s Q statistics. I^2^: to determine the magnitude of heterogeneity and the level of confidence.

**Table 12:** Total effects of the independent variables on BI in different subgroup models.

Domain	Variable	Estimate	Standard Error	z	*p*	95% CI	
						Lower	Upper
Education (k = 178, *n* = 26,294)	Perceived Usefulness	0.300 ***	0.028	10.860	<0.001	0.246	0.354
	Perceived playfulness	0.188 ***	0.041	4.554	<0.001	0.107	0.269
	Perceived Value	0.147	0.061	2.414	0.016	0.028	0.266
	Social Influence	0.169 ***	0.032	5.262	<0.001	0.106	0.232
	Perceived Risk	−0.054	0.032	−1.666	0.096	−0.118	0.010
	Facilitating Conditions	0.078	0.038	2.050	0.040	0.003	0.153
	Self-Efficacy	0.241 ***	0.046	5.257	<0.001	0.151	0.330
	Attitude	0.405 ***	0.068	6.100	<0.001	0.275	0.538
	Perceived Ease of Use	0.136 ***	0.027	4.952	<0.001	0.082	0.190
	Creativity	0.213 ***	0.056	3.806	<0.001	0.103	0.322
	Trust	0.237 ***	0.065	3.651	<0.001	0.110	0.365
Creative Industry (k = 39, *n* = 2937)	Perceived Usefulness	0.201 **	0.075	2.686	0.007	0.054	0.348
	Perceived Ease of Use	0.057	0.095	0.595	0.552	−0.130	0.243
	Perceived Value	0.178	0.126	1.416	0.157	−0.068	0.424
	Social Influence	0.142	0.078	1.832	0.067	−0.010	0.295
	Perceived Risk	−0.041	0.085	−0.474	0.635	−0.208	0.127
	Facilitating Conditions	0.387 ***	0.096	4.017	<0.001	0.198	0.576
	Self-Efficacy	0.184	0.123	1.488	0.137	−0.058	0.426
Healthcare (k = 12, *n* = 995)	Perceived Usefulness	0.335 **	0.112	2.996	0.003	0.116	0.555
	Perceived Risk	−0.031	0.087	−0.354	0.723	−0.202	0.140
	Social Influence	0.082	0.108	0.758	0.448	−0.129	0.292
Organization (k = 12, *n* = 1191)	Perceived Ease of Use	−0.022	0.134	−0.163	0.871	−0.285	0.241
	Perceived Usefulness	0.076 **	0.025	3.101	0.002	0.028	0.125
	Facilitating Conditions	0.101 **	0.038	2.684	0.007	0.027	0.175
Consumer Service Scenarios (k = 27, *n* = 3261)	Perceived Usefulness	0.289 ***	0.056	5.153	<0.001	0.179	0.398
	Trust	0.280 ***	0.083	3.394	<0.001	0.118	0.442
	Perceived Ease of Use	0.351 ***	0.089	3.954	<0.001	0.177	0.526
	Social Influence	0.334 ***	0.074	4.531	<0.001	0.189	0.478
	Perceived playfulness	0.359 ***	0.074	4.829	<0.001	0.213	0.504
	Attitude	0.309 ***	0.070	4.436	<0.001	0.173	0.446
	Self-Efficacy	0.323	0.179	1.809	0.070	−0.027	0.673
General-Purpose Scenarios (k = 21, *n* = 3718)	Perceived Usefulness	0.344 ***	0.072	4.815	<0.001	0.204	0.484
	Perceived Ease of Use	0.206 **	0.076	2.722	0.006	0.058	0.354
	Social Influence	0.240 **	0.091	2.646	0.008	0.062	0.418
	Facilitating Conditions	0.150	0.090	1.663	0.096	−0.027	0.327
	Perceived Value	0.169	0.098	1.718	0.086	−0.024	0.361
	Perceived Risk	−0.600 **	0.208	−2.879	0.004	−1.008	−0.192

Note: k: Total number of studies in the dataset involving altered quantities and number of effectors; *n*: refers to the number of study participants/users. ** *p* < 0.01 (significant); *** *p* < 0.001 (highly significant).

**Table 13:** Independent variables (positive and negative) and moderating variables in education.

Education (*n* = 93)		
Positive Factor	Negative Factor	Regulatory Factor
Perceived confirmation (Tian et al. 2024), cost ethical awareness (Zhu et al. 2024), usefulness, social presence, legitimacy of the tool, enjoyment, motivation (Tiwari et al. 2023), knowledge sharing (Duong et al. 2023), design, interactivity, perceived trust (Salifu et al. 2024), functional elements (Ibrahim et al. 2023), emotional intelligence (Zhou and Zhang 2024), anthropomorphism, design novelty, trust (Polyportis and Pahos 2024), feedback and quality, subject norms (Almogren et al. 2024), perceived value (Chan and Zhou 2023), information system (Thongsri et al. 2024), subjective norms, perceived behavioral control (Al-Qaysi et al. 2024), perceived use contexts (Y. Chang et al. 2022), autonomy (Niu et al. 2024), course level, motivation, confidence (Amoozadeh et al. 2024), potential of GenAI enjoyment (Liu et al. 2024), content quality, emotional wellbeing, perceived utility (Almufarreh 2024), personal ability, perceived intelligence, perceived enjoyment (Dahri et al. 2024), personal innovativeness (Hernandez et al. 2023; Nawaz et al. 2024)	Perceived cost ethical awareness, perceived ethical risk, artificial intelligence ethical anxiety (W. Li 2024; Zhu et al. 2024), psychological risk (Wu et al. 2022), perceived cost (Chan and Zhou 2023), potential risks (L. Law 2024), fake information (Amaro et al. 2024), over-reliance, accuracy, ethical considerations (Abu Hammour et al. 2023), impeding learning, producing inaccurate information (Anderson et al. 2024)	Institutional policy (Polyportis and Pahos 2024), gender, age, experience, voluntariness of use, teachers’ teaching level and experience, students’ major (X. Lin et al. 2024), academic disciplines, geographical locations, cultural orientations (Yusuf et al. 2024), SDT motivation (Y. Wang et al. 2024), educational backgrounds (Yao and Abd Halim 2024), AI training courses (Pellas 2023), task–technology fit (Du and Lv 2024)

Note: *n*: number of study.

**Table 14:** Independent variables (positive and negative) and moderating variables in creative arts.

Creative Arts and Design (*n* = 28)		
Positive Factor	Negative Factor	Regulatory Factor
Optimism, creativity, trait curiosity (indirect) (Y. Wang and Zhang 2023), confirmation, satisfaction ,personal innovativeness (Yu et al. 2024), design priorities, AI literacy (T. Li et al. 2023), perceived relevance, autonomy, competence (Latikka et al. 2023), auxiliary action (Saadi and Yang 2023), perceptual intelligence, personify, individuation (Zhou and Zhang 2024), AI image filter (Papadopoulou et al. 2024), perceived Intelligence (C. Gu et al. 2024), satisfaction, perceived usefulness, self-efficacy, technology trust (H. Ma and Li 2024), social system, individual innovativeness, communication channels, AI anxiety, relative advantage, performance expectancy, effort expectancy, facilitating conditions (Qiu et al. 2024)	AI anxiety (anxiety about GenAI learning (AL), anxiety about job substitution (JP), and anxiety about socio-technical blindness (SB) in the use of GenAI (Yin et al. 2023), privacy concerns information illusion (Zhou and Zhang 2024), copyright issue (C. Wang 2024), the label of an AI artwork (made by AI or made by humans), the perception of creativity, the sense of awe (Kokkoris et al. 2023), perceived eeriness (C. Gu et al. 2024), social influence	Habit (Y. Wang and Zhang 2023), educational level (Yin et al. 2023), functional factor, art background, art experience (Lyu et al. 2022), gender, design art level, education level (C. Wang 2024)

Note: *n*: number of study.

**Table 15:** Independent variables (positive and negative) and moderating variables in healthcare.

Healthcare (*n* = 24)		
Positive Factor	Negative Factor	Regulatory Factor
Information credibility, perceived application value and reliability, decision-making (Stevens and Stetson 2023), depression levels, perceived usefulness, and parasocial interactions (Park and Kim 2023), performance expectancy, price value, descriptive norm, psychological distress, the potential to increase accuracy, speed, and efficiency in medical decision making (Amann et al. 2023), transparency, autonomy (Haber et al. 2024), trust, information, credibility, system performance, application value (Shahsavar and Choudhury 2023), AI smartness, AI transparency (Hou et al. 2024), initial trust, performance expectation, effort expectations, trust tendency, social influence (X. Wang and Wang 2024)	Images inaccuracies (Ozmen and Schwarz 2024), AI hesitancy and effort expectancy, privacy and security issues, questions of accuracy and authenticity, ethical and legal issues, lack of control (Xu and Wang 2024), generated hype (Gravina et al. 2024)	Personal innovation, task complexity (X. Wang and Wang 2024)

Note: *n*: number of study.

**Table 16:** Independent variables (positive and negative) and moderating variables in commercial organizations.

Organization (*n* = 20)		
Positive Factor	Negative Factor	Regulatory Factor
Performance expectancy, perceived usefulness (Herani and Angela 2024), facilitating conditions, hedonic motivation, performance expectancy (Maican et al. 2023), agile leadership, innovation orientation, agile leadership (Cimino et al. 2024), perceived usefulness, perceived ease of use, perceived enjoyment, anthropomorphism (V. Gupta 2024), compatibility, organizational size, competition intensity, perceived ease of use, trust, facilitating conditions, perceived value, perceived autonomy, perceived usefulness (Tanantong et al. 2024), AI engagement, AI familiarity (De Vreede et al. 2024), coercive pressure, normative pressure, mimetic pressure, fairness, accountability, transparency, accuracy, autonomy (Rana et al. 2024), user experience (Marimon et al. 2024), functional value, social value, emotional value, epistemic value, information control (Huynh 2024)	Effort expectancy, social influence, perceived customer value (PCV) (Maican et al. 2023), information sensitivity (Huynh 2024), regulatory support, complexity (Prasad Agrawal 2024)	Interaction convenience, system quality, training and support, technology experience, domain experience (V. Gupta 2024), public knowledge, private knowledge (Figueiredo et al. 2022)

Note: *n*: number of study.

**Table 17:** Top 10 authors of relevant publications on the impact of user attitudes towards GenAI.

Rating	Author	h_Index	g_Index	TC	NP	PY_Start
1	Gursory D	5	5	817	5	2019
2	Kim J	3	4	16	4	2023
3	Park J	3	3	12	4	2023
4	AL-Emran M	2	2	8	2	2023
5	Baek TH	2	2	39	2	2023
6	Balakrishnan J	2	2	795	2	2022
7	Chi OH	2	2	131	2	2022
8	Chi OHX	2	2	625	2	2019
9	Chiu TKF	2	2	55	2	2023
10	Choudhury S	2	2	809	2	2023

Note: Data comes from WoSCC. NP: number of publications; TC: total citations; H-index: Hirsch index, an author-level metric used to measure authors’ scholarly influence; g_index: another measure of scholarly influence; PY-start: year of the first publication.

**Table 18:** The top ten institutions with the highest number of publications.

Count	Central	Year	Institution	Country
11	0.27	2021	State University System of Florida	USA
7	0.14	2020	University of London	UK
6	0	2024	University of California System	USA
6	0.16	2019	University of Johannesburg	South Africa
5	0.01	2023	Chinese University of Hong Kong	China
5	0	2019	Washington State University	USA
5	0	2024	University of Pennsylvania	USA
4	0.01	2024	University of Houston	USA

**Table 19:** Future research trends.

Research Focus	Current Gaps	Potential Issues	Future Research Directions
Theoretical Model Development	Traditional models do not fit GenAI	Neglect of emotional motivations and individual differences	Cross-level integration, diverse variable systems
Sample Heterogeneity Analysis	High I^2^ heterogeneity across studies	Weak predictive power; unclear mechanisms	Group-specific path analysis and inclusion of moderators
Multimodal Acceptance Mechanisms	Overemphasis on text-based tools	Ignorance of modality-specific perceptual differences	Cross-modal comparisons
Cross-Cultural Research	Lack of cultural diversity in samples	Variable effects differ across cultures	International collaboration
Human-AI Co-Creation	Blurred roles, reduced cognitive agency	Creativity erosion	Mechanism for co-creation, cognitive boundary research

## Supplementary Materials

The following supporting information can be downloaded at https://www.mdpi.com/article/10.3390/jintelligence13070078/s1. Table S1: Study included in meta-analysis of effect size (independent variable to dependent variable).

## Appendix A

**Table A1:** Independent variables (positive and negative) and moderating variables of another field.

Field	Positive Factor	Negative Factor	Regulatory Factor
Travel and Hotel Services (*n* = 14)	Practicality (T. Kim et al. 2024), perceived trust (R. Law et al. 2024), satisfaction, parasocial interaction (Duong et al. 2024), perceived coolness, perceived enjoyment, perceived usefulness, perceived ease of use (S. Li et al. 2024), anthropomorphism, expectancy, emotion (Rao et al. 2020; H. Lin et al. 2020), communication quality, accuracy, timeliness, understandability, personalization (Y. Li and Lee 2024), emotions, performance expectancy (Vitezić and Perić 2021), advice elaboration (Spatscheck et al. 2024)	Incorrect information (J. H. Kim et al. 2025), effort expectancy (Vitezić and Perić 2021), anthropomorphism (Spatscheck et al. 2024)	Individual innovation (T. Kim et al. 2024), AI hallucination (Christensen et al. 2024), technology anxiety (Duong et al. 2024), ethical and quality concerns (J. H. Kim et al. 2023)
Academic research (*n* = 13)	Time and energy saving, innovative software (Durak and Cankaya 2024), capability to simulate human behavior, enhancement in research techniques (Bail 2024), awareness of GAI advantages (Kshetri 2024), writing motivation, self-efficacy, engagement, collaborative writing tendency (Teng 2024), personal innovativeness (Strzelecki et al. 2024)	Negative effects on reading habits, reduced originality, academic dishonesty, and monotonous writing (Durak and Cankaya 2024) Bias in AI Tools, risk of replication, poor quality studies, misinformation, ethical (Bail 2024), menace to original thinking, new scientific ideas, academic, research integrity (Bannister et al. 2023; Kapsali et al. 2024),	Familiarity and experience with technology, academic background, institutional policies and guidelines, cultural and educational values (Durak and Cankaya 2024), open-source infrastructure, community standards, and guidelines (Bail 2024)
Shopping (*n* = 10)	Perceived interaction quality, perceived trustworthiness, perceptual anthropomorphism (Chakraborty et al. 2024), competence, warmth, empathy (Woo and Hur 2023), utilitarian factors, hedonic factors (Marjerison et al. 2022), enjoyment, subjective norms (Sohn and Kwon 2020)	Perceived inertia, perceived threats, perceived regret avoidance (Chakraborty et al. 2024), privacy concerns, technology immaturity (Marjerison et al. 2022)	Anthropomorphism (Chakraborty et al. 2024), visual, textual closeness GenAI experience level (Martínez Puertas et al. 2024), brand awareness (W. Chang and Park 2024)
Software Engineering (*n* = 2)	Compatibility, social factors. perceptions about the technology (Russo 2024), perceived Intelligence, perceived Anthropomorphism (Diirr et al. 2024)		Interaction Intensity (Diirr et al. 2024)
Catering Service (*n* = 2)	Perceived risk (K. P. Gupta and Pande 2022)		Gender, familiarity, experience (K. P. Gupta and Pande 2022)

Note: *n*: number of study.
