# Supplementary material for: A Systematic Review of User Attitudes Toward GenAI: Influencing Factors and Industry Perspectives
Source: J Intell. 2025 Jun 27;13(7):78. doi: 10.3390/jintelligence13070078 (PMC12295719; doi:10.3390/jintelligence13070078)
Supplement: Supplementary file 1 [file jintelligence-13-00078-s001.zip › jintelligence-3435784-Supplementary.pdf]

Supplementary Materials of the Manuscript: Sex Differences on Cognitive Reflection: A meta-analysis

Junjie Chen [1,2], Wei Xie [1,2], Qing Xie [3], Anshu Hu [3], Yiran Qiao [3], Ruoyu Wan [1] and Yuhan Liu [1,2,4,*]

[1] School of Design, Huazhong University of Science and Technology, Wuhan 430074, China
[2] MoCT Key Laboratory of Lighting Interactive Service & Tech, Huazhong University of Science and Technology, Wuhan 430074, China
[3] School of Computer Science and Artificial Intelligence, Wuhan University of Technology, Wuhan 430070, China
[4] Cognitive Aesthetics Media Lab (CAMLab), Harvard Faculty of Arts and Sciences (FAS), Harvard University, Cambridge, MA 02138, USA

Table S1.Study included in meta-analysis of effect size (independent variable to dependent variable)

Study	Theories used	Domain	DV(Dependent Variable）	IV Independent Variable	β	N	p
(Zhu et al. 2024)	Extended UTAUT2	Education	Use Behavior(UB)	Facilitating Conditions(FC)	0.231	226	<0.01
			Use Behavior(UB)	Habit	0.356	226	<0.001
			Use Behavior(UB)	Perceived risk	-0.152	226	<0.05
			Behavioral Intention(BI）	Performance Expectancy(PE)	0.317	226	<0.001
			Behavioral Intention(BI）	Hedonic Motivation(HM)	0.181	226	<0.05
			Behavioral Intention(BI）	Price Value(PV)	0.144	226	<0.01
			Behavioral Intention(BI）	Social Influence (SI）	0.217	226	<0.01
			Anxiety	Perceived risk	0.584	226	<0.001
			Concern	Ethical Awareness(EA)	0.488	226	<0.001
(Amoozadeh et al. 2024)	Self-built framework	Education	Trust	Gender	0.24	253	<0.05
			Trust	Age	-0.18	253	<0.05
			Trust	Performance Expectancy(PE)	0.317	253	<0.001
			Trust	Hedonic Motivation(HM)	0.181	253	<0.05
			Trust	Price Value(PV)	0.144	253	<0.01
			Trust	Social Influence（SI）	0.217	253	<0.01
			Trust	Habit	0.356	253	<0.001
			Trust	AI Learning Anxiety	0.14	253	<0.001
			Trust	AI Configuration Anxiety	-0.19	253	<0.001
(Chan and Zhou 2023)	EVT	Education	Behavioral Intention(BI）	Perceived Value(PV)	0.606	405	<0.001

			Intentions to learn AI	Perceived cost	-0.295	405	<0.001
(F. Wang et al. 2023)	EVT	Education	Intentions to learn AI	Supportive social norms	0.08	494	<0.001
			Intentions to learn AI	Facilitating Conditions(FC)	0.14	494	<0.001
			Intentions to learn AI	Self-Efficacy	0.23	494	<0.001
			Intentions to learn AI	Perceived Usefulness (PU)	0.3	494	<0.001
(Tiwari et al. 2023)	TAM	Education	Attitude	Perceived Usefulness (PU)	0.311	375	<0.001
			Attitude	Perceived Credibility	0.328	375	<0.001
			Attitude	Perceived Social Presence	0.247	375	0.005
			Attitude	Hedonic Motivation(HM)	0.391	375	<0.001
			Intention to Use ChatGPT(IUCG)	Attitude	0.649	375	<0.001
(Hidayat-ur-Rehman and Ibrahim 2023)	UTAUT and SQB	Education	Intention to Use ChatGPT(IUCG)	Effort Expectancy(EE)	0.187	243	<0.002
			Intention to Use ChatGPT(IUCG)	Educators Autonomous Motivation (EAM)	0.088	243	<0.039
			Intention to Use ChatGPT(IUCG)	Innovative Behaviour towards Technological Agility (IBTTA)	0.214	243	<0.000
			Intention to Use ChatGPT(IUCG)	Perceived Students Engagement (PSE)	0.198		<0.000
			Intention to Use ChatGPT(IUCG)	Cognitive Need (CN)	0.17	243	<0.000
			Intention to Use ChatGPT(IUCG)	Information Quality (INQ)	0.155	243	<0.007
			Intention to Use ChatGPT(IUCG)	System Quality (SYQ)	-0.218	243	<0.000
(Sudan et al. 2024)	UTAUT2	Education	Behavioral Intention	Service Quality (SEQ)	0.46	638	0.000*
			Behavioral Intention	Personal Innovativeness (PI)	0.263	638	0.000*
			Behavioral Intention	PI × BEH (Personal Innovativeness × Behavioural Intention)	0.088	638	0.126
			Behavioral Intention	Reasons for Adoption (RFA)	0.041	638	0.848
			Behavioral Intention	Social Influence（SI）	0.022	638	0.934
			Behavioral Intention	Hedonic Motivation(HM)	0.11	638	0.124

(Thongsri et al. 2024)	UTG	Educ ation	Behavioral Intention	Affective Need	0.087	679	0.012
			Behavioral Intention	Cognitive Need (CN)	0.189	679	<0.001
			Behavioral Intention	Information Quality (INQ)	0.049	679	0.001
			Behavioral Intention	System Quality (SYQ)	0.055	679	<0.001
			Behavioral Intention	Service Quality (SEQ)	0.567	679	<0.001
			Behavioral Intention	Personal Innovativeness (PI)	0.066	679	0.358
(W. Li 2024)	UTAUT	Educ ation	Behavioral Intention	Perceived Usefulness	0.567	404	<0.001
			Behavioral Intention	Social Influence	0.241	404	<0.001
			Behavioral Intention	Perceived Risk	-0.17	404	<0.001
			Behavioral Intention	Perceived Ease of Use	0.056	404	0.357
			Behavioral Intention	Facilitating Conditions	-0.044	404	0.068
			Behavioral Intention	Perceived Risk	0.042	404	0.097
(Nawaz et al. 2024)	UTAUT2	Educ ation	Behavioral Intention	Performance Expectancy(PE)	0.295	500	<0.001
			Behavioral Intention	Perceived Ease of Use (PEU)	0.266	500	<0.001
			Behavioral Intention	Social Influence（SI）	0.117	500	<0.001
			Behavioral Intention	Facilitating Conditions(FC)	0.212	500	<0.001
			Behavioral Intention	Hedonic Motivation(HM)	0.235	500	<0.001
			Behavioral Intention	Habit	0.272	500	<0.001
			Behavioral Intention	Personal Innovativeness (PI)	0.383	500	<0.001
			Use Behavior(UB)	Behavioural Intention	0.382	500	<0.001
(Al-Qaysi et al. 2024)	TPB and BRT	Educ ation	Use Behavior(UB)	Attitude	0.394	357	<0.001
			Use Behavior(UB)	Subjective Norms(SN)	0.213	357	0.003
			Use Behavior(UB)	Perceived Behavioral Control(PBC)	0.297	357	<0.001
			Attitude	Reasons for Adoption	0.529	357	<0.001
			Attitude	Reasons against Adoption	-0.292	357	<0.001
(Amaro et al. 2024)	Multiple	Educ ation	Trust	Fake Information	-0.48	62	0.0077

			Trust	Early Fake	-0.64	62	<0.05
			Trust	Order of Fake Information	-0.64	62	0.0066
(Ngo et al. 2024)	TAM	Educ ation	Satisfaction	Expectation Confirmation	0.515	513	<0.001
			Satisfaction	Knowledge Acquisition	0.192	513	<0.001
			Satisfaction	Knowledge Sharing	0.087	513	<0.05
			Satisfaction	Knowledge Sharing	0.077	513	<0.05
			Satisfaction	Knowledge Application	0.088	513	<0.05
			Continuance Usage Intention	Student Satisfaction	0.255	513	<0.001
			Continuance Usage Intention	Student Satisfaction	0.315	513	<0.001
			Continuance Usage Intention	Knowledge Acquisition	0.184	513	<0.001
			Continuance Usage Intention	Knowledge Sharing	0.057	513	0.153
			Continuance Usage Intention	Knowledge Application	0.076	513	0.124
(Yusuf et al. 2024)	TAM	Educ ation	Behavioral Intention	Perceived Usefulness (PU)	0.96	867	<0.001
			Behavioral Intention	Perceived Ease of Use (PEU)	0.05	867	0.48
			Use Behavior(UB)	Behavioural Intention	0.85	867	<0.001
(Almufarreh 2024)	ANN	Educ ation	Satisfaction	Content Quality	0.488	355	0
			Satisfaction	Emotional Wellbeing	0.478	355	0
			Satisfaction	Performance Expectancy(PE)	0.260	355	0
			Satisfaction	Cognitive Absorption	0.092	355	0.128
			Satisfaction	Perceived Credibility	0.083	355	0.137
(Y. Wang et al. 2024)	UTAUT2 and SDT	Educ ation	Use Behavior(UB)	Behavioural Intention	0.47	620	<0.001
			Use Behavior(UB)	Habit	0.148	620	<0.001
			Use Behavior(UB)	SDT Motivation	0.293	620	<0.001
			Use Behavior(UB)	Facilitating Conditions(FC)	0.141	620	>0.05
			Behavioral Intention	Performance Expectancy(PE)	0.191	620	<0.001
			Behavioral Intention	Effort Expectancy(EE)	0.177	620	<0.001
			Behavioral Intention	Social Influence（SI）	0.175	620	<0.001
			Behavioral Intention	Hedonic Motivation(HM)	0.166	620	<0.001

			Behavioral Intention	Habit	0.156	620	<0.001
			Behavioral Intention	Price Value(PV)	0.025	620	0.542
			Behavioral Intention	SDT Motivation	0.14	620	<0.001
			Behavioral Intention	Facilitating Conditions(FC)	0.144	620	<0.001
(Hernandez et al. 2023)	UTAUT2	Education	Use Behavior(UB)	Facilitating Conditions(FC)	0.102	520	0.013
			Use Behavior(UB)	Habit	0.318	520	0
			Use Behavior(UB)	Behavioural Intention	0.416	520	0
			Behavioral Intention	Habit	0.476	520	0
			Behavioral Intention	Performance Expectancy(PE)	0.217	520	0
			Behavioral Intention	Personal Innovativeness	0.122	520	0.005
(Duong et al. 2023)	TAM	Education	Use Behavior(UB)	Effort Expectancy(EE)	0.166	1389	<0.001
			Use Behavior(UB)	Behavioural Intention	0.462	1389	<0.001
			Use Behavior(UB)	Performance Expectancy(PE)	0.123	1389	<0.001
			Behavioral Intention	Effort Expectancy(EE)	0.457	1389	<0.001
			Behavioral Intention	Performance Expectancy(PE)	0.528	1389	<0.001
(Raman et al. 2024)	Multiple	Education	Intention to Use ChatGPT	Relative Advantage	0.228	288	0.001
			Intention to Use ChatGPT	Compatibility	0.187	288	0.005
			Intention to Use ChatGPT	Perceived Ease of Use (PEU)	0.436	288	0.001
			Intention to Use ChatGPT	Trialability	0.171	288	0.01
			Intention to Use ChatGPT	Observability	0.325	288	0.014
(Shen et al. 2024)	TAM and SDT	Education	Behavioral Intention	Original Privacy Concerns	-0.159	308	<0.05
			Behavioral Intention	Job Replacement Anxiety(JRA）	-0.277	308	<0.01
			Behavioral Intention	Perceived Usefulness (PU)	0.854	308	<0.001
			Behavioral Intention	Perceived Ease of Use (PEU)	0.029	308	>0.05
			Anxiety	Perceived Usefulness (PU)	0.956	308	<0.001
			Anxiety	Perceived Relevance (PR)	-0.54	308	<0.001
			Anxiety	Perceived	-0.202	308	>0.05

				Relevance			
			Concern	Perceived Usefulness (PU)	0.925	308	<0.001
			Concern	Perceived Relevance (PR)	-0.552	308	<0.001
			Concern	Perceived Relevance	-0.375	308	<0.01
(Salifu et al. 2024)	TAM, Human-Computer Interaction theory, Algorithmic Trust Theory and Information Processing Theory	Education	Trust	Design	0.238	306	<0.001
			Trust	Interactivity	0.482	306	<0.001
			Trust	Ethics	0.036	306	>0.05
			Behavioral Intention	Effort Expectancy(EE)	0.058	306	>0.05
			Behavioral Intention	Performance Expectancy(PE)	0.116	306	<0.05
			Behavioral Intention	Social Influence（SI）	0.097	306	<0.05
			Behavioral Intention	Hedonic Motivation(HM)	0.336	306	<0.001
			Behavioral Intention	Habit	0.143	306	<0.01
			Use Behavior(UB)	Behavioural Intention	0.557	306	<0.001
			Use Behavior(UB)	Facilitating Conditions(FC)	0.383	306	<0.001
(Ivanov et al. 2024)	TPB	Education	Attitude	Strengths of GenAI	0.251	298	<0.05
			Attitude	Benefits of GenAI	0.32	298	<0.05
			Behavioral Intention	Attitude	0.45	298	<0.05
			Behavioral Intention	Subjective Norms(SN)	0.38	298	<0.05
			Behavioral Intention	Perceived Behavioral Control(PBC)	0.35	298	<0.05
			Use Behavior(UB)	Behavioural Intention	0.52	298	<0.05
(Sun et al. 2024)	NA	Education	Attitude	Interactive AI Image Processing	-0.37	96	<0.05
			Continuous Learning Intention	Interactive AI Image Processing	-0.45	96	<0.05
(Pellas 2023)	NA	Education	Attitude	Number of AI Training Courses Participated	-0.043	398	0.004
			Attitude	Gender	-0.041	398	0.069

			Attitude	Age	0.028	398	0.055
			Attitude	General ICT Usage	-0.1	398	0.556
			Attitude	Appropriateness of AI-Generated Video Content Creation	-0.19	398	0.426
			Attitude	Number of Devices for AI Content Access and Creation	-0.012	398	0.142
			Attitude	Frequency of AI Platform Usage	-0.008	398	0.683
			Attitude	Tech-savviness	-0.018	398	0.257
(Tian et al. 2024)	UTAUT and ECM	Educ ation	Behavioral Intention	Performance Expectancy(PE)	0.203	373	0.004
			Behavioral Intention	Effort Expectancy(EE)	-0.057	373	0.35
			Behavioral Intention	Social Influence（SI）	0.059	373	0.382
			Behavioral Intention	Facilitating Conditions(FC)	0.051	373	0.364
			Behavioral Intention	Satisfaction	0.059	373	0
			Behavioral Intention	Personal Innovativeness	0.258	373	0
			Use Behavior(UB)	Facilitating Conditions(FC)	0.109	373	0.051
			Use Behavior(UB)	Behavioural Intention	0.401	373	0
			Satisfaction	Performance Expectancy(PE)	0.419	373	0
			Satisfaction	Effort Expectancy(EE)	0.285	373	0
			Satisfaction	Confirmation	0.194	373	0
(Yildiz et al. 2024)	UTAUT2	Educ ation	Behavioral Intent to Use/Learn Chatbot (BIULC)	Performance Expectancy(PE)	0.178	926	0.008
			Behavioral Intent to Use/Learn Chatbot (BIULC)	Effort Expectancy(EE)	-0.17	926	0.012
			Behavioral Intent to Use/Learn Chatbot (BIULC)	Attitude	0.218	926	0
			Behavioral Intent to Use/Learn Chatbot (BIULC)	Self-Efficacy	0.324	926	0
			Behavioral Intent to Use/Learn Chatbot (BIULC)	Perceived Anxiety	0.073	926	0
			Behavioral Intent to Use/Learn Chatbot (BIULC)	Sustainability of Use	0.232	926	0
			Behavioral Intent	Facilitating	0.083	926	0.111

			to Use/Learn Chatbot (BIULC)	Conditions(FC)			
			Behavioral Intent to Use/Learn Chatbot (BIULC)	Social Influence（SI）	-0.003	926	0.948
(Yao and Abd Halim 2024)	UTAUT	Education	Acceptance	Performance Expectancy(PE)	0.598	428	<0.001
			Acceptance	Social Influence（SI）	0.138	428	<0.1
			Acceptance	Cognitive Factors	0.027	428	>0.05
			Acceptance	Effort Expectancy(EE)	0.018	428	>0.05
			Acceptance	Facilitating Conditions(FC)	-0.112	428	>0.05
(Y. Chang et al. 2022)	UGT and Belief-Attitude-Intention Theory	Education	Continuance Usage Intention	Attitude	0.762	478	<0.001
			Attitude	Cognitive	0.279	478	<0.001
			Attitude	Affective	0.342	478	<0.001
			Attitude	Personal Integrative	0.079	478	<0.05
			Attitude	Social Integrative	-0.072	478	>0.05
			Attitude	Interaction with AI	0.2	478	<0.001
			Attitude	Customization	0.225	478	<0.001
(Niu et al. 2024)	UGT	Education	Behavioral Intention	Sensing Autonomy	0.049	673	0.302
			Behavioral Intention	Thought Autonomy	0.047	673	0.231
			Behavioral Intention	Action Autonomy	0.03	673	0.533
			Satisfaction	Sensing Autonomy	-0.075	673	0.143
			Satisfaction	Sensing Autonomy	0.315	673	<0.001
			Satisfaction	Sensing Autonomy	0.383	673	<0.001
			Satisfaction	Thought Autonomy	0.215	673	<0.001
			Satisfaction	Thought Autonomy	0.238	673	<0.001
			Satisfaction	Action Autonomy	0.374	673	<0.001
			Satisfaction	Action Autonomy	0.306	673	<0.001
(Bhat et al. 2024)	Extended UTAUT	Education	Use Behavior(UB)	Behavioural Intention	0.34	1214	<0.01
			Use Behavior(UB)	Trust	0.25	1214	>0.05
			Behavioral Intention	Performance Expectancy(PE)	0.29	1214	<0.05
			Behavioral Intention	Effort Expectancy(EE)	0.28	1214	<0.05
			Behavioral Intention	Social Influence（SI）	0.15	1214	>0.05
			Behavioral Intention	Facilitating Conditions(FC)	0.36	1214	<0.05
			Behavioral Intention	Hedonic Motivation(HM)	0.47	1214	<0.001
			Behavioral Intention	Habit	0.37	1214	<0.001

			Behavioral Intention	Personal Innovation	0.2	1214	>0.05
(Sobaih et al. 2024)	UTAUT2	Education	Behavioral Intention	Performance Expectancy(PE)	0.398	520	<0.001
			Behavioral Intention	Effort Expectancy(EE)	0.144	520	0.009
			Behavioral Intention	Social Influence（SI）	0.445	520	<0.001
			Behavioral Intention	Facilitating Conditions(FC)	-0.204	520	<0.001
			Use Behavior(UB)	Performance Expectancy(PE)	0.141	520	<0.001
			Use Behavior(UB)	Social Influence（SI）	0.07	520	0.009
			Use Behavior(UB)	Behavioural Intention	0.789	520	<0.001
(Wu et al. 2022)	UTAUT and perceived risk theory	Education	Use Behavior(UB)	Emotional Intelligence EI	0.925	524	<0.001
			Use Behavior(UB)	Performance Expectancy(PE)	0.926	524	<0.001
			Use Behavior(UB)	Effort Expectancy	0.823	524	<0.001
			Use Behavior(UB)	Social Influence（SI）	0.926	524	<0.001
			Use Behavior(UB)	Facilitating Conditions(FC)	0.925	524	<0.001
(Du and Lv 2024)	Extended UTAUT and TTF	Education	Behavioral Intention	Performance Expectancy(PE)	0.25	279	<0.05
			Behavioral Intention	Social Influence（SI）	0.213	279	<0.05
			Behavioral Intention	Effort Expectancy(EE)	0.189	279	<0.05
			Use Behavior(UB)	Facilitating Conditions(FC)	0.385	279	<0.001
			Use Behavior(UB)	Behavioural Intention	0.283	279	<0.05
(Yilmaz et al. 2023)	UTAUT	Education	Acceptance	Performance Expectancy(PE)	0.72	338	<0.01
			Acceptance	Effort Expectancy	0.65	338	<0.01
			Acceptance	Social Influence（SI）	0.75	338	<0.01
			Acceptance	Facilitating Conditions(FC)	0.6	338	<0.01
(Bilquise et al. 2023)	TAM,UTAUT,AI-driven self-service technology model ,sRAM and SDT	Education	Acceptance	Perceived Usefulness (PU)	0.15	300	<0.01
			Behavioral Intention	Perceived risk	0.25	450	<0.001
			Behavioral	Trust	0.2	400	<0.05

			Intention				
(K. Wang et al. 2024)	UTAU and TPACK Framework	Education	Acceptance	Social Influence（SI）	0.3	350	<0.001
			Behavioral Intention	Effort Expectancy(EE)	0.047	606	0.298
			Behavioral Intention	Facilitating Conditions(FC)	0.071	606	0.136
			Behavioral Intention	Social Influence（SI）	0.346	606	<0.001
			Behavioral Intention	Performance Expectancy(PE)	0.442	606	<0.001
			Behavioral Intention	Perceived Anxiety	-0.166	606	<0.001
			Anxiety	Self-Efficacy	-0.466	606	<0.001
(Fu et al. 2024)	UTAUT and PMT	Education	Behavioral Intention	Perceived Vulnerability	-0.15	445	<0.001
			Behavioral Intention	Perceived Severity	-0.034	445	ns
			Behavioral Intention	Response Efficacy	0.128	445	<0.05
			Behavioral Intention	Response Cost	0.018	445	ns
			Behavioral Intention	Self-Efficacy	0.114	445	<0.01
			Behavioral Intention	Task Efficiency	0.252	445	<0.001
			Behavioral Intention	Hedonic Motivation(HM)	0.121	445	<0.05
			Behavioral Intention	Performance Expectancy(PE)	0.3	445	<0.001
			Behavioral Intention	Effort Expectancy	0.076	445	<0.01
			Use Behavior(UB)	Behavioural Intention	0.236	445	<0.001
(Bouteraa et al. 2024)	Extended Meta-UTAUT	Education	Use Behavior(UB)	Behavioural Intention	0.236	445	<0.001
			Use Behavior(UB)	Behavioural Intention	0.935	355	<0.01
			Use Behavior(UB)	Attitude	0.34	355	<0.01
			Behavioral Intention	Performance Expectancy(PE)	0.197	355	<0.01
			Behavioral Intention	Social Influence（SI）	0.221	355	<0.01
			Behavioral Intention	Facilitating Conditions(FC)	0.114	355	<0.05
			Behavioral Intention	Attitude	0.595	355	<0.01
			Attitude	Performance Expectancy(PE)	0.58	355	<0.01
			Attitude	Effort	0.139	355	<0.01

				Expectancy(EE)			
			Attitude	Anthropomorphism	0.203	355	<0.01
			Attitude	Design Novelty	0.144	355	<0.01
			Attitude	Trust	0.109	355	<0.05
(Dahri et al. 2024)	Extended TAM	Education	Behavioral Intention	Personal Competence	0.16	300	<0.001
			Behavioral Intention	Social Influence（SI）	0.21	300	<0.001
			Behavioral Intention	Trust	0.12	300	0.03
			Behavioral Intention	Perceived Usefulness (PU)	0.34	300	<0.001
			Behavioral Intention	Perceived AI Enjoyment	0.12	300	0.03
			Behavioral Intention	Perceived Intelligence	0.12	300	0.21
			Behavioral Intention	Metacognitive Self-regulated Learning	0.36		<0.001
(Rajan and Niranjan 2025)	UTAUT and TRA	Education	Creativity	Usage behavior	0.053	403	>0.05
			Creativity	Intrinsic Motivation	0.692	403	<0.001
(Pellas 2025)	Higher-order thinking skills theory	Education	Attitude	Academic Achievements	0.39	416	<0.001
			Attitude	Creativity	0.33	416	<0.001
			Attitude	Critical Thinking	-0.1	416	<0.05
			Attitude	Problem-solving	-0.09		<0.05
			Creativity	Academic Achievements	0.33	416	<0.001
(M. F. Shahzad et al. 2024)	SCT	Education	Creativity	Generative AI-based Technologies, GAIT	0.361	362	p<0.001
(Avci 2024)	Extended TAM	Education	Acceptance	Age	0.028	183	0.994
			Acceptance	Gender	0.219	183	0.003
			Acceptance	Academic Achievements	0.117	183	0.222
			Acceptance	AI Training Experience	-0.224	183	0.023
			Acceptance	Fixed Creative Thinking	0.111	183	0.032
			Acceptance	Growing Creative Thinking	0.413	183	<0.001
			Acceptance	Attitude	0.456	183	<0.001
			Acceptance	Attitude	0.066	183	0.195
			Acceptance	Perceived Anxiety	-0.032	183	0.33
(Grassini et al. 2024)	UTAUT2	Education	Use Behavior(UB)	Behavioural Intention	0.579	104	<0.001
			Behavioral Intention	Performance Expectancy(PE)	0.495	104	<0.001

			Behavioral Intention	Habit	0.411	104	<0.001
(Hsiao et al. 2024)	TAM-NAM Model	Education	Behavioral Intention	Perceived Usefulness (PU)	0.37	336	<0.001
			Behavioral Intention	Perceived Ease of Use (PEU)	0.17	336	<0.05
			Behavioral Intention	Perceived Enjoyment	0.22	336	<0.001
			Behavioral Intention	Awareness of Consequences	-0.16	336	<0.01
			Behavioral Intention	Personal Innovativeness	0.14	336	<0.05
			Behavioral Intention	Self-Efficacy	0.18	336	<0.05
(Orhan et al. 2024)	GenAIAS	Education	Enjoyment	Performance Expectancy	0.79	461	<0.001
			Enjoyment	Effort Expectation	0.53	461	<0.001
			Enjoyment	Facilitating Conditions	0.65	461	<0.001
			Enjoyment	Social Influence	0.72	461	<0.001
			Interest	Performance Expectancy	0.84	461	<0.001
			Interest	Effort Expectation	0.65	461	<0.001
			Interest	Facilitating Conditions	0.7	461	<0.001
			Interest	Social Influence	0.67	461	<0.001
(Adžić et al. 2024)	self-constructed model	Education	Attitude	Use of ChatGPTUse of ChatGPT	0.514	229	<0.001
			Attitude	Usefulness of ChatGPT	0.514	229	<0.001
			Attitude	Cheating with ChatGPT	-0.103	229	0.113
			Attitude	Gender	0.416	237	0.005
(Ballesteros et al. 2024)	Extended UTAUT2	Education	Use Behavior(UB)	Behavioural Intention	0.443	772	<0.001
			Use Behavior(UB)	Effort Expectancy(EE)	0.148	772	0.003
			Behavioral Intention	Effort Expectancy(EE)	0.046	772	0.018
			Behavioral Intention	Performance Expectancy(PE)	0.626	772	<0.001
(Y. Yang et al. 2025)	TPACK Model and SDT	Education	Teachers' Willingness to Integrate Generative Artificial Intelligence (WIAI)	Technological pedagogical content knowledge TPACK	0.482	1348	<0.001
(Zhao et al. 2024)	Extended UTAUT2	Education	Use Behavior(UB)	Habit	0.197	500	<0.001
			Use Behavior(UB)	Facilitating Conditions(FC)	0.151	500	<0.001
			Behavioral	Facilitating	0.041	500	>0.05

			Intention	Conditions(FC)			
			Behavioral Intention	Personal Innovativeness	0.208	500	<0.001
			Behavioral Intention	Familiarize with technical knowledge FTK	0.281	500	<0.001
			Behavioral Intention	Perceived Ease of Use (PEU)	-0.057	500	>0.05
			Behavioral Intention	Social Influence（SI）	0.039	500	>0.05
			Behavioral Intention	Price Value(PV)	0.002	500	>0.05
			Behavioral Intention	Privacy Concerns	0.02	500	>0.05
			Behavioral Intention	Performance Expectancy(PE)	0.137	500	<0.01
			Behavioral Intention	Hedonic Motivation(HM)	0.142	500	<0.01
(C. Wang et al. 2024)	Interaction Hypothesis	Education	Acceptance	Interacting with D-ID Agent	1.31	99	<0.001
			Anxiety	Interacting with D-ID Agent	-0.7	99	<0.05
(Al-Abdullatif 2024)	TAM	Education	Acceptance	Perceived Ease of Use (PEU)	0.48	237	<0.001
			Acceptance	AI Literacy	0.35	237	<0.001
(Tang and Su 2025)	AIGCT-SI Model	Education	Behavioral Intention	Performance Expectancy(PE)	0.211	453	<0.001
			Behavioral Intention	Effort Expectancy(EE)	0.135	453	<0.01
			Behavioral Intention	Facilitating Conditions(FC)	0.1	453	<0.05
			Behavioral Intention	Learning Value	0.167	453	<0.01
			Behavioral Intention	Habit	0.137	453	<0.05
			Behavioral Intention	Trust	0.14	453	<0.05
			Trust	Information Accuracy	0.556	453	<0.001
			Trust	Privacy Concerns	-0.099	453	<0.05
			Trust	Social Influence（SI）	0.13	453	<0.01
			Trust	Effort Expectancy(EE)	0.149	453	<0.01
(Cabero-Almenara et al. 2024)	UTAUT2	Education	Behavioral Intention	Attitude	0.626	425	<0.005
			Behavioral Intention	Constructivist Pedagogical Beliefs	0.281	425	<0.005
			Behavioral Intention	Effort Expectancy(EE)	-0.028	425	<0.005
			Behavioral Intention	Facilitating Conditions(FC)	-0.031	425	<0.005

			Behavioral Intention	Hedonic Motivation(HM)	0.286	425	<0.005
			Behavioral Intention	Performance Expectancy(PE)	0.362	425	<0.005
			Behavioral Intention	Social Influence（SI）	0.305	425	<0.005
			Behavioral Intention	Transmissive Pedagogical Beliefs	0.411	425	<0.005
(F. Huang et al. 2024)	Self-Efficacy Theory and TAM	Education	Acceptance	EFL Learners' Well-Being	0.483	613	<0.001
			Acceptance	Self-Efficacy	0.388	613	<0.001
(Ramnarain et al. 2024)	TPB	Education	Behavioral Intention	Subjective Norms(SN)	0.3	42	<0.05
			Behavioral Intention	Concerns about Generative AI	0.29	42	<0.05
			Behavioral Intention	Perceived Usefulness (PU)	0.27	42	<0.05
			Behavioral Intention	Perceived Skill Readiness	0.27	42	<0.05
			Behavioral Intention	Perceived Behavioral Control(PBC)	0.381	42	<0.05
(Jang 2024)	UTAUT	Education	Behavioral Intention	AI Literacy	0.118	238	<0.01
			Behavioral Intention	Performance Expectancy(PE)	0.595	238	<0.001
			Behavioral Intention	Effort Expectancy(EE)	0.011	238	0.844
(Bouteraa et al. 2024)	TAM and SDT	Education	Continuance Usage Intention	Autonomy	0.577	240	<0.01
			Continuance Usage Intention	Perceived Usefulness (PU)	0.195	240	<0.05
(Bhaskar et al. 2024)	Extended TAM	Education	Behavioral Intention	Perceived Usefulness (PU)	0.37	476	<0.001
			Behavioral Intention	Perceived Ease of Use (PEU)	0.265	476	<0.001
			Behavioral Intention	Trust	0.382	476	<0.001
			Trust	Perceived Intelligence	0.232	476	<0.001
			Trust	Perceived Anthropomorphism	0.423	476	<0.001
			Trust	Social Influence（SI）	0.17	476	<0.001
(Salem et al. 2024)	BFI and GAAIS	Education	Attitude	Openness	0.499	218	<0.001
			Attitude	Neuroticism	-0.149	218	0.005
			Attitude	Agreeableness	-0.178	218	0.019
			Attitude	Agreeableness	0.372	218	<0.001
			Attitude	Conscientiousness	0.172	218	0.005
			Attitude	Neuroticism	0.16	218	0.011
(Lu et al.	TAM and TPB	Educ	Behavioral	Perceived Ease of	0.246、	457	<0.001

2024)		ation	Intention	Use (PEU)	0.307、0.264		
			Behavioral Intention	Perceived Usefulness (PU)	0.466、0.332、0.395	457	<0.001
			Behavioral Intention	Attitude	0.373	457	<0.001
			Behavioral Intention	Perceived Behavioral Control(PBC)	0.239{Citation}	457	<0.001
(M. Shahzad et al. 2024)	TAM	Education	Behavioral Intention	ChatGPT Consciousness CGPTAW	0.077	320	0.003
			Behavioral Intention	Perceived Ease of Use (PEU)	0.694	320	<0.001
			Behavioral Intention	Perceived Usefulness (PU)	0.082	320	0.004
			Behavioral Intention	Perceived Intelligence	0.196	320	0.007
(C. L. Wang et al. 2024)	Extended TPB	Education	Behavioral Intention	Attitude	0.624	245	<0.001
			Behavioral Intention	Perceived Behavioral Control(PBC)	0.26	245	<0.001
			Attitude	AI Literacy	0.398	245	<0.001
			Attitude	Subjective Norms(SN)	0.416	245	<0.001
(Hazaimeh and Al-Ansi 2024)	AI acceptance model	Education	Acceptance	Attitude	0.211	1152	<0.001
			Acceptance	Digital Competency	0.382	1152	<0.001
			Acceptance	Perceived Benefits	0.313	1152	<0.001
			Acceptance	Openness to Experience	0.118	1152	<0.001
(Syed et al. 2024)	No theoretical model introduced	Education	Use Behavior(UB)	Age	0.048	201	0.028
			Use Behavior(UB)	Gender	0.33	201	<0.001
			Use Behavior(UB)	Nationality	0.194	201	0.003
(Duong et al. 2024a)	ISS and SOR	Education	Continuance Usage Intention	Information Quality,	0.216	468	<0.001
			Continuance Usage Intention	Service Quality	0.244	468	<0.001
			Satisfaction	Information Quality	0.241	468	<0.001
			Satisfaction	Service Quality	0.343	468	<0.001
			Trust	Information Quality	0.142	468	0.001
			Trust	Service Quality	0.312	468	<0.001
(W. Li et al. 2024)	TAM	Education	Attitude	Perceived Usefulness (PU)	0.208	197	<0.05
			Attitude	Perceived Ease of Use (PEU)	0.228	197	<0.05

			Attitude	AI Anxiety (AIAX)	-0.13	197	<0.05
(Kocsis and Molnár 2025)	TAM	Education	Use Behavior(UB)	Behavioural Intention	0.99	911	<0.001
			Behavioral Intention	Attitude	1.02	911	<0.001
			Attitude	Perceived Usefulness (PU)	0.99	911	<0.001
(Ziemba et al. 2023)	UTAUT	Education	Use Behavior(UB)	Behavioural Intention	0.571	237	<0.05
			Use Behavior(UB)	Facilitating Conditions(FC)	0.149	237	<0.05
			Behavioral Intention	Effort Expectancy(EE)	0.207	237	<0.05
			Behavioral Intention	Social Influence（SI）	0.227	237	<0.05
			Behavioral Intention	Performance Expectancy(PE)	0.44	237	<0.05
(Chan and Hu, 2023)	No theoretical model introduced	Education	Acceptance	Knowledge of GenAI	0.189	399	<0.001
			Acceptance	Frequency of Use	0.326	399	<0.001
(Y. Wang and Zhang 2023)	UTAUT2	Creative industries	Behavioral Intention	Innovativeness	0.187	326	<0.001
			Behavioral Intention	Performance Expectancy(PE)	0.102	326	>0.05
			Behavioral Intention	Effort Expectancy(EE)	0.229	326	<0.001
			Behavioral Intention	Hedonic Motivation(HM)	0.256	326	<0.001
			Behavioral Intention	Price Value(PV)	0.246	326	<0.001
			Behavioral Intention	Curiosity	0.22	326	<0.001
			Creativity	Trait Curiosity	0.558	326	<0.001
(Yin et al. 2023)	UTAUT2	Creative industries	Behavioral Intention	Performance Expectancy(PE)	0.15	347	<0.01
			Behavioral Intention	Effort Expectancy(EE)	0.09	347	<0.05
			Behavioral Intention	Social Influence（SI）	0.16	347	<0.001
			Behavioral Intention	Hedonic Motivation(HM)	0.16	347	<0.01
			Behavioral Intention	Habit	0.39	347	<0.001
			Behavioral Intention	AI Learning Anxiety (AL)	0.14	347	<0.001
			Behavioral Intention	AI Configuration Anxiety (AC)	-0.19	347	<0.001

			Behavioral Intention	AI Configuration Anxiety (AC)	0.05	347	<0.05
			Behavioral Intention	Job Replacement Anxiety (JP)	0.06	347	<0.001
(Latikka et al. 2023)	SDT	Creative industries	Attitude	Perceived Autonomy	0.04	828	0.004
			Attitude	Perceived Relatedness	0.05	828	0.001
(Zhou and Zhang 2024a)	Multiple	Creative industries	Concern	Flow Experience	0.292	529	<0.001
			Concern	Attachment	0.439	529	<0.001
			Concern	Flow Experience	0.398	529	<0.001
			Concern	Perceived Anthropomorphism	0.212	529	<0.001
			Concern	Personalization	0.256	529	<0.001
(Sohn et al. 2021)	Consumption Value Theory	Creative industries	Purchase Intentions	Social value	0.476	163	<0.01
			Purchase Intentions	Epistemic value	0.584	163	<0.001
			Behavioral Intention	Gender	-0.12	1298	<0.001
(C. Wang 2024)	Multiple	Creative industries	Attitude	Behavioural Intention	0.755	313	<0.001
			Attitude	Satisfaction	0.245	443	<0.001
			Attitude	Flow Experience	0.226	443	<0.001
(Y. Wang et al. 2025)	UTAUT and Affective-Cognitive Coherence Theory	Creative industries	Behavioral Intention	Social Influence（SI）	0.317	443	<0.001
			Behavioral Intention	Perceived Usefulness (PU)	0.215	443	0.001
			Behavioral Intention	Perceived enjoyment (PE)	0.132	443	0.045
			Behavioral Intention	Effort Expectancy(EE)	-0.098	443	0.08
(Yu et al. 2024)	ECM,TAM,UTAUT and Flow Theory	Creative industries	Use Behavior(UB)	Personal Innovativeness	-0.013	443	0.775
			Continuance Usage Intention	Confirmation	0.97	443	<0.001
			Continuance Usage Intention	Perceived Usefulness (PU)	-0.113	443	0.128
			Continuance	Perceived Ease of	0.011	443	0.768

			Usage Intention	Use (PEU)			
			Continuance Usage Intention	Confirmation	0.811	443	<0.001
			Continuance Usage Intention	Confirmation	0.406	443	<0.001
			Continuance Usage Intention	Confirmation	0.808	443	<0.001
			Satisfaction	Confirmation	0.735	443	<0.001
			Satisfaction	Confirmation	0.671	443	<0.001
			Satisfaction	Personal Innovativeness	0.611	443	<0.001
			Satisfaction	Perceived enjoyment	0.808	443	<0.001
			Continuance Usage Intention	AI Anxiety (AIAX)	-0.12	9	<0.05
			Attitude	Social Influence（SI）	0.12	9	<0.05
			Attitude	Facilitating Conditions(FC)	0.12	9	<0.05
			Attitude	Attitude	0.12	9	<0.05
			Attitude	Behavioural Intention	0.12	9	<0.05
(X. Li et al. 2024)	Extended UTAUT2	Creati ve indus tries	Attitude	Performance Expectancy(PE)	0.274	386	<0.001
			Attitude	Effort Expectancy(EE)	0.286	386	<0.001
			Attitude	Social Influence（SI）	0.171	386	0.013
			Behavioral Intention	Facilitating Conditions(FC)	0.314	386	<0.001
			Behavioral Intention	Perceived risk	-0.307	386	<0.001
			Use Behavior(UB)	Commercial Value(CV)	0.181	386	<0.001
			Use Behavior(UB)	Commercial Value(CV)	0.149	386	0.002
			Behavioral Intention	Commercial Value(CV)	0.157	386	<0.001
(Qiu et al. 2024)	UTAUT2 and IDT	Creati ve indus tries	Behavioral Intention	Behavioural Intention	0.241	394	<0.001
			Behavioral Intention	Social System	0.237	394	<0.01
			Behavioral Intention	Individual Innovativeness	0.289	394	<0.01
			Behavioral Intention	Communication Channels	0.1	394	<0.05
			Behavioral Intention	Performance Expectancy(PE)	0.109	394	<0.05
			Use	Effort	0.096	394	<0.05

			Behavior(UB)	Expectancy(EE)			
			Use Behavior(UB)	Social System	0.127	394	<0.05
			Use Behavior(UB)	Individual Innovativeness	0.128	394	<0.05
			Use Behavior(UB)	Communication Channels	0.133	394	<0.01
			Use Behavior(UB)	AI Anxiety (AIAX)	0.122	394	<0.05
(Jiang et al. 2024)	UTAUT,DITand self-efficacy theory	Creative industries	Behavioral Intention	Performance Expectancy(PE)	0.516	284	<0.000
			Behavioral Intention	Performance Expectancy(PE)	0.232	284	0.035
			Behavioral Intention	Effort Expectancy(EE)	0.132	284	0.056
			Behavioral Intention	Social Influence（SI）	-0.021	284	0.756
			Behavioral Intention	Facilitating Conditions(FC)	0.087	284	0.275
			Behavioral Intention	Compatibility	-0.027	284	0.637
			Behavioral Intention	Self-Efficacy	0.02	284	0.747
(S. F. Wang and Chen 2024b)	SOR framework, TAM,TPB and self-efficacy theory	Creative industries	Behavioral Intention	Subjective Norms(SN)	0.034	226	>0.05
			Behavioral Intention	Self-Efficacy	-0.261	226	>0.05
			Behavioral Intention	Subjective Norms(SN)	0.277	226	<0.05
			Behavioral Intention	Facilitating Conditions(FC)	1.049	226	<0.05
			Behavioral Intention	Self-Efficacy	0.963	226	<0.05
			Behavioral Intention	Technology Anxiety	-0.017	226	>0.05
			Behavioral Intention	Perceived Ease of Use (PEU)	-0.066	226	>0.05
			Behavioral Intention	Perceived Usefulness (PU)	0.134	226	>0.05
			Behavioral Intention	Subjective Norms(SN)	0.097	226	>0.05
(Sohn and Kwon 2020)	TAM,UTAUT,TPB,VAM	Creative industries	Behavioral Intention	Facilitating Conditions(FC)	0.045	226	>0.05
			Behavioral Intention	Facilitating Conditions(FC)	0.433	226	<0.05
			Behavioral Intention	Self-Efficacy	0.595	226	<0.05

			Anxiety	Self-Efficacy	-0.335	226	>0.05
			Anxiety	Technology Anxiety	-0.021	226	>0.05
(Jang et al. 2024)	Experimentation	Creative industries	Acceptance	Additional explanation	-1.19	500	<0.01
			Acceptance	Arrangement	0.81	500	<0.01
			Acceptance	Global explanation	0.9	500	<0.01
			Acceptance	Local explanation	0.5	500	<0.01
			Acceptance	Accuracy	0.35	500	<0.01
			Acceptance	Price	-1.3	500	<0.01
(S. F. Wang and Chen 2024a)	TAM and TPB	Creative industries	Behavioral Intention	Trust	0.118	184	<0.001
			Trust	Functional Risk	0.138	184	0.047
			Concern	Perceived Ease of Use (PEU)	0.08	184	0.772
			Concern	Perceived Ease of Use (PEU)	0.1	184	<0.001
			Concern	Perceived Usefulness (PU)	0.11	184	<0.001
(Zhou and Zhang 2024b)	Extended UTAUT	Creative industries	Concern	Flow Experience	0.456	529	<0.05
			Concern	Attachment	0.439	529	<0.05
			Acceptance	Perceived Anthropomorphism	0.282	529	<0.05
			Acceptance	Personalization	0.306	529	<0.05
(K.-L. Huang et al. 2024)	HOTS	Creative industries	Acceptance	Competency Levels	0.158（PU）, 0.175（ATU）, 0.063（BI）	119	<0.05
(H. Ma and Li 2024)	ECT	Creative industries	Continuance Usage Intention	Satisfaction	0.45	338	<0.05
			Continuance Usage Intention	Perceived Usefulness (PU)	0.211	338	<0.05
			Continuance Usage Intention	Self-Efficacy	0.209	338	<0.05
			Continuance Usage Intention	Trust	0.126	338	<0.05
			Satisfaction	Perceived Usefulness (PU)	0.329	338	<0.05
			Satisfaction	Self-Efficacy	0.15	338	<0.05
(Skripchuk et al. 2024)	TPB	Creative	Attitude	Perceived Usefulness (PU)	0.305（Web）/	152	<0.000

		industries	Attitude	Punishment Certainty	0.267（GenAI）-0.337（Web）/-0.279（GenAI）	152	<0.000
(Maican et al. 2023)	UTAUT2	Organization	Behavioral Intention	Effort Expectancy (EE)	-0.748	403	0.012
			Behavioral Intention	Habit	0.572	403	0.008
			Behavioral Intention	Performance Expectancy(PE)	0.689	403	0.003
			Behavioral Intention	Social Influence（SI）	-0.454	403	0.025
			Behavioral Intention	Hedonic Motivation (HM)	0.175	403	0.444
			Behavioral Intention	Facilitating Conditions(FC)	0.095	403	0.659
			Behavioral Intention	Perceived Customer Value (PCV)	-0.045	403	0.821
(Agrawal 2023)	Techno-organizational-environmental framework, institutional theory and innovation diffusion theory.	Organization	Use Behavior(UB)	Compatibility	1.08	108	<.05
			Use Behavior(UB)	Complexity	-2.63	108	<.001
			Use Behavior(UB)	Organizational Size	0.59	108	<.01
			Use Behavior(UB)	Regulatory Support	-3.52	108	<.01
			Use Behavior(UB)	Competition Intensity	0.82	108	<.05
			Use Behavior(UB)	Environmental Uncertainty	0.79	108	<.05
(Tanantong and Wongras 2024)	UTAUT	Organization	Behavioral Intention	Effort Expectancy(EE)	0.165	364	0.001
			Behavioral Intention	Trust	0.06	364	0.201
			Behavioral Intention	Facilitating Conditions(FC)	0.084	364	0.027
			Behavioral Intention	Perceived Value(PV)	0.269	364	<0.001
			Behavioral Intention	Perceived Autonomy PA	0.257	364	<0.001

			Behavioral Intention	Agile Leadership AL	0.071	364	0.287
(Cimino et al. 2024)	AST	Organization	Use Behavior(UB)	Agile Leadership AL	0.135	222	0.037
			Use Behavior(UB)	Innovation Orientation IO	0.167	222	0.027
			Use Behavior(UB)	Individual Creativity IC	0.153		0.043
(W. Chang and Park 2024)	Trust Transfer Effect Theory and Consumer Decision-Making Process	Organization	Trust	Trust	0.29	443	<0.001
			Trust	Performance Expectancy(PE)	0.732	443	<0.001
			Trust	Performance Expectancy(PE)	0.49	443	<0.001
(Cintamür 2024)	AIDUA	bank	Attitude	Performance Expectancy(PE)	0.348	443	<0.000
			Attitude	Effort Expectancy(EE)	-0.244	575	<0.000
			Acceptance	Emotion	0.658	575	<0.000
			Concern	Emotion	-0.371	575	<0.000
(Jiang et al. 2024)	UTAUT and TRA	bank	Attitude	Performance Expectancy(PE)	0.168	638	<0.001
			Attitude	Effort Expectancy(EE)	0.053	638	<0.05
			Attitude	Social Influence（SI）	0.128	638	<0.01
			Attitude	Facilitating Conditions(FC)	0.245	638	<0.001
			Attitude	Attitude	0.352	638	<0.001
			Attitude	Facilitating Conditions(FC)	0.242	638	<0.001
			Trust	Perceived security	0.574	638	<0.001
			Trust	Perceived privacy	0.227	638	<0.001
			Attitude	Trust	0.274	638	<0.001
			Behavioral Intention	Trust	0.282	638	<0.001
(Figueiredo et al. 2024)	UTAUT and SPINNER model	Organization	Creativity	Use behavior	0.555	124	<0.000
			Creativity	Facilitating Conditions(FC)	0.383	124	<0.000
			Behavioral Intention	Private Knowledge, PRVKM	0.348	124	<0.000
			Behavioral Intention	Public Knowledge, PUBKM	0.312	124	0.003
			Behavioral Intention	Facilitating Conditions(FC)	0.206	124	0.13
(Zhang et al. 2024)	social network theory	Organizati	Creativity	Employee-AI Instrumental Ties	0.486	243	<0.000

		Organization					
			Creativity	Employee-AI Expressive Ties	0.352	243	<0.000
(Huynh 2024)	TCV and PCT	Organization	Trust	Functional value	0.442	211	<0.001
			Trust	Social value	0.123	211	<0.05
			Trust	Emotional value	0.143	211	<0.05
			Trust	Epistemic value	0.206	211	<0.01
			Trust	Information sensitivity	-0.231	211	<0.001
			Trust	Information control	0.5	211	<0.001
			Acceptance	Trust	0.563	211	<0.001
(Y. Kim et al. 2024)	UTAUT	Organization	Use Behavior(UB)	Behavioural Intention	0.863	300	<0.001
			Behavioral Intention	Effort Expectancy(EE)	0.174	300	<0.05
			Behavioral Intention	Social Influence（SI）	0.662	300	<0.001
			Behavioral Intention	Performance Expectancy(PE)	0.072	300	>0.05
			Behavioral Intention	Facilitating Conditions(FC)	0.037	300	>0.05
(Prasad et al. 2024)	TAM	Organization	Attitude	Optimism	0.416	500	<0.001
			Attitude	Innovativeness	0.081	500	<0.001
(Korzyński et al. n.d.)	UTAUT	Organization	Use Behavior(UB)	Effort Expectancy(EE)	0.113	128	0.095
			Use Behavior(UB)	Facilitating Conditions(FC)	0.149	128	0.034
			Use Behavior(UB)	Trust	0.353	128	<0.001
			Use Behavior(UB)	Top Management Support via Trust in AI	0.137	128	0.002
(Felicetti et al. 2024)	Adaptive Structuration Theory(AST)	Organization	Creativity	Innovation Attitude, IA	0.32	131	<0.001
			Creativity	Peer Influence	0.147	131	0.044
			Creativity	Task-Technology Fit, TTF	0.458	131	<0.001
			Concern	Innovation Attitude, IA	0.388	131	<0.001
			Concern	Peer Influence	0.164	131	0.008
			Concern	Task-Technology Fit, TTF	0.433	131	<0.001
(Marimon et al. 2024)	Job Demands-Resources Model, TAM, TRI, Trust	Organization	Trust	User Experience	0.86	251	<0.001

	Theory and Work Engagement Theory						
(Rana et al. 2024)	Institutional Theory(INST), FATAA Ethical Principles	Organization	Use Behavior(UB)	Coercive Pressure	0.149	384	0.036
			Use Behavior(UB)	Normative Pressure	0.208	384	0.004
			Use Behavior(UB)	Mimetic Pressure	0.321		0.001
			Use Behavior(UB)	Fairness	0.288	384	0.002
			Use Behavior(UB)	Accountability	0.298	384	0.002
			Use Behavior(UB)	Transparency	0.217	384	0.003
			Use Behavior(UB)	Accuracy	0.302	384	0.003
			Use Behavior(UB)	Autonomy	0.276	384	0.003
(de Vreede and de Vreede n.d.)	SCT	Organization	Behavioral Intention	AI Engagement	0.48	258	<0.001
			Behavioral Intention	AI Familiarity	0.21	258	<0.01
(B. Yang et al. 2024)	Cognitive Fit Theory and Satisfaction Attainment Theory	Organization	Satisfaction	Cognitive Fit	0.592	548	<0.001
			Satisfaction	Cognitive Fit	0.289	548	<0.001
			Satisfaction	Satisfaction with Outcome	0.552	548	<0.001
			Behavioral Intention	Decision-making	0.565	319	<0.001
(Stevens and Stetson 2023)	TrAAIT	Healthcare	Acceptance	Trust	0.697	73	<0.001
			Trust	Information Credibility INFC	0.996	73	<0.001
			Trust	Performance Expectancy(PE)	0.998	73	<0.001
			Trust	Application Value (APPV)	0.995	73	<0.001
			Trust	Trust	0.629	73	<0.001
(Hou et al. 2024)	Experimentation	Healthcare	Acceptance	AISmartness	0.586	583	<0.01
			Acceptance	Transparency	0.394	583	<0.01
(X. Wang and Wang 2024)	UTAUT	Healthcare	Behavioral Intention	Initial Trust	0.574	190	<0.001

			Behavioral Intention	Performance Expectancy(PE)	0.174	190	<0.01
			Trust	Effort Expectancy(EE)	0.243	190	<0.001
			Trust	Trust Tendency	0.307	190	<0.001
			Trust	Social Influence（SI）	0.228	190	<0.001
(L. Li et al. 2024)	UTAUT	Healt hcare	Behavioral Intention	Performance Expectancy(PE)	0.6	208	<0.001
			Behavioral Intention	Effort Expectancy(EE)	-0.28	208	<0.05
			Behavioral Intention	Facilitating Conditions(FC)	0.12	208	>0.05
			Behavioral Intention	Price Value(PV)	0.3	208	<0.01
			Behavioral Intention	Descriptive Norm	0.3	208	<0.001
			Behavioral Intention	Injunctive Norm	-0.09	208	>0.05
			Behavioral Intention	Self-stigma	-0.09	208	>0.05
			Behavioral Intention	Social stigma	0.12	208	>0.05
			Behavioral Intention	AI Hesitancy	-0.19	208	<0.05
			Behavioral Intention	Privacy Concerns	0.02	208	>0.05
			Continuance Usage Intention	Performance Expectancy(PE)	0.46	185	<0.001
			Continuance Usage Intention	Effort Expectancy(EE)	0.09	185	>0.05
			Continuance Usage Intention	Facilitating Conditions(FC)	-0.12	185	>0.05
			Continuance Usage Intention	Price Value(PV)	0.23	185	<0.01
			Continuance Usage Intention	Descriptive Norm	0.21	185	<0.01
			Continuance Usage Intention	Injunctive Norm	0.19	185	<0.05
			Continuance Usage Intention	Self-stigma	0.01	185	>0.05
			Continuance Usage Intention	Social stigma	0.07	185	>0.05
			Continuance Usage Intention	AI Hesitancy	-0.11	185	>0.05
			Continuance Usage Intention	Privacy Concerns	-0.02	185	>0.05
(Andrews 2021)	UTAUT	Acad emic resear ch	Behavioral Intention	Habit	0.361	629	<0.01
			Behavioral Intention	Performance Expectancy(PE)	0.351	629	<0.01

			Behavioral Intention	Hedonic Motivation(HM)	0.199	629	<0.01
			Behavioral Intention	Social Influence（SI）	0.083	629	<0.01
			Behavioral Intention	Personal Innovativeness	0.087	629	<0.01
(Pallivathukal et al. 2024)	NA	Academic research	Use Behavior(UB)	Year of study	1.22	443	0.002
			Use Behavior(UB)	Knowledge	0.13	443	0.014
			Use Behavior(UB)	Attitude	0.04	443	<0.001
(Gu et al. 2024)	SOR	Creative industries	Concern	Verisimilitude	-0.186	1147	<0.001
			Concern	Vitality	-0.053	1147	0.179
			Concern	Imagination	-0.196	1147	<0.001
			Concern	Synthesis	0.484	1147	<0.001
			Acceptance	Perceived Eeriness	-0.256	1147	<0.001
			Acceptance	Perceived Intelligence	0.628	1147	<0.001
(Russo 2024)	TAM, DIT and SCT	Creative industries	Behavioral Intention	Perceptions about the Technology (PT)	0.155	184	0.204
			Behavioral Intention	Compatibility Factors	0.536	184	<0.001
			Behavioral Intention	Social Factors (SF)	0.087	184	0.17
(Spatscheck et al. 2024)	Experimentation	Healthcare	Trust	Anthropomorphism	-0.301	368	0.021
			Trust	Advice Elaboration	0.417	368	<0.001
			Concern	Anthropomorphism	-0.27	368	0.039
(H.-L. Lin et al. 2025)	TAM	Healthcare	Use Behavior(UB)	Perceived risk	-0.259	111	0.049
			Use Behavior(UB)	Perceived Usefulness (PU)	0.662	111	<0.001
(R. R. Lin et al. 2023)	UTAUT	bank	Acceptance	Performance Expectancy(PE)	0.598	428	<0.001
			Acceptance	Social Influence（SI）	0.138	428	<0.1
			Acceptance	Cognitive Factors	0.027	428	>0.05
			Acceptance	Effort Expectancy(EE)	0.018	428	>0.05
			Acceptance	Facilitating Conditions(FC)	-0.112	428	>0.05
(Martínez Puertas et al. 2024)	UGT	consumer	Purchase Intentions	Entertainment	0.289	173	<0.001

			Purchase Intentions	Social Presence	0.258	173	<0.01
			Purchase Intentions	Media Appeal	0.218	173	<0.05
			Purchase Intentions	Risk to Privacy	-0.06	173	<0.05
(Lee and Chen 2022)	SOR	bank	Behavioral Intention	Perceived Intelligence	0.483	451	<0.001
			Behavioral Intention	Perceived Anthropomorphism	0.367	451	<0.001
			Behavioral Intention	Task Technology Fit(TTF)	0.292	451	<0.001
			Behavioral Intention	Trust	0.549	451	<0.001
			Trust	Perceived Intelligence	0.304	451	<0.001
			Trust	Perceived Anthropomorphism	0.303	451	<0.001
(Baek and Kim 2023)	UGT	service	Continuance Usage Intention	Personalization	0.522	421	<0.01
			Continuance Usage Intention	Task Efficiency	0.291	421	<0.10
			Continuance Usage Intention	Information Seeking	-0.021	421	>0.10
			Continuance Usage Intention	Social Interaction	0.237	421	>0.10
			Continuance Usage Intention	Playfulness	0.041	421	>0.10
			Continuance Usage Intention	Trust	0.187	421	<0.01
			Continuance Usage Intention	Creepiness	-0.704	421	<0.01
			Concern	Personalization	-0.437	421	<0.10
			Concern	Task Efficiency	0.607	421	<0.05
			Concern	Social Interaction	0.229	421	<0.05
			Concern	Information Seeking	-0.171	421	>0.10
			Concern	Playfulness	0.321	421	>0.10
			Trust	Personalization	0.522	421	<0.01
			Trust	Task Efficiency	0.291	421	<0.10
			Trust	Information Seeking	-0.021	421	>0.10
			Trust	Social Interaction	0.237	421	>0.10
			Trust	Playfulness	0.041	421	>0.10
(Camilleri 2024)	UTAUT,ISST,ELM and IAM	service	Behavioral Intention	Performance Expectancy(PE)	0.236	654	0.002
			Behavioral Intention	Effort Expectancy(EE)	0.134	654	0.006
			Behavioral Intention	Social Influence（SI）	0.263	654	<0.001
			Behavioral	Perceived	0.355	654	<0.001

			Intention	Interactivity			
(Nan et al. 2024)	ISST and ECM	service	Behavioral Intention	Performance Expectancy(PE)	0.341	694	<0.001
			Behavioral Intention	Social Influence（SI）	0.117	694	<0.001
			Behavioral Intention	Hedonic Motivation (HM)	0.243	694	<0.001
			Behavioral Intention	Habit	0.409	694	<0.001
			Behavioral Intention	Personal Innovativeness	0.091	694	<0.001
			Behavioral Intention	Effort Expectancy(EE)	0.002	694	0.961
			Behavioral Intention	Facilitating Conditions(FC)	-0.022	694	0.652
			Behavioral Intention	Price Value(PV)	0.02	694	0.561
(Nan et al. 2024)	Information System Success Theory	service	Continuance Usage Intention	Information Quality	0.398	252	<0.001
			Continuance Usage Intention	System Quality	0.532	252	<0.001
			Continuance Usage Intention	Privacy Concerns	-0.116	252	0.008
			Continuance Usage Intention	Perceived Innovativeness	0.244	252	<0.001
			Intention to Recommend	Information Quality	0.398	252	<0.001
			Intention to Recommend	System Quality	0.532	252	<0.001
			Intention to Recommend	Privacy Concerns	-0.116	252	0.008
			Intention to Recommend	Perceived Innovativeness	0.244	252	<0.001
(J. S. Kim et al. 2024)	SOR, TAM amd attachment theory	service	Satisfaction	Perceived Ease of Use (PEU)	0.254	446	<0.01
			Satisfaction	Perceived Enjoyment	0.438	446	<0.01
			Satisfaction	perceptual responsiveness	0.052	446	>0.05
			Concern	Perceived Enjoyment	0.702	446	<0.01
			Concern	Perceived Ease of Use (PEU)	0.006	446	>0.05
			Concern	perceptual responsiveness	-0.112	446	>0.05
			Continuance Usage Intention	Attachment	0.405	446	<0.01
			Continuance Usage Intention	Satisfaction	0.447	446	<0.01
(Xia and Chen 2024)	UTAUT	service	Attitude	Self-Efficacy	0.209	309	0.001

			Behavioral Intention	Task-Tool Fitness	0.578	309	<0.001
			Behavioral Intention	Price Value(PV)	0.231	309	<0.001
			Behavioral Intention	Effort Expectancy(EE)	0.215	309	<0.001
			Behavioral Intention	Facilitating Conditions(FC)	0.21	309	<0.001
(Marjerison et al. 2022)	UGT	service	Behavioral Intention	Information Support	0.187	351	0.001
			Behavioral Intention	Knowledge Acquisition	0.291	351	<0.001
			Behavioral Intention	Utilitarian Benefits	0.4	351	<0.001
			Use Behavior(UB)	Behavioural Intention	0.51	351	<0.001
			Use Behavior(UB)	Gender	-0.181	351	0.037
			Use Behavior(UB)	Age	-0.099	351	0.021
(Xie et al. 2024)	UGT	service	Use Behavior(UB)	Utilitarian Gratification	0.71	3156	<0.001
			Satisfaction	Technology Gratification	0.58	2205	<0.001
			Satisfaction	Hedonic Gratification	0.66	2280	<0.001
			Satisfaction	Social Gratification	0.58	1665	<0.001
(Xiong et al. 2024)	UTAUT	service	Attitude	Performance Expectancy(PE)	0.53	926	<0.001
			Attitude	Effort Expectancy(EE)	0.58	926	<0.001
			Attitude	Trust	0.38	926	<0.01
			Attitude	Perceived risk	-0.39	926	<0.001
			Behavioral Intention	Performance Expectancy(PE)	0.42	926	<0.01
			Behavioral Intention	Effort Expectancy(EE)	0.5	926	<0.001
			Behavioral Intention	Social Influence（SI）	0.39	926	<0.01
			Behavioral Intention	Facilitating Conditions(FC)	0.42	926	<0.01
			Behavioral Intention	Trust	0.68	926	<0.001
			Behavioral Intention	Perceived risk	-0.6	926	<0.001
			Behavioral Intention	Attitude	0.73	926	<0.001
(Joshi 2021)	Extended UTAUT	consumer	Behavioral Intention	Effort Expectancy(EE)	-0.109	60	0.427
			Behavioral Intention	Performance Expectancy(PE)	0.479	60	<0.001
			Behavioral Intention	Social Influence（SI）	0.195	60	0.068

			Behavioral Intention	Facilitating Conditions(FC)	0.163	60	0.194
			Behavioral Intention	Perceived risk	-0.107	60	0.219
			Behavioral Intention	Satisfaction	0.16	60	0.333
			Behavioral Intention	Satisfaction	0.278	60	0.114
			Satisfaction	Facilitating Conditions(FC)	0.348	60	0.001
			Satisfaction	Trust	0.458	60	<0.001
			Trust	Perceived risk	-0.45	60	<0.001
			Satisfaction	Perceived risk	0.16	60	0.333
			Satisfaction	Behavioural Intention	0.213	60	0.213
			Satisfaction	Facilitating Conditions(FC)	0.245	60	0.167
(Chakraborty et al. 2024)	ELM and SQB	consumer	Attitude	Perceived Interaction Quality	0.376	372	<0.001
			Attitude	Perceived Credibility	0.359	372	<0.001
			Attitude	Perceived Inertia (PEI)	-0.248	372	<0.001
			Attitude	Perceived Inertia (PEI)	-0.106	372	0.033
			Attitude	Perceived Regret Avoidance (PRA)	-0.104		0.08
			Trust	Attitude	0.44	372	<0.001
(Duong et al. 2024b)	Parasocial Interaction Theory	journey	Satisfaction	Parasocial Interaction	0.396	606	<0.001
			Behavioral Intention	Parasocial Interaction	0.308	606	0.009
			Behavioral Intention	Satisfaction	0.281	606	<0.001
(S. Li et al. 2024)	Extended TAM	journey	Attitude	Perceived Ease of Use (PEU)	0.188	491	<0.01
			Attitude	Perceived Usefulness (PU)	0.393	491	<0.001
			Attitude	Perceived Enjoyment	0.122	491	<0.05
			Attitude	Perceived coolness	0.266	491	<0.001
			Behavioral Intention	Perceived Usefulness (PU)	0.376	491	<0.001
			Behavioral Intention	Perceived Enjoyment	0.271	491	<0.001
(Gursoy et al. 2019)	AIDUA	consumer	Attitude	Perceived Ease of Use (PEU)	0.188	491	<0.01
			Attitude	Perceived Usefulness (PU)	0.393	491	<0.001
			Behavioral Intention	Perceived Usefulness (PU)	0.376	491	<0.001
			Attitude	Perceived Ease of	0.414	491	<0.001

				Use (PEU)			
			Attitude	Perceived coolness	0.266	491	<0.001
			Attitude	Perceived Enjoyment	0.122	491	<0.05
			Behavioral Intention	Perceived Enjoyment	0.271	491	<0.001
(H. Lin et al. 2020)	AIDUA	consu mer	Acceptance	Social Influence（SI）	0.14	605	0.008
			Acceptance	Hedonic Motivation (HM)	0.73	605	<0.001
			Acceptance	Positive Emotion	0.88	605	<0.001
			Concern	Social Influence（SI）	-0.271	605	ns
			Concern	Positive Emotion	-0.53	605	<0.001
			Attitude	Performance Expectancy(PE)	0.21	605	<0.001
			Attitude	Effort Expectancy(EE)	-0.08	605	0.009
(Chi et al. 2023)	AIDUA	consu mer	Acceptance	Trust	0.32	986	<0.001
			Concern	Trust	-0.26	986	<0.001
			Trust	Performance Expectancy(PE)	0.35	986	<0.001
			Trust	Effort Expectancy(EE)	-0.19	986	<0.001
			Trust	Hedonic Motivation (HM)	0.39	986	<0.001
			Attitude	Effort Expectancy(EE)	-0.32	986	<0.001
			Attitude	Performance Expectancy(PE)	0.446	986	<0.001
			Attitude	Hedonic Motivation (HM)	0.768	986	<0.001
(Wong et al. 2023)	AIDUA	journ ey	Attitude	Performance Expectancy(PE)	0.59	499	<0.001
			Attitude	Effort Expectancy(EE)	-0.24	499	<0.001
			Acceptance	Emotions	0.69	499	<0.001
			Concern	Emotions	-0.34	499	<0.001
(Vitezić and Perić 2021)	AIDUA	journ ey	Acceptance	Emotions	0.788	786	<0.000
			Attitude	Effort Expectancy(EE)	-0.025	786	0.402
			Attitude	Performance Expectancy(PE)	0.218	786	<0.000
(Vitezić and Perić 2021)	Extaned UTAUT2	consu mer	Behavioral Intention	Performance Expectancy(PE)	0.25	411	<0.001
			Behavioral Intention	Facilitating Conditions(FC)	0.19	411	<0.001
			Behavioral Intention	Hedonic Motivation (HM)	0.32	411	<0.001
			Behavioral Intention	Information Quality	0.23	411	<0.1

			Behavioral Intention	Trust	0.2	411	<0.05
			Behavioral Intention	Mobility	0.19	411	<0.001
			Trust	Privacy Concerns	-0.24	411	<0.001
			Trust	Interface	0.6	411	<0.001
			Trust	Equipment	0.14	411	<0.01
(de Andrés-Sánchez and Gené-Albesa 2023)	UTAUT	bank	Behavioral Intention	Effort Expectancy(EE)	0.288	226	<0.01
			Behavioral Intention	Social Influence（SI）	0.339	226	<0.01
			Behavioral Intention	Trust	0.23	226	<0.05
			Behavioral Intention	Performance Expectancy(PE)	0.034	226	>0.05（ns）
(Sohn and Kwon 2020)	TAM,TPB and UTAUT	consumer	Behavioral Intention	Perceived Usefulness (PU)	0.354	378	<0.01
			Behavioral Intention	Perceived Ease of Use (PEU)	0.558	378	<0.01
			Behavioral Intention	Attitude	0.202	378	<0.01
			Behavioral Intention	Perceived Behavioral Control(PBC)	0.323	378	<0.01
			Behavioral Intention	Subjective Norms(SN)	0.474	378	<0.01
			Behavioral Intention	Performance Expectancy(PE)	0.303	378	<0.01
			Behavioral Intention	Effort Expectancy(EE)	0.315	378	<0.01
			Behavioral Intention	Social Influence（SI）	0.396	378	<0.01
			Behavioral Intention	Enjoyment,	0.631	378	<0.01
			Behavioral Intention	Technicality,	-0.132	378	<0.01
			Behavioral Intention	Perceived Value(PV)	0.255	378	<0.01
(Balakrishnan et al. 2022)	Meta-UTAUT	consumer	Attitude	Performance Expectancy(PE)	0.283	420	<0.001
			Attitude	Effort Expectancy(EE)	0.355	420	<0.001
			Attitude	Facilitating Conditions(FC)	0.169	420	<0.01
			Attitude	Social Influence（SI）	0.212	420	<0.001
			Attitude	Perceived Intelligence	0.411	420	<0.001
			Attitude	Perceived Anthropomorphis	0.382	420	<0.001

				m			
			Continuance Usage Intention	Performance Expectancy(PE)	0.207	420	<0.001
			Continuance Usage Intention	Effort Expectancy(EE)	0.282	420	<0.001
			Continuance Usage Intention	Facilitating Conditions(FC)	0.184	420	<0.01
			Continuance Usage Intention	Perceived Intelligence	0.384	420	<0.001
			Continuance Usage Intention	Perceived Anthropomorphism	0.336	420	<0.01
			Continuance Usage Intention	Attitude	0.454	420	<0.001
(K. P. Gupta and Pande 2022)	UTAUT and HRI	consumer	Trust	Anthropomorphism	0.2	419	<0.001
			Trust	Performance Expectancy(PE)	0.46	419	<0.001
			Trust	Effort Expectancy(EE)	0.34	419	<0.001
			Acceptance	Performance Expectancy(PE)	0.39	419	<0.001
			Acceptance	Trust	0.47	419	<0.001
			Acceptance	Effort Expectancy(EE)	0.22	419	<0.001
(Manzoor et al. 2024)	AIDUA	journey	Attitude	Performance Expectancy(PE)	0.065	463	<0.05
			Attitude	Effort Expectancy(EE)	-0.064	463	<0.05
			Behavioral Intention	Emotions	0.541	463	<0.001
			Concern	Emotions	-0.964	463	<0.01
			Behavioral Intention	Emotions	0.231	463	<0.05
			Concern	Emotions	-0.113	463	<0.01
(“Full article: The effects of generative AI’s human-like competencies on clinical decision-making,” n.d.)	No theoretical model	journey	Trust	Anthropomorphism	-0.301	368	0.021
			Trust	Advice Elaboration	0.3721	368	0.0484
			Concern	Anthropomorphism	-0.2700	368	0.039
(Gupta, 2024)	AIDUA	Organization	Intention to Recommend	Emotions	0.29	482	0.023
			Attitude	Perceived	0.24	482	0.039

			Attitude	Usefulness (PU) Perceived Ease of Use (PEU)	0.19	482	0.041
			Attitude	Perceived Enjoyment	0.22	482	0.026
(Xiaohong et al., n.d.)	Extended UTAUT	Education	Behavioral Intention	Performance Expectancy(PE)	-0.026	299	0.731
			Behavioral Intention	Effort Expectancy(EE)	0.18	299	0.036
			Behavioral Intention	Social Influence（SI）	0.079	299	0.256
			Behavioral Intention	Facilitating Conditions(FC)	0.25	299	0.002
			Behavioral Intention	Perceived Enjoyment	0.374	299	<0.001
			Behavioral Intention	Performance Expectancy(PE)	0.392	347	<0.001
			Behavioral Intention	Effort Expectancy(EE)	0.343	347	<0.001
			Behavioral Intention	Social Influence（SI）	0.338	347	<0.001
			Behavioral Intention	Facilitating Conditions(FC)	0.163	347	0.041
			Behavioral Intention	Perceived Interest (PP)	-0.13	347	0.062
(Almahri et al., 2020)	UTAUT2	Education	Behavioral Intention	Performance Expectancy(PE)	0.497	431	0
			Behavioral Intention	Effort Expectancy(EE)	0.107	431	0.018
			Behavioral Intention	Habit	0.266	431	0
			Use Behavior(UB)	Performance Expectancy(PE)	0.679	431	0
(Park and Kim, 2023)	TAM and UGT	Healthcare	Use Behavior(UB)	Perceived usefulness(PU)	0.37	278	<0.001
			Behavioral Intention	Parasocial Interactions	0.46	278	<0.001
			Behavioral Intention	Depression Levels	0.10	278	<0.05
(J. Ma et al. 2024)	TAM	Service	Behavioral Intention	Perceived Usefulness (PU)	0.227	784	<0.01
			Behavioral Intention	Perceived Ease of Use (PEU)	0.304	784	<0.001
			Use Behavior(UB)	Behavioural Intention	0.639	784	<0.001
			Use Behavior(UB)	Perceived Usefulness (PU)	0.145	784	0.015
			Use Behavior(UB)	Perceived Ease of Use (PEU)	0.306	784	<0.001
(Gupta 2024)	Generative Artificial Intelligence Technology	Organization	Attitude	Perceived Usefulness (PU)	0.24	482	0.039

Adoption Model						
	Organization	Attitude	Perceived Ease of Use (PEU)	0.19	482	0.041
		Attitude	Perceived Enjoyment	0.22	482	0.026
		Switching Intentions	Emotions	-0.29	482	0.023

References

(Adžić et al. 2024) Adžić, S., Tot, T. S., Vukovic, V., Radanov, P., & Avakumović, J. (2024). Understanding Student Attitudes toward GenAI Tools: A Comparative Study of Serbia and Austria. International Journal of Cognitive Research in Science, Engineering & Education (IJCRSEE), 12(3).

(Agrawal 2023) Agrawal, K. P. 2023. Towards Adoption of Generative AI in Organizational Settings. J. Comput. Inf. Syst. https://doi.org/10.1080/08874417.2023.2240744

(Al-Abdullatif 2024) Al-Abdullatif, A.M. Modeling Teachers' Acceptance of Generative Artificial Intelligence Use in Higher Education: The Role of AI Literacy, Intelligent TPACK, and Perceived Trust. Educ. Sci. 2024, 14, 1209. https://doi.org/10.3390/educsci14111209

(Almahri et al. 2020) Almahri, F.A.J., Bell, D., Merhi, M. 2020. Understanding Student Acceptance and Use of Chatbots in the United Kingdom Universities: A Structural Equation Modelling Approach, in: 2020 6th International Conference on Information Management (ICIM). Presented at the 2020 6th International Conference on Information Management (ICIM), pp. 284–288. https://doi.org/10.1109/ICIM49319.2020.244712

(Almufarreh 2024) Almufarreh, A. 2024. Almufarreh, Ahmad. 2024. Determinants of Students' Satisfaction with AI Tools in Education: A PLS-SEM-ANN Approach. Sustainability 16: 5354. Available online: https://webofscience.clarivate.cn/wos/alldb/full-record/WOS:001266597300001 (accessed on 25 August 2024).

(Al-Qaysi et al. 2024) Al-Qaysi, N., Al-Emran, M., Al-Sharafi, M.A., Iranmanesh, M., Ahmad, A., Mahmoud, M.A. 2024. Determinants of ChatGPT Use and its Impact on Learning Performance: An Integrated Model of BRT and TPB. Int. J. Hum.-Comput. Interact. https://doi.org/10.1080/10447318.2024.2361210

(Amaro et al. 2024) Amaro, I., Barra, P., Greca, A.D., Francese, R., Tucci, C. 2024. Believe in Artificial Intelligence? A User Study on the ChatGPT's Fake Information Impact. IEEE Transactions on Computational Social Systems 11 5168–5177. https://doi.org/10.1109/TCSS.2023.3291539

(Amoozadeh et al 2024) Amoozadeh et al. 2024. Amoozadeh, Matin, David Daniels, Daye Nam, Aayush Kumar, Stella Chen, Michael Hilton, Sruti Srinivasa Ragavan, and Mohammad Amin Alipour. 2024. Trust in Generative AI among Students. Paper presented at the 55th ACM Technical Symposium on Computer Science Education, SIGCSE 2024, Portland, OR, USA, March 20–23. New York: Assoc Computing Machinery, vol. 1, pp. 67–73. https://doi.org/10.1145/3626252.3630842.

(Andrews et al. 2021) Andrews, J. E., Ward, H., & Yoon, J. (2021). UTAUT as a model for understanding intention to adopt AI and related technologies among librarians. The Journal of Academic Librarianship, 47(6) 102437.

(Avci 2024) Avci, U. 2024. Students' GAI Acceptance: Role of Demographics, Creative Mindsets, Anxiety, Attitudes. JOURNAL OF COMPUTER INFORMATION SYSTEMS. https://doi.org/10.1080/08874417.2024.2386545

(Baek and Kim 2023) Baek, T.H., Kim, M. 2023. Is ChatGPT scary good? How user motivations affect creepiness and trust in generative artificial intelligence. Telemat. Inform. 83 102030. https://doi.org/10.1016/j.tele.2023.102030

(Balakrishnan et al. 2022) Balakrishnan, J., Abed, S. S., & Jones, P. (2022). The role of meta-UTAUT factors, perceived anthropomorphism, perceived intelligence, and social self-efficacy in chatbot-based services?. Technological Forecasting and Social Change, 180 121692.

(Ballesteros et al. 2024) Ballesteros, M. A. A., Enríquez, B. G. A., Farroñán, E. V. R., Juárez, H. D. G., Salinas, L. E. C., Sánchez, J. E. B., ... & Chilicaus, G. C. F. (2024). The Sustainable Integration of AI in Higher Education: Analyzing ChatGPT Acceptance Factors Through an Extended UTAUT2 Framework in Peruvian Universities. Sustainability, 16(23), 1-28.

(Bhaskar et al. 2024) Bhaskar, P., Misra, P., Chopra, G. 2024. Shall I use ChatGPT? A study on perceived trust and perceived risk towards ChatGPT usage by teachers at higher education institutions. INTERNATIONAL JOURNAL OF INFORMATION AND LEARNING TECHNOLOGY 41, 428–447. https://doi.org/10.1108/IJILT-11-2023-0220

(Bhat et al. 2024) Bhat, M.A., Tiwari, C.K., Bhaskar, P., Khan, S.T. 2024. Examining ChatGPT adoption among educators in higher educational institutions using extended UTAUT model. Journal of Information, Communication and Ethics in Society 22, 331–353. https://doi.org/10.1108/JICES-03-2024-0033

(Bouteraa et al. 2024) Bouteraa, M., Bin-Nashwan, S.A., Al-Daihani, M., Dirie, K.A., Benlahcene, A., Sadallah, M., Zaki, H.O., Lada, S., Ansar, R., Fook, L.M., Chekima, B. 2024. Understanding the diffusion of AI-generative (ChatGPT) in higher education: Does students' integrity matter? Comput. Hum. Behav. Rep. 14 100402. https://doi.org/10.1016/j.chbr.2024.100402

(Cabero-Almenara et al. 2024) Cabero-Almenara, J., Palacios-Rodríguez, A., Loaiza-Aguirre, M.I., Andrade-Abarca, P.S. 2024. The impact of pedagogical beliefs on the adoption of generative AI in higher education: predictive model from UTAUT2. Front Artif Intell 7 1497705. https://doi.org/10.3389/frai.2024.1497705

(Camilleri 2024) Camilleri, M.A. 2024. Factors affecting performance expectancy and intentions to use ChatGPT: Using SmartPLS to advance an information technology acceptance framework. Technol. Forecast. Soc. Chang. 201 123247. https://doi.org/10.1016/j.techfore.2024.123247

(Chakraborty et al. 2024) Chakraborty, D., Kar, A.K., Patre, S., Gupta, S. 2024. Enhancing trust in online grocery shopping through generative AI chatbots. J. Bus. Res. 180 114737. https://doi.org/10.1016/j.jbusres.2024.114737

(Chan and Hu, 2023) Chan, C. K. Y., & Hu, W. 2023. Students' voices on generative AI: Perceptions, benefits, and challenges in higher education. International Journal of Educational Technology in Higher Education, 20(1), 43.

(Chan and Zhou 2023) Chan, C.K.Y., Zhou, W. 2023. An expectancy value theory (EVT) based instrument for measuring student perceptions of generative AI. Smart Learn. Env. 10, 64. https://doi.org/10.1186/s40561-023-00284-4

(W. Chang and Park 2024) Chang, W., Park, J. 2024. A comparative study on the effect of ChatGPT recommendation and AI recommender systems on the formation of a consideration set. JOURNAL OF RETAILING AND CONSUMER SERVICES 78. https://doi.org/10.1016/j.jretconser.2024.103743

(Y. Chang et al. 2022) Chang, Y., Lee, S., Wong, S.F., Jeong, S. 2022. AI-powered learning application use and gratification: an integrative model. Inf. Technol. People 35 2115–2139. https://doi.org/10.1108/ITP-09-2020-0632

(Chi et al. 2023) Chi, O., Chi, C., Gursoy, D., Nunkoo, R. 2023. Customers? acceptance of artificially intelligent service robots: The influence of trust and culture. INTERNATIONAL JOURNAL OF INFORMATION MANAGEMENT 70. https://doi.org/10.1016/j.ijinfomgt.2023.102623

(Cimino et al. 2024) Cimino, A., Felicetti, A.M., Corvello, V., Ndou, V., Longo, F. 2024. Generative artificial intelligence (AI) tools in innovation management: a study on the appropriation of ChatGPT by innovation managers. Management Decision ahead-of-print. https://doi.org/10.1108/MD-10-2023-1968

(Cintamür 2024) Cintamür, İ.G. 2024. Acceptance of artificial intelligence devices in banking services: moderation role of technology anxiety and risk aversion. International Journal of Bank Marketing ahead-of-print. https://doi.org/10.1108/IJBM-10-2023-0563

(Dahri et al. 2024) Dahri, Nisar Ahmed, Noraffandy Yahaya, Waleed Mugahed Al-Rahmi, Ahmed Aldraiweesh, Uthman Alturki, Sultan Almutairy, Anna Shutaleva, and Rahim Bux Soomro. 2024. Extended TAM Based Acceptance of AI-Powered ChatGPT for Supporting Metacognitive Self-Regulated Learning in Education: A Mixed-Methods Study. Heliyon 10: e29317. Available online: https://webofscience.clarivate.cn/wos/alldb/full-record/WOS:001226756000001 (accessed on 25 August 2024).

(de Andrés-Sánchez and Gené-Albesa 2023) de Andrés-Sánchez, J., & Gené-Albesa, J. (2023). Explaining policyholders' chatbot acceptance with an unified technology acceptance and use of technology-based model. Journal of Theoretical and Applied Electronic Commerce Research, 18(3) 1217-1237.

(de Vreede and de Vreede n.d.) de Vreede, T., de Vreede, G., n.d. The Effect of AI Engagement on Generative AI Adoption.

(Du and Lv 2024) Du, L., Lv, B. 2024. Factors influencing students' acceptance and use generative artificial intelligence in elementary education: an expansion of the UTAUT model. Educ. Inf. Technol. https://doi.org/10.1007/s10639-024-12835-4

(Duong et al. 2024a) Duong, C.D., Nguyen, T.H., Ngo, T.V.N., Dao, V.T., Do, N.D., Pham, T.V. 2024a. Exploring higher education students' continuance usage intention of ChatGPT: amalgamation of the information system success model and the stimulus-organism-response paradigm. The International Journal of Information and Learning Technology 41, 556–584. https://doi.org/10.1108/IJILT-01-2024-0006

(Duong et al. 2024b) Duong, C.D., Nguyen, T.H., Ngo, T.V.N., Pham, T.T.P., Vu, A.T., Dang, N.S. 2024b. Using generative artificial intelligence (ChatGPT) for travel purposes: parasocial interaction and tourists' continuance intention. Tour. Rev. https://doi.org/10.1108/TR-01-2024-0027

(Duong et al. 2023) Duong, C.D., Vu, T.N., Ngo, T.V.N. 2023. Applying a modified technology acceptance model to explain higher education students' usage of ChatGPT: A serial multiple mediation model with knowledge sharing as a moderator. Int. J. Manag. Educ. 21 100883. https://doi.org/10.1016/j.ijme.2023.100883

(Felicetti et al. 2024) Felicetti, A.M., Cimino, A., Mazzoleni, A., Ammirato, S. 2024. Artificial intelligence and project management: An empirical investigation on the appropriation of generative Chatbots by project managers. JOURNAL OF INNOVATION & KNOWLEDGE 9. https://doi.org/10.1016/j.jik.2024.100545

(Figueiredo et al. 2022) Figueiredo, R., Camargo, M. E., Ferreira, J. J., Zhang, J. Z., & Liu, Y. D. (2022). Predicting the Intention to Adopt Innovation in Supply Chain Finance: Determinants of Brazilian FinTech. Journal of Organizational and End User Computing (JOEUC), 35(2), 1-27.URL https://dl.acm.org/doi/10.4018/JOEUC.333689 (accessed 8.25.24).

(Fu et al. 2024) Fu, C.-J., Silalahi, A.D.K., Huang, S.-C., Phuong, D.T.T., Eunike, I.J., Yu, Z.-H. 2024 The (Un)Knowledgeable, the (Un)Skilled? Undertaking Chat-GPT Users' Benefit-Risk-Coping Paradox in Higher Education Focusing on an Integrated, UTAUT and PMT. International Journal of Human–Computer Interaction 0, 1–31. https://doi.org/10.1080/10447318.2024.2365028

(Grassini et al. 2024) Grassini, S., Aasen, M. L., & Møgelvang, A. (2024). Understanding university students' acceptance of ChatGPT: insights from the UTAUT2 model. Applied Artificial Intelligence, 38(1) 2371168.

(Gu et al. 2024) Gu, C., Jia, S., Lai, J., Chen, R., & Chang, X. (2024). Exploring Consumer Acceptance of AI-Generated Advertisements: From the Perspectives of Perceived Eeriness and Perceived Intelligence. Journal of Theoretical and Applied Electronic Commerce Research, 19(3) 2218-2238.

(Gupta 2024) Gupta, V. 2024. An Empirical Evaluation of a Generative Artificial Intelligence Technology Adoption Model from Entrepreneurs' Perspectives. Systems-Basel 12, 103. https://doi.org/10.3390/systems12030103

(Gursoy et al. 2019) Gursoy, D., Chi, O.H., Lu, L., Nunkoo, R. 2019. Consumers acceptance of artificially intelligent (AI) device use in service delivery. International Journal of Information Management 49, 157–169. https://doi.org/10.1016/j.ijinfomgt.2019.03.008

(Hazaimeh and Al-Ansi 2024) Hazaimeh, M., Al-Ansi, A.M. 2024. Model of AI acceptance in higher education: arguing teaching staff and students perspectives. The International Journal of Information and Learning Technology 41, 371–393. https://doi.org/10.1108/IJILT-01-2024-0005

(Hernandez et al. 2023) Hernandez, A.A., Abisado, M.B., Rodriguez, R.L., Imperial, J.M.R. 2023. Predicting the Use Behavior of Higher Education Students on ChatGPT: Evidence from the Philippines, in: 2023 IEEE INTERNATIONAL CONFERENCE ON TEACHING, ASSESSMENT AND LEARNING FOR ENGINEERING, TALE, Proceedings of IEEE International Conference on Teaching Assessment and Learning for Engineering. Presented at the IEEE International Conference on Teaching, Assessment and Learning for Engineering (IEEE TALE), IEEE, New York, pp. 266–272. https://doi.org/10.1109/TALE56641.2023.10398324

(Hidayat-ur-Rehman and Ibrahim 2023) Hidayat-ur-Rehman, I., Ibrahim, Y. 2023. Exploring factors influencing educators' adoption of ChatGPT: a mixed method approach. Interact. Technol. Smart Educ. https://doi.org/10.1108/ITSE-07-2023-0127

(Hou et al. 2024) Hou, T., Li, M., Tan, Y., & Zhao, H. 2024. Physician adoption of AI assistant.Manufacturing & Service Operations Management,26(5) 1639-1655. https://doi.org/10.1287/msom.2023.0093.

(Hsiao et al. 2024) Hsiao, C. H., & Tang, K. Y. (2024). Beyond acceptance: an empirical investigation of technological, ethical, social, and individual determinants of GenAI-supported learning in higher education. Education and Information Technologies, 1-26.

(F. Huang et al. 2024) Huang, F., Wang, Y., & Zhang, H. (2024). Modelling Generative AI Acceptance, Perceived Teachers' Enthusiasm and Self-Efficacy to English as a Foreign Language Learners' Well-Being in the Digital Era. European Journal of Education, 59(4), e12770.

(K.-L. Huang et al. 2024) Huang, K.-L., Liu, Y., Dong, M.-Q. 2024. Incorporating AIGC into design ideation: A study on self-efficacy and learning experience acceptance under higher-order thinking. Thinking Skills and Creativity 52 101508. https://doi.org/10.1016/j.tsc.2024.101508

(Huynh 2024) Huynh, M.-T. 2024. Huynh, Minh-Tay. "Using generative AI as decision-support tools: unraveling users' trust and AI appreciation." Journal of Decision Systems 2024 : 1-32. https://doi.org/10.1080/12460125.2024.2428166

(Ivanov et al. 2024) Ivanov, S., Soliman, M., Tuomi, A., Alkathiri, N.A., Al-Alawi, A.N. 2024. Drivers of generative AI adoption in higher education through the lens of the Theory of Planned Behaviour. Technology in Society 77. https://doi.org/10.1016/j.techsoc.2024.102521

(Jang 2024) Jang, M. 2024.AI Literacy and Intention to Use Text-Based GenAI for Learning: The Case of Business Students in Korea [WWW Document], n.d. URL https://www.mdpi.com/2227-9709/11/3/54 (accessed 12 May 2025).

(Jang et al. 2024) Jang, S., Lee, H., Kim, Y., Lee, D., Shin, J., Nam, J. 2024. When, What, and how should generative artificial intelligence explain to Users? Telematics and Informatics 93 102175. https://doi.org/10.1016/j.tele.2024.102175

(Jiang et al. 2024) Jiang, Q., Zhang, J., Wang, P. H., & Zhang, Y. (2024). Technology Acceptance and Innovation Diffusion: Are Users More Inclined Toward AIGC-Assisted Design?. International Journal of Human–Computer Interaction, 1-15.

(Joshi 2021) Joshi, H. (2021, October). Perception and Adoption of Customer Service Chatbots among Millennials: An Empirical Validation in the Indian Context. In Webist (Vol. 2021, No. 17, pp. 197-208).

(K. P. Gupta and Pande 2022) Gupta, Kriti Priya, and Smriti Pande. 2022. Indian Customers' Acceptance of Service Robots in Restaurant Services. Behaviour & Information Technology 42: 1946–67. Available online: https://www.tandfonline.com/doi/abs/10.1080/0144929X.2022.2103734 (accessed on 30 August 2024).

(J. S. Kim et al. 2024) Kim, J.S., Kim, M., Baek, T.H. 2024. Enhancing User Experience With a Generative AI Chatbot. Int. J. Hum.-Comput. Interact. https://doi.org/10.1080/10447318.2024.2311971

(Y. Kim et al. 2024) Kim, Y., Blazquez, V., & Oh, T. (2024). Determinants of Generative AI System Adoption and Usage Behavior in Korean Companies: Applying the UTAUT Model. Behavioral Sciences, 14(11) 1035.

(Kocsis and Molnár 2025) Kocsis, Á., Molnár, G. 2025. Factors influencing academic performance and dropout rates in higher education. Oxford Review of Education.

(Korzyński et al. n.d.) Korzyński, P., Silva ,Susana Costa e, Górska ,Anna Maria, and Mazurek, G., n.d. Trust in AI and Top Management Support in Generative-AI Adoption. Journal of Computer Information Systems 0, 1–15. https://doi.org/10.1080/08874417.2024.2401986

(Latikka et al. 2023) Latikka, R., Bergdahl, J., Savela, N., Oksanen, A. 2023. AI as an Artist? A Two-Wave Survey Study on Attitudes Toward Using Artificial Intelligence in Art. Poetics 101 101839. https://doi.org/10.1016/j.poetic.2023.101839

(Lee and Chen 2022) Lee, J.-C., Chen, X. 2022. Exploring users' adoption intentions in the evolution of artificial intelligence mobile banking applications: the intelligent and anthropomorphic perspectives. International Journal of Bank Marketing 40, 631–658. https://doi.org/10.1108/IJBM-08-2021-0394

(L. Li et al. 2024) Li, L., Peng, W., Rheu, M.M.J. 2024. Factors Predicting Intentions of Adoption and Continued Use of Artificial Intelligence Chatbots for Mental Health: Examining the Role of UTAUT Model, Stigma, Privacy Concerns, and Artificial Intelligence Hesitancy. Telemed. e-Health 30, 722–730. https://doi.org/10.1089/tmj.2023.0313

(S. Li et al. 2024) Li, S., Han, R., Fu, T., Chen, M., Zhang, Y. 2024. Tourists' behavioural intentions to use ChatGPT for tour route planning: an extended TAM model including rational and emotional factors. Current Issues in Tourism.

(W. Li et al. 2024) Li, W., Zhang, X., Li, J., Yang, X., Li, D., & Liu, Y. 2024. An explanatory study of factors influencing engagement in AI education at the K-12 Level: an extension of the classic TAM model. Scientific Reports, 14(1) 13922.

(W. Li 2024) Li, Weiyi. 2024. Full Article: A Study on Factors Influencing Designers' Behavioral Intention in Using AI-Generated Content for Assisted Design: Perceived Anxiety, Perceived Risk, and UTAUT. *International Journal of Human–Computer Interaction* 41: 1064–77. Available online: https://www.tandfonline.com/doi/full/10.1080/10447318.2024.2310354 (accessed on 24 August 2024).

(X. Li et al. 2024) Li, X., Shen, L., Ren, X. 2024. Explore the Fashion Industry's Behavioral Intention to Use Artificial Intelligence Generated Content Tools Based on the UTAUT Model. International Journal of Human–Computer Interaction.

(H. Lin et al. 2020) Lin, H., Chi, O., Gursoy, D. 2020. Antecedents of customers' acceptance of artificially intelligent robotic device use in hospitality services. JOURNAL OF HOSPITALITY MARKETING & MANAGEMENT 29, 530–549. https://doi.org/10.1080/19368623.2020.1685053

(H.-L. Lin et al. 2025) Lin, H.-L., Liao, L.-L., Wang, Y.-N., Chang, L.-C. 2025. Attitude and utilization of ChatGPT among registered nurses: A cross-sectional study. Int Nurs Rev 72, e13012. https://doi.org/10.1111/inr.13012

(R. R. Lin et al. 2023) Lin, R. R., Zheng, Y., & Lee, J. C. (2023). Artificial intelligence-based pre-implementation interventions in users' continuance intention to use mobile banking. International Journal of Mobile Communications, 21(4), 518-540.

(Lu et al. 2024) Lu, H., He, L., Yu, H., Pan, T., Fu, K. 2024. A Study on Teachers' Willingness to Use Generative AI Technology and Its Influencing Factors: Based on an Integrated Model. SUSTAINABILITY 16. https://doi.org/10.3390/su16167216

(H. Ma and Li 2024) Ma, H., & Li, N. (2024). Exploring User Behavioral Intentions and Their Relationship With AI Design Tools: A Future Outlook on Intelligent Design | IEEE Journals & Magazine | IEEE Xplore [WWW Document], n.d. URL https://ieeexplore.ieee.org/abstract/document/10632118 (accessed 5.12.25).

(J. Ma et al. 2024) Ma, J., Wang, P., Li, B., Wang, T., Pang, X.S., Wang, D. 2024. Exploring User Adoption of ChatGPT: A Technology Acceptance Model Perspective. Int. J. Hum.-Comput. Interact. https://doi.org/10.1080/10447318.2024.2314358

(Maican et al. 2023) Maican, C.I., Sumedrea, S., Tecau, A., Nichifor, E., Chitu, I.B., Lixandroiu, R., Bratucu, G. 2023. Factors Influencing the Behavioural Intention to Use AI-Generated Images in Business: A UTAUT2 Perspective With Moderators. J. Organ. End User Comput. 35 330019. https://doi.org/10.4018/JOEUC.330019

(Manzoor et al. 2024) Manzoor, S., Ullah, R., Khattak, A., Ullah, M., Han, H. 2024. Exploring tourist perceptions of artificial intelligence devices in the hotel industry: impact of industry 4.0. JOURNAL OF TRAVEL & TOURISM MARKETING 41, 272–291. https://doi.org/10.1080/10548408.2024.2310169

(Marimon et al. 2024) Marimon, F., Mas-Machuca, M., & Akhmedova, A. (2024). Trusting in generative AI: Catalyst for employee performance and engagement in the workplace. International Journal of Human–Computer Interaction, 1-16.

(Marjerison et al. 2022) Marjerison, Rob Kim, Youran Zhang, and Hanyi Zheng. 2022. AI in E-Commerce: Application of the Use and Gratification Model to The Acceptance of Chatbots. Sustainability 14: 14270. https://doi.org/10.3390/su142114270.

(Martínez Puertas et al. 2024) Martínez Puertas, Sergio, María Dolores Illescas Manzano, Cristina Segovia López, and Paulo Ribeiro Cardoso. 2024. Purchase Intentions in a Chatbot Environment: An Examination of the Effects of Customer Experience. Oeconomia Copernicana 15: 145–94.

(Nan et al. 2024) Nan, D., Sun, S., Zhang, S., Zhao, X., Kim, J.H. 2024. Analyzing behavioral intentions toward Generative Artificial Intelligence: the case of ChatGPT. Univers. Access Inf. Soc. https://doi.org/10.1007/s10209-024-01116-z

(Nawaz et al. 2024) Nawaz, S.S., Sanjeetha, M.B.F., Al Murshidi, G., Riyath, M.I.M., Yamin, F.B.M., Mohamed, R. 2024. Acceptance of ChatGPT by undergraduates in Sri Lanka: a hybrid approach of SEM-ANN. Interact. Technol. Smart Educ. https://doi.org/10.1108/ITSE-11-2023-0227

(Ngo et al. 2024) Ngo, T.T.A., Tran, T.T., An, G.K., Nguyen, P.T. 2024. ChatGPT for Educational Purposes: Investigating the Impact of Knowledge Management Factors on Student Satisfaction and Continuous Usage. IEEE Trans. Learn. Technol. 17 1367–1378. https://doi.org/10.1109/TLT.2024.3383773

(Niu et al. 2024) Niu, W., Zhang, W., Zhang, C., Chen, X. 2024. The Role of Artificial Intelligence Autonomy in Higher Education: A Uses and Gratification Perspective. Sustainability 16 1276. https://doi.org/10.3390/su16031276

(Orhan et al. 2024) Orhan, A., Aydın Yıldız, T., & Çınar Yağcı, Ş. (2024). Assessing EFL learners' attitudes on Generative Artificial Intelligence: Development and validation of Generative Artificial Intelligence attitude scale for EFL learners (GenAIAS). Journal of Research on Technology in Education, 1-21.

(Pallivathukal et al. 2024) Pallivathukal, R., Soe, H., Donald, P., Samson, R., Ismail, A. 2024. ChatGPT for Academic Purposes: Survey Among Undergraduate Healthcare Students in Malaysia. CUREUS JOURNAL OF MEDICAL SCIENCE 16. https://doi.org/10.7759/cureus.53032

(Park and Kim 2023) Park, D.Y.Y., Kim, H. 2023. Determinants of Intentions to Use Digital Mental Healthcare Content among University Students, Faculty, and Staff: Motivation, Perceived Usefulness, Perceived Ease of Use, and Parasocial Interaction with AI Chatbot. Sustainability 15, 872. https://doi.org/10.3390/su15010872

(Pellas 2023) Pellas, N. 2023. The influence of sociodemographic factors on students' attitudes toward AI-generated video content creation. Smart Learn. Environ. 10, 57. https://doi.org/10.1186/s40561-023-00276-4

(Pellas 2025) Pellas, N. 2025. The role of students' higher-order thinking skills in the relationship between academic achievements and machine learning using generative AI chatbots. RESEARCH AND PRACTICE IN TECHNOLOGY ENHANCED LEARNING 20. https://doi.org/10.58459/rptel.2025.20036

(Prasad and De 2024) Prasad, K. D. V., & De, T. (2024). Generative AI as a catalyst for HRM practices: mediating effects of trust. Humanities and Social Sciences Communications, 11(1), 1-16. https://www.nature.com/articles/s41599-024-03842-4 (accessed 5.12.25).

(Qiu et al. 2024) Qiu, T., Yang, D., Zeng, H., Chen, X. 2024. Understanding graphic designers' usage behavior of generative artificial intelligence tools. Kybernetes ahead-of-print. https://doi.org/10.1108/K-05-2024-1159

(Rajan and Niranjan 2025) Rajan, S., Niranjan, L.R. 2025. The double-edged sword of ChatGPT: fostering and hindering creativity in postgraduate academics in Bengaluru. INTERNATIONAL JOURNAL OF EDUCATIONAL MANAGEMENT. https://doi.org/10.1108/IJEM-03-2024-0181

(Raman et al. 2024) Raman, R., Mandal, S., Das, P., Kaur, T., Sanjanasri, J.P., Nedungadi, P. 2024. Exploring University Students' Adoption of ChatGPT Using the Diffusion of Innovation Theory and Sentiment Analysis With Gender Dimension. Hum. Behav. Emerg. Tech. 2024 3085910. https://doi.org/10.1155/2024/3085910

(Ramnarain et al. 2024) Ramnarain, U., Ogegbo, A. A., Penn, M., Ojetunde, S., & Mdlalose, N. (2024). Pre-service science teachers' intention to use generative artificial intelligence in inquiry-based teaching. Journal of Science Education and Technology, 1-14.

(Rana et al. 2024) Rana, N.P., Pillai, R., Sivathanu, B., Malik, N. 2024. Assessing the nexus of Generative AI adoption, ethical considerations and organizational performance. Technovation 135 103064. https://doi.org/10.1016/j.technovation.2024.103064

(Russo 2024) Russo, D. 2024. Navigating the Complexity of Generative AI Adoption in Software Engineering. ACM TRANSACTIONS ON SOFTWARE ENGINEERING AND METHODOLOGY 33. https://doi.org/10.1145/3652154

(Salem et al. 2024) Salem, G., El-Gazar, H., Mahdy, A., Alharbi, T., Zoromba, M. 2024. Nursing Students' Personality Traits and Their Attitude toward Artificial Intelligence: A Multicenter Cross-Sectional Study. JOURNAL OF NURSING MANAGEMENT 2024. https://doi.org/10.1155/2024/6992824

(Salifu et al. 2024) Salifu, I., Arthur, F., Arkorful, V., Abam Nortey, S., & Solomon Osei-Yaw, R. (2024). Economics students' behavioural intention and usage of ChatGPT in higher education: a hybrid structural equation modelling-artificial neural network approach. *Cogent Social Sciences*,*10*(1) 2300177.

(M. Shahzad et al. 2024) Shahzad, M., Xu, S., Javed, I. 2024. ChatGPT awareness, acceptance, and adoption in higher education: the role of trust as a cornerstone. INTERNATIONAL JOURNAL OF EDUCATIONAL TECHNOLOGY IN HIGHER EDUCATION 21. https://doi.org/10.1186/s41239-024-00478-x

(M. F. Shahzad et al. 2024) Shahzad, M.F., Xu, S., Zahid, H. 2024. Exploring the impact of generative AI-based technologies on learning performance through self-efficacy, fairness & ethics, creativity, and trust in higher education. EDUCATION AND INFORMATION TECHNOLOGIES. https://doi.org/10.1007/s10639-024-12949-9

(Shen et al. 2024) Shen, X., Mo, X., Xia, T. 2024. Exploring the attitude and use of GenAI-image among art and design college students based on TAM and SDT. Interact. Learn. Environ. https://doi.org/10.1080/10494820.2024.2365959

(Skripchuk 2024) Skripchuk, J., Bacher, J., & Price, T. (2024, August). An Investigation of the Drivers of Novice Programmers' Intentions to Use Web Search and GenAI. In Proceedings of the 2024 ACM Conference on International Computing Education Research-Volume 1 (pp. 487-501).

(Sobaih et al. 2024) Sobaih, A. E. E., Elshaer, I. A., & Hasanein, A. M. (2024). Examining students' acceptance and use of ChatGPT in Saudi Arabian higher education. European Journal of Investigation in Health, Psychology and Education, 14(3), 709-721.

(Sohn and Kwon 2020) Sohn, K., Kwon, O. 2020. Technology acceptance theories and factors influencing artificial Intelligence-based intelligent products. Telematics and Informatics 47 101324. https://doi.org/10.1016/j.tele.2019.101324

(Sohn et al. 2021) Sohn, K., Sung, C., Koo, G., Kwon, O. 2021. Artificial intelligence in the fashion industry: consumer responses to generative adversarial network (GAN) technology. INTERNATIONAL JOURNAL OF RETAIL & DISTRIBUTION MANAGEMENT 49, 61–80. https://doi.org/10.1108/IJRDM-03-2020-0091

(Spatscheck et al. 2024) Spatscheck, N., Schaschek, M., & Winkelmann, A. (2024). The effects of generative AI's human-like competencies on clinical decision-making. Journal of Decision Systems, 1-39.

(Stevens and Stetson 2023) Stevens, A.F., Stetson, P. 2023. Theory of trust and acceptance of artificial intelligence technology (TrAAIT): An instrument to assess clinician trust and acceptance of artificial intelligence. J. Biomed. Inform. 148 104550. https://doi.org/10.1016/j.jbi.2023.104550

(Sudan et al. 2024) Sudan, T., Hans, A., Taggar, R. 2024. Transformative learning with ChatGPT: analyzing adoption trends and implications for business management students in India. Interactive Technology and Smart Education ahead-of-print. https://doi.org/10.1108/ITSE-10-2023-0202

(Sun et al. 2022) Sun, J., Gu, C., Chen, J., Wei, W., Yang, C., & Jiang, Q. (2022, June). A study of the effects of interactive AI image processing functions on children's painting education. In International Conference on Human-Computer Interaction(pp. 93-108). Cham: Springer International Publishing.

(Syed et al. 2024) Syed, W., Bashatah, A., Alharbi, K., Bakarman, S.S., Asiri, S., Alqahtani, N. 2024. Awareness and Perceptions of ChatGPT Among Academics and Research Professionals in Riyadh, Saudi Arabia: Implications for Responsible AI Use. Med Sci Monit 30, e944993. https://doi.org/10.12659/MSM.944993

(Tanantong and Wongras 2024) Tanantong, T., & Wongras, P. 2024. A UTAUT-based framework for analyzing users' intention to adopt artificial intelligence in human resource recruitment: a case study of Thailand. Systems, 12(1), 28.

(Tang and Su 2025) Tang, Y., & Su, L. (2025). Graduate education in China meets AI: Key factors for adopting AI-generated content tools. Libri, 75(1), 81-96. https://www.degruyterbrill.com/document/doi/10.1515/libri-2024-0079/html (accessed 5.12.25).

(Thongsri et al. 2024) Thongsri, N., Tripak, O., Bo, Y. 2024. Do learners exhibit a willingness to use ChatGPT? An advanced two-stage SEM-neural network approach for forecasting factors influencing ChatGPT adoption. Interact. Technol. Smart Educ. https://doi.org/10.1108/ITSE-01-2024-0001

(Tian et al. 2024) Tian, W., Ge, J., Zhao, Y., Zheng, X. 2024. AI Chatbots in Chinese higher education: adoption, perception, and influence among graduate students—an integrated analysis utilizing UTAUT and ECM models. Front. Psychol. 15. https://doi.org/10.3389/fpsyg.2024.1268549

(Tiwari et al. 2023) Tiwari, C.K., Bhat, M.A., Khan, S.T., Subramaniam, R., Khan, M.A.I. 2023. What drives students toward ChatGPT? An investigation of the factors influencing adoption and usage of ChatGPT. Interact. Technol. Smart Educ. 708854. https://doi.org/10.1108/ITSE-04-2023-0061

(Vitezić and Perić 2021) Vitezić, V., Perić, M. 2021. Artificial intelligence acceptance in services: connecting with Generation Z. The Service Industries Journal.

(C. Wang 2024) Wang, C. 2024. Art Innovation or Plagiarism? Chinese Students' Attitudes Toward AI Painting Technology and Influencing Factors. IEEE Access 12 85795–85805. https://doi.org/10.1109/ACCESS.2024.3412176

(C. Wang et al. 2024) Wang, Chenghao, Zou, B., Du, Y., Wang, Z. 2024. The impact of different conversational generative AI chatbots on EFL learners: An analysis of willingness to communicate, foreign language speaking anxiety, and self-perceived communicative competence. System 127 103533. https://doi.org/10.1016/j.system.2024.103533

(C. L. Wang et al. 2024) Wang, C. L., Wang, H., Li, Y., Dai, J., Gu, X., Yu, T. 2024. Factors Influencing University Students' Behavioral Intention to Use Generative Artificial Intelligence: Integrating the Theory of Planned Behavior and AI Literacy. INTERNATIONAL JOURNAL OF HUMAN-COMPUTER INTERACTION. https://doi.org/10.1080/10447318.2024.2383033

(F. Wang et al. 2023) Wang, F., King, R.B., Chai, C.S., Zhou, Y. 2023. University students' intentions to learn artificial intelligence: the roles of supportive environments and expectancy–value beliefs. Int J Educ Technol High Educ 20, 51. https://doi.org/10.1186/s41239-023-00417-2

(K. Wang et al. 2024) Wang, K., Ruan, Q., Zhang, X., Fu, C., Duan, B. 2024. Pre-Service Teachers' GenAI Anxiety, Technology Self-Efficacy, and TPACK: Their Structural Relations with Behavioral Intention to Design GenAI-Assisted Teaching. Behav. Sci. 14, 373. https://doi.org/10.3390/bs14050373

(S. F. Wang and Chen 2024a) Wang, S. F., & Chen, C. C. (2024a). Exploring designer trust in artificial intelligence-generated content: TAM/TPB model study. Applied Sciences, 14(16) 6902. https://doi.org/10.3390/app14166902

(S. F. Wang and Chen 2024b) Wang, S. F., & Chen, C. C. 2024b. Explore the driving factors of designers' AIGC usage behavior based on SOR framework. Frontiers in Computer Science, 6 1417016.

(X. Wang and Wang 2024) Wang, X., & Wang, Y. 2024. Analysis of trust factors for AI-assisted diagnosis in intelligent Healthcare: Personalized management strategies in chronic disease management. Expert Syst. Appl. 255 124499. https://doi.org/10.1016/j.eswa.2024.124499

(Y. Wang and Zhang 2023) Wang, Y., Zhang, W. 2023. Factors Influencing the Adoption of Generative AI for Art Designing Among Chinese Generation Z: A Structural Equation Modeling Approach. IEEE Access 11 143272–143284. https://doi.org/10.1109/ACCESS.2023.3342055

(Y. Wang et al. 2025) Wang, Y., Zhao, Y., Tian, X., Yang, J., Luo, S. 2025. The influence of subjective knowledge, technophobia and perceived enjoyment on design students' intention to use artificial intelligence design tools. Int J Technol Des Educ 35, 333–358. https://doi.org/10.1007/s10798-024-09897-3

(Y. Wang et al. 2024) Wang, Yabing, Kelly Shu-Xia Liu, Yi Zheng, and Michael Yi-Chao Jiang. 2024. Examining the Moderating Effect of Motivation on Technology Acceptance of Generative AI for English as a Foreign Language Learning. Education and Information Technologies 29: 23547–75. Available online: https://link.springer.com/article/10.1007/s10639-024-12763-3 (accessed on 24 August 2024).

(Wong et al. 2023) Wong, I., Zhang, T., Lin, Z., Peng, Q. 2023. Hotel AI service: Are employees still needed? JOURNAL OF HOSPITALITY AND TOURISM MANAGEMENT 55, 416–424. https://doi.org/10.1016/j.jhtm.2023.05.005

(Wu et al. 2022) Wu, W., Zhang, B., Li, S., Liu, H. 2022. Exploring Factors of the Willingness to Accept AI-Assisted Learning Environments: An Empirical Investigation Based on the UTAUT Model and Perceived Risk Theory. Front. Psychol. 13. https://doi.org/10.3389/fpsyg.2022.870777

(Xia and Chen 2024) Xia, Y., Chen, Y. 2024. Driving Factors of Generative AI Adoption in New Product Development Teams from a UTAUT Perspective. INTERNATIONAL JOURNAL OF HUMAN-COMPUTER INTERACTION. https://doi.org/10.1080/10447318.2024.2375686

(Xiaohong et al. n.d.) Xiaohong, L., Jun, Z., Xiaoming, C., Beina, Z. n.d. A study on behavioral intentions of artificial intelligence learning platform: comparing the perspectives of teachers and students. Interactive Learning Environments 0, 1–21. https://doi.org/10.1080/10494820.2024.2343752

(Xie et al. 2024) Xie, C., Wang, Y., & Cheng, Y. (2024). Does artificial intelligence satisfy you? A meta-analysis of user gratification and user satisfaction with AI-powered chatbots. International Journal of Human–Computer Interaction, 40(3), 613-623.

(Xiong et al. 2024) Xiong, Y., Shi, Y., Pu, Q., & Liu, N. (2024). More trust or more risk? User acceptance of artificial intelligence virtual assistant. Human Factors and Ergonomics in Manufacturing & Service Industries, 34(3), 190-205.

(B. Yang et al. 2024) Yang, B., Sun, Y., Li, Q. 2024. To Be Credible or to Be Creative? Understanding the Antecedents of User Satisfaction with AI-Generated Content from a Cognitive Fit Perspective. Presented at the Hawaii International Conference on System Sciences. https://doi.org/10.24251/HICSS.2024.049

(Y. Yang et al. 2025) Yang, Y., Xia, Q., Liu, C., & Chiu, T. K. (2025). The impact of TPACK on teachers' willingness to integrate generative artificial intelligence (GenAI): The moderating role of negative emotions and the buffering effects of need satisfaction. Teaching and Teacher Education, 154 104877.

(Yao and Abd Halim 2024) Yao, N., Abd Halim, N.D. 2024. Analyzing Factors Influencing Primary School Teachers' Acceptance Willingness of Artificial Intelligence Technology, in: Proceedings of the 2023 6th International Conference on Educational Technology Management, ICETM '23. Association for Computing Machinery, New York, NY, USA, pp. 35–41. https://doi.org/10.1145/3637907.3637951

(Yildiz Durak et al. 2024) Yildiz Durak, H., & Onan, A. (2024). Predicting the use of chatbot systems in education: a comparative approach using PLS-SEM and machine learning algorithms. Current Psychology, 43(28) 23656-23674.

(Yilmaz et al. 2023) Yilmaz, F.G.K., Yilmaz, R., Ceylan, M. 2023. Generative Artificial Intelligence Acceptance Scale: A Validity and Reliability Study. Int. J. Hum.-Comput. Interact. https://doi.org/10.1080/10447318.2023.2288730

(Yin et al. 2023) Yin, M., Han, B., Ryu, S., Hua, M. 2023. Acceptance of Generative AI in the Creative Industry: Examining the Role of AI Anxiety in the UTAUT2 Model, in: Degen, H., Ntoa, S., Moallem, A. (Eds.), HCI INTERNATIONAL 2023 LATE BREAKING PAPERS, HCII 2023, PT VI, Lecture Notes in Computer Science. Presented at the 25th International Conference on Human-Computer Interaction (HCI International), Springer International Publishing Ag, Cham, pp. 288–310. https://doi.org/10.1007/978-3-031-48057-7_18

(Yu et al. 2024) Yu, X., Yang, Y., Li, S. 2024. Users' continuance intention towards an AI painting application: An extended expectation confirmation model. PLOS ONE 19, e0301821. https://doi.org/10.1371/journal.pone.0301821

(Yusuf et al. 2024) Yusuf, A., Pervin, N., Roman-Gonzalez, M. 2024. Generative AI and the future of higher education: a threat to academic integrity or reformation? Evidence from multicultural perspectives. Int. J. Educ. Technol. High. Educ. 21, 21. https://doi.org/10.1186/s41239-024-00453-6

(Zhang et al. 2024) Zhang, X., Yu, P., Ma, L., Liang, Y. 2024. How the Human-Like Characteristics of AI Assistants Affect Employee Creativity: A Social Network Ties Perspective. INTERNATIONAL JOURNAL OF HUMAN-COMPUTER INTERACTION. https://doi.org/10.1080/10447318.2024.2379719

(Zhao et al. 2024) Zhao, L., Rahman, M. H., Yeoh, W., Wang, S., & Ooi, K. B. (2024). Examining factors influencing university students' adoption of generative artificial intelligence: a cross-country study. Studies in Higher Education, 1-23.

(Zhou and Zhang 2024a) Zhou, T., Zhang, C. 2024a. Examining Generative AI User Intermittent Discontinuance from a C-A-C Perspective. Int. J. Hum.-Comput. Interact. https://doi.org/10.1080/10447318.2024.2376370

(Zhou and Zhang 2024b) Zhou, T., Zhang, C. 2024b. Examining generative AI user addiction from a C-A-C perspective. Technology in Society 78 102653. https://doi.org/10.1016/j.techsoc.2024.102653

(Zhu et al. 2024) Zhu, W., Huang, L., Zhou, X., Li, X., Shi, G., Ying, J., Wang, C. 2024. Could AI Ethical Anxiety, Perceived Ethical Risks and Ethical Awareness About AI Influence University Students' Use of Generative AI Products? An Ethical Perspective. Int. J. Hum.-Comput. Interact. https://doi.org/10.1080/10447318.2024.2323277

(Ziemba et al. 2023) Ziemba, E. W., Maruszewska, E. W., Grabara, D., & Renik, K. 2023, September. Acceptance and Use of ChatGPT among accounting and finance higher education students. In European Conference on Artificial Intelligence (pp. 185-202). Cham: Springer Nature Switzerland.
